# Inference on the dynamics of COVID-19 in the United States

**DOI:** 10.1038/s41598-021-04494-z

**Published:** 2022-02-10

**Authors:** Satarupa Bhattacharjee, Shuting Liao, Debashis Paul, Sanjay Chaudhuri

**Affiliations:** 1 Department of Statistics, University of California, Davis, 95616 USA; 2 Graduate Group in BioStatistics, University of California, Davis, 95616 USA; 3 Department of Statistics and Applied Probability, National University of Singapore, Singapore, 117546 Singapore

**Keywords:** Statistics, Epidemiology

## Abstract

The evolution of the COVID-19 pandemic is described through a time-dependent stochastic dynamic model in discrete time. The proposed multi-compartment model is expressed through a system of difference equations. Information on the social distancing measures and diagnostic testing rates are incorporated to characterize the dynamics of the various compartments of the model. In contrast with conventional epidemiological models, the proposed model involves interpretable temporally static and dynamic epidemiological rate parameters. A model fitting strategy built upon nonparametric smoothing is employed for estimating the time-varying parameters, while profiling over the time-independent parameters. Confidence bands of the parameters are obtained through a residual bootstrap procedure. A key feature of the methodology is its ability to estimate latent unobservable compartments such as the number of asymptomatic but infected individuals who are known to be the key vectors of COVID-19 spread. The nature of the disease dynamics is further quantified by relevant epidemiological markers that make use of the estimates of latent compartments. The methodology is applied to understand the true extent and dynamics of the pandemic in various states within the United States (US).

## Introduction

The novel coronavirus has been ravaging the world since early 2020. First identified in Wuhan, Hubei Province, China, the epidemic has since spread to every corner of the world. As of February 5, 2021^1^, more than 105 million people have been infected, out of which more than 2.1 million have died of the disease. The World Health Organization declared the situation a pandemic on March 11, 2020. Since then, various parts of the world have gone through multiple waves surges in the number of new infections. The pandemic has severely affected the world economy. Repeated lock-downs, travel restrictions, and other measures of containment have severely impacted the economy of many countries, stretched healthcare systems to the extreme, and caused mental health crises for large chunks of the population.

The new pathogen (SARS-CoV-2) that causes the disease^2^ is mostly unknown in terms of its infectivity and clinical profile. It is well-known that the infection primarily spreads through infected but asymptomatic people^3–5^. The number of such people remains unknown. The reported number is based on symptomatic or positively tested persons, which grossly underestimates the true value. Because of the undetermined denominator effect, important epidemiological markers like the death rate, hospitalization rate etc remain non-determinable from the observed data. Various estimates^6–10^ of these markers have been postulated by many authors. Mathematical modeling and quantification of the epidemiological parameters^11–16^ of the pandemic have been crucial in understanding and interpreting the transmission dynamics from the perspective of public health researchers and policymakers around the globe^17–20^. The dynamics of COVID-19 in various states of the United States (US) has been studied by several authors^21,22^. We analyze such publicly available state-wise COVID-19 data from the US using the proposed methodology.

A number of popular compartmental epidemiological models, such as SIR (Susceptible-Infectious-Recovered) model, SEIR (Susceptible-Exposed-Infectious-Recovered) model, and SIRD (Susceptible-Infectious-Recovered-Deceased) model, have been employed to describe the dynamics of COVID-19^23–26^. Such models yield estimates of epidemiological markers such as the basic reproduction number ($R_0$), and various doubling and case fatality rates that are indicators of the disease growth pattern^27,28^. Prediction of epidemiological characteristics and transmission patterns in this context have also attracted major attention^29–32^. Advanced statistical methods have been employed in forecasting the number of cases worldwide^33^ or quantifying the effects of prevention mechanisms like social distancing^34–39^, public gathering, and travel restrictions^40–42^ for various countries. Due to the difference in analytical methods and assumptions, the parameter estimates describing COVID-19 dynamics vary widely. This variability is also reflected in the estimates of the effectiveness of public health interventions implemented worldwide. Most epidemiological models of disease transmission are simplistic and use time-invariant transmission rates. However, in reality, due to mitigation efforts and the evolving nature of the infection mechanism, such rates become temporally dynamic. Furthermore, most SEIR-type models exclude the effects of testing and subsequent quarantining, and occasionally, even hospitalization. Such practices fail to adequately account for the size of the susceptible population and therefore tend to provide unreliable estimates of the number of asymptomatic persons infected by COVID-19 in the population.

We propose a detailed discrete-time semiparametric stochastic dynamic model for COVID-19 spread. The model is expressed through a system of difference equations connecting various interpretable compartments in the disease dynamics such as individuals who are susceptible, asymptomatic but infected, quarantined, hospitalized, dead, and have recovered from the disease. The model has interpretable time-varying parameters that reflect various temporally dynamic rates. The model also includes available information on the number of tests. On the other hand, the proposed model does not make restrictive and often untestable distributional assumptions about compartments or parameters that are commonplace in various probabilistic models for the epidemiological dynamics.

We employ nonlinear nonparametric regression techniques through a profiling-based estimation procedure to estimate the model parameters and the number of people in different compartments. Using residual bootstrap based techniques, we also provide point-wise confidence intervals (bands) for the time-invariant (time-varying) parameters. The proposed model and estimation procedure relies on linear kernel weighting and fairly low dimensional optimization, thus avoiding Markov chain Monte Carlo and other computationally expensive methods employed by Bayesian inference schemes for standard epidemiological models. Therefore, the estimates can be obtained almost instantaneously. Another key feature of this method is the ability of identifying and estimating unobservable quantities such as the actual number of asymptomatic but infected people at any given time. The estimated trajectory of the infected but asymptomatic population over time, its doubling rate, the true case fatality rate, and an analogue of the basic reproduction rate are crucial in interpreting the time-dynamics of the pandemic. They have important implications for policy decisions regarding appropriate mitigation strategies.

The contributions here are significant for the following reasons. Since the number of infected but asymptomatic individuals is unknown, conventional epidemiological models of disease spread do not readily apply to the COVID-19 dynamics. The adaption of these models to COVID-19 spread necessitates strong assumptions and costly numerical computations. Our proposed model provides a computationally inexpensive method for estimating several unobserved states as well as relevant parameters governing the spread of the disease. Various epidemiological markers based on these estimates are introduced to reveal the true extent of the pandemic in the US.

## A multi-compartment model for disease spread

Throughout, a closed population without emigration or immigration is assumed. The model describes the spread of the COVID19 pandemic in terms of various observable and partially or totally unobservable compartments.

Suppose at time *t*, $C_t$, $D_t$, $T_t$, respectively, denote the number of confirmed cases, the number of deaths due to the disease and the number of tests performed up to time *t*. These variables are nondecreasing cumulative counts and are generally fully observed. The number of hospitalized persons due to COVID-19 infection at time *t* (denoted $H_t$) is also generally observed (see “Results” section for more detail). Furthermore, we observe $Q_t$, the number of asymptomatic individuals who are in quarantine at time *t*. These individuals have been tested positive, but show no significant symptoms requiring hospitalization.

The most crucial unobserved compartment is $A_t$, i.e., the number of infected but asymptomatic individuals at time *t*. It is well known that the people in this group are primary spreaders of the disease. Furthermore, due to underreporting, the number of confirmed cases would be a fraction of $A_t$. Since we do not observe how many in the population are currently infected, the number of susceptible individuals at time *t*, (denoted $S_t$) is also unobserved.

The number of recovered individuals (denoted $R_t$) up to time *t* can be partially observed. To understand this, note that the recoveries from quarantine centers and hospitals, (denoted $R^Q_t$ and $R^H_t$ respectively) are reported, though not necessarily separately (see Supplement Section S2., for the case when $R^Q_t$ and $R^H_t$ are reported separately). But since $A_t$ is unobserved, the number of asymptomatic but infected people who recover without being quarantined or hospitalized (denoted $R^A_t$) cannot be observed. That is, even though $R^{reported}_t=R^Q_t+R^H_t$ is available from the data, the total recovery $R_t$ is not.

The proposed disease propagation model is based on the following assumptions:

**A1**: Only an asymptomatic individual who is not either in quarantine or in hospital can transmit the disease to a susceptible individual.
**A2**: People who recover from the disease are immune from subsequent infection.
**A3**: The false positive rate for the test is negligible, so that if somebody is confirmed to be positive, then he/she is assumed to be infected.
**A4**: Anybody who shows significant symptoms, whether being in quarantine or not, is immediately hospitalized and is tested to be positive.
**A5**: There is no effective treatment regime for the asymptomatic individuals, and so they recover or turn symptomatic at the same rate regardless of whether they are tested positive (and hence quarantined) or not.

**Figure 1:**
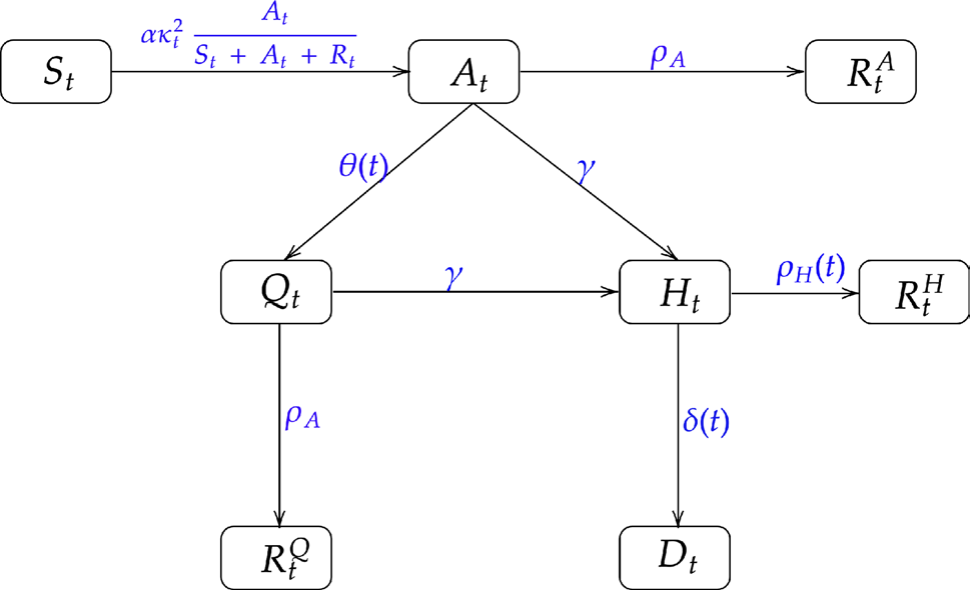
A graphical representation of the disease propagation model. $S_t,\ A_t, \ H_t,\ Q_t,\ D_t$ are the number of susceptible, infected, hospitalized, quarantined, and deceased people at time *t* respectively. $R^Q_t, \ R^H_t,\ R^A_t$ represent the recovered population from quarantined, hospitalization, and infected but asymptotic stages respectively. The rate parameters are as described in “Disease propagation model” section.

A graphical representation of the proposed disease propagation model is presented in Fig. 1 below. The assumptions A1-A5 are quite general and concur to the observed dynamics of the COVID-19 pandemic so far, even though a relatively tiny fraction of people do get infected by prolonged exposure to symptomatic patients, typically in hospitals. However, this small violation of assumption A1 is unlikely to have a significant influence on the overall dynamics, and in any case, the requisite data to account for this violation is practically unavailable. The number of reported reinfection after recovery is negligible, so are the false positive rates of both RT-PCR and antigen tests (estimated to be less than 5%^43–46^). If necessary, the assumptions A2 and A3 can be generalized by adding a fraction of the recovered people in the susceptible category. Assumption A5 implies that the rate of transfer from compartment $A_t$ to $R^A_t$ is the same as that of transfer from the compartments $Q_t$ to $R^Q_t$ and the rate of transfer from the compartments $A_t$ and $Q_t$ to $H_t$ are equal.

### Disease propagation model

We assume an underlying Poisson process model for describing the disease dynamics. Let $\Delta C_{t}=C_{t+1}-C_t$ be the increments in the number of observed confirmed cases in day $t+1$. The increments $\Delta A_{t}$, etc. are defined similarly. Under our model, conditionally on the current values of different compartments (collectively denoted by $\mathscr {F}_t$), the above increments follow Poisson distributions with their mean depending on $\mathscr {F}_t$ and a set of rate parameter. Based on our assumptions, the evolution model is expressed as follows:

1 $$\begin{aligned} \mathbb {E}[\Delta S_{t}|\mathscr {F}_t]&= -\left( \frac{S_t}{S_t + A_t + R_t}\right) \alpha \kappa _t^2 A_t, \end{aligned}$$

2 $$\begin{aligned} \mathbb {E}[\Delta A_{t}|\mathscr {F}_t]&= - (\theta (t) + \gamma + \rho _A) A_t + \left( \frac{S_t}{S_t + A_t + R_t}\right) \alpha \kappa _t^2 A_t, \end{aligned}$$

3 $$\begin{aligned} \mathbb {E}[\Delta Q_{t}|\mathscr {F}_t]&= \theta (t) A_t - (\gamma + \rho _A) Q_t, \end{aligned}$$

4 $$\begin{aligned} \mathbb {E}[\Delta H_{t}|\mathscr {F}_t]&= \gamma (A_t + Q_t) - (\rho _H(t) +\delta (t)) H_t, ~\mathbb {E}[\Delta D_{t}|\mathscr {F}_t] = \delta (t) H_t, \end{aligned}$$

5 $$\begin{aligned} \mathbb {E}[\Delta C_{t}|\mathscr {F}_t]&= (\theta (t) + \gamma ) A_t, \end{aligned}$$

6 $$\begin{aligned} \mathbb {E}[\Delta R_{t}^A|\mathscr {F}_t]&= \rho _A A_t, ~\mathbb {E}[\Delta R_{t}^Q|\mathscr {F}_t] = \rho _A Q_t, ~\mathbb {E}[\Delta R_{t}^H|\mathscr {F}_t] = \rho _H(t) H_t, \end{aligned}$$

7 $$\begin{aligned} \mathbb {E}[\Delta R_{t}|\mathscr {F}_t]&= \mathbb {E}[\Delta R_{t}^H|\mathscr {F}_t] +\mathbb {E}[\Delta R_{t}^Q|\mathscr {F}_t] +\mathbb {E}[\Delta R_{t}^A|\mathscr {F}_t]. \end{aligned}$$

A schematic diagram of the proposed model can be found in Fig. 1. All parameters in the proposed model are non-negative. The parameter $\alpha$ is the baseline infection rate, in the absence of any social distancing. This means, $\alpha$ is the average number of susceptible individuals who may be infected on any given day by an asymptomatic but infected individual. The rate of daily recovery directly from the asymptomatic compartment is denoted by $\rho _A$. By assumption A5, this is also the daily rate at which a quarantined individual directly recovers. We use $\gamma$ to describe the rate at which an asymptomatic individual may become symptomatic on a given day. By assumption A5, this rate is the same whether the individual is free or in quarantine. The symbols, $\rho _H(t)$ and $\delta (t)$, respectively, denote is the rate at which people recover and die from the hospitalized compartment. We assume both these rates to be time-varying to reflect the changing levels of effectiveness of treatment regimes over time. We emphasize that Poisson distributions for the increments of various compartments are only a working assumption that guides our estimation strategy (e.g., by formulating appropriate transformations of variables). In Supplement Sections S6. and S7., we carry out a detailed numerical simulation under the Poisson model to validate the statistical performance of the proposed estimation procedure.

Information about daily tests is included in the model using the function $\theta (t)$. We call it the *confirmed fraction (CF)*, i.e., the fraction of currently asymptomatic individuals who are detected through testing. Parameter $\theta (t)$ would depend on the daily number of tests, as well as the efficiency of the testing strategy in identifying the infected and asymptomatic individuals. It can also be viewed as an intervention parameter, controlling the overall testing rate per hospitalization. The contact tracing strategies were introduced by many states^47,48^ with varying success. In many parts of the world, people in close contact with hospitalized patients are routinely tested. This strategy is closely connected to cluster sampling, where a cluster is defined by the contacts of a hospitalized person.

Guided by the above consideration, we reformulate the parameter $\theta (t)$ by expressing it as follows:

8 $$\begin{aligned} \theta (t) = \phi (t) \frac{\Delta T_t}{H_t}, \end{aligned}$$

where $\phi (t)$ is interpreted as the *testing efficiency (TE)* since it measures the fraction of confirmed asymptomatic cases per test, per (currently) hospitalized patient. We use $\Delta T_t/H_t$ as a surrogate for the contact tracing intensity, since this quantity literally represents the number of new tests on day $t+1$, per hospitalized (and hence severely symptomatic) patient. Clearly, the value of $\theta (t)$ is modulated by this ratio, while the factor $\phi (t)$ implicitly quantifies the extent of positivity among those tested, after accounting for the testing intensity, which justifies the nomenclature “testing efficiency”. Hypothetically, one may aim to estimate $\theta (t)$ in terms of the number of people who have been tested positive. However, in most countries (including the US) contact tracing was limited, making estimation of $\theta (t)$ difficult. A realistic alternative is to estimate $\phi (t)$ instead, which provides an estimate of $\theta (t)$ via (8) and makes our model interpretable and more flexible. Note that we do not assume $\phi (t)$ to be known. It is estimated from data (see “Methods : parameter and compartment estimation” section).

Equations (1) and (2), the parameter $\alpha \kappa _t^2$ approximately measures the daily rate at which a susceptible individual turns asymptomatic-infected. Here, $\alpha$ represents the baseline infection rate, and the $\kappa _t$, which represents the current level of interaction among individuals, is expressed as a fraction, taking value 1 for normal activity, and 0 for complete lockdown. This parameter thus measures the social distancing in the population. In general, $\kappa _t$ is not observable. However, the parameter $\alpha \kappa _t^2$ can be estimated from observed data. Moreover, using our procedure, we also obtain estimates of the key epi-markers ($\gamma$, $\rho _A$, $\rho _H(t)$, $\delta (t)$ and $\theta (t)$) as well as the unobserved state $A_t$, without any knowledge of $\kappa _t$. However, if there is information about the degree of social distancing, then that can be used to estimate $\kappa _t$ and $\alpha$, separately. As for example, the community mobility data collected by Google^34,49–51^ aims to provide insights into what has changed in response to policies aimed at combating COVID-19. This mobility data can be used as a surrogate for $\kappa _t$. The results are presented in Section S.8. of the Supplementary Material. It should be noted that there are alternative data sources on social distancing, such as SafeGraph^52^, Apple^53^, Facebook^54^ etc. that can also be used for this purpose.

In the early stage of the epidemic, the fraction $S_t/(S_t+A_t+R_t) \approx 1$. Furthermore, rather than waiting for herd immunity to be achieved, mitigation measures are implemented in most affected places or countries to contain the spread of the disease. As a consequence, at any given time, the number of non-susceptible people is much lower as compared to the susceptible population. So $S_t/(S_t+A_t+R_t)$ has remained quite close to 1 for almost the duration of the pandemic until this point, due to the absence of mass-scale vaccination.

Notice that Eq. (5), provides a connection between the daily reported confirmed cases $\Delta C_t$ and the number of asymptomatic-infected individuals $A_t$ in the population. In our model, an asymptomatic-infected person can be discovered either through a positive test and subsequent quarantining or through hospitalization upon showing severe symptoms. Therefore, once the estimates of $\theta (t)$ and $\gamma$ are available, Eq. (5) allows us to estimate the unknown $A_t$ from the observed $C_t$. It is also clear that, due to unavoidable severe under-reporting, $\Delta C_t$ will only be a fraction of the number of total infected individuals at any time point.

### Some relevant epidemiological markers

The proposed model is more realistic than the traditional such as SIR model, SEIR model etc., and allows us to estimate different epidemiological markers which can measure the dynamics of disease spread. Our focus here is on estimating epidemiological markers related to the number of asymptomatic but infected persons (i.e. $A_t$) in the population. It is well-known that the disease is mostly spread through persons in that group. Thus the proposed epidemiological markers reveal more fundamental trends of disease dynamics, than what can be obtained only by the confirmed case counts. In particular, we define the following epidemiological markers:

#### Relative change in confirmed fraction (RCCF)

The relative change in confirmed fraction measures the change in the fraction of currently asymptomatic-infected individuals who are caught in the quarantine net through testing relative to the total fraction of currently infected individuals who are either quarantined or hospitalized. From “Disease propagation model” section we get:

9 $$\begin{aligned} RCCF(t) = \frac{\Delta \theta (t)}{\theta (t) + \gamma }. \end{aligned}$$

The above equation is obtained by applying the difference operator on both sides of the equation $\Delta C_t = (\theta (t) + \gamma )A_t$ (see (16)), and subsequently dividing both sides by $\Delta C_t$. The marker *RCCF*(*t*) measures the dynamics of the efficacy of the testing regime to isolate the asymptomatic but infected individuals from the population into quarantine. From Eq. (8), this marker is directly controlled by the prevalent testing strategy and efficiency.

#### Crude infection rate (CIR) and net infection rate (NIR)

The crude infection rate is defined as the fraction of change in the daily confirmed cases on a day to the number of confirmed cases on that day. In our notation, it follows that:

10 $$\begin{aligned} CIR(t) = \frac{\Delta ^2 C_t}{\Delta C_t}. \end{aligned}$$

Since CIR suffers from the under-representation inherent in the reported number of confirmed cases, we define a model-based estimate for the infection rate, denoted Net Infection Rate (NIR), which is the ratio of the daily change in the number of asymptomatic-infected individuals to the number of the asymptomatic-infected individuals. In our notations, from (9), (16), and (17) simple algebraic manipulations yield:

11 $$\begin{aligned} NIR(t) = \frac{\Delta A_t}{A_t}=\frac{CIR(t) - RCCF(t)}{1+ RCCF(t)}. \end{aligned}$$

#### Daily new infections (NI)

From our model and assumptions, the daily number of new infections is given by the number of the susceptible population who turn asymptomatic-infected on that day. From Eq. (1) we define this marker as:

12 $$\begin{aligned} NI(t) = \alpha \kappa _t^2\left( \frac{S_t}{S_t+A_t+R_t}\right) A_t. \end{aligned}$$

The cumulative number of new infections up to time *t* can be defined as $CNI(t)=\sum ^t_{i=1} NI(i)$.

#### Doubling times and rates

The doubling time at time *t*, denoted $t_d(t)$ measures how much longer it would take for the number of infected up to time *t* to double. The doubling rate at time *t*, ${\tilde{\xi }}(t)$ is given by the inverse of the doubling time. A higher doubling rate reflects the faster spread of infection. This rate is often used to measure the effect of social distancing campaigns, improved hygiene, and case tracking.

The doubling time for $C_t$ computed using the relationship $C_{t+t_d(t)}/C_t=2$. A first order approximation (see Supplement Section S4.) yields $t_d(t) \approx \left[ \frac{d}{dt}\log C_t\right] ^{-1}$. That is the doubling rate ${\tilde{\xi }}(t)=t_d(t)^{-1}=\frac{d}{dt}\log C_t$. Doubling rates for other compartments can be computed similarly.

#### Crude and net case fatality rates

In general a *case fatality rate* at time *t* is given by the ratio of the total death count and the total case count at that time. Depending on whether the reported case counts or the actual case counts are used, we can define two different case fatality rates. The *crude case fatality rate* (CFR) is defined as:

13 $$\begin{aligned} CFR(t) = \frac{D_t}{C_t}\times 100, \end{aligned}$$

whereas the *net case fatality rate* is given by

14 $$\begin{aligned} NFR(t)=\frac{D_t}{CNI(t)}\times 100. \end{aligned}$$

#### Basic reproduction rate

In the conventional SIR or SEIR models, basic reproduction rate ($R_0$), which measures the expected number of cases directly generated by one case in a population where all individuals are susceptible to infection^55^, is used to determine the nature, rate of growth and possible measures for controlling the pandemic^27,28^.

Our model is more detailed and allows for time varying parameters and as a result, the conventional $R_0$ cannot be directly estimated from our model. The closest epidemiological quantity we can observe is the background infection rate, $\alpha$, measuring the average number of susceptible individuals who may be infected on any given day by an asymptomatic but infected individual. However, an analogue of the basic reproduction rate for the compartment $A_t$ can be computed^56,57^.

By focusing on the compartment $A_t$, under our assumptions from Eq. (2) new infections arrive at the compartment at the rate of $\alpha \kappa _t^2S_t/(S_t+A_t+R_t)$ and leave at the rate of $(\theta (t)+\gamma +\rho _A)$. There is no other pathway for disease spread. Thus we can define an analogue of the basic reproduction rate as:

15 $$\begin{aligned} {\tilde{R}}_0(t)=\frac{\alpha \kappa _t^2}{\theta (t)+\gamma +\rho _A}\left( \frac{S_t}{S_t+A_t+R_t}\right) . \end{aligned}$$

Note that, the proposed ${\tilde{R}}_0(t)$ can be interpreted in the same way as the conventional basic reproduction rate. By construction, ${\tilde{R}}_0(t)<1$ indicates negative growth of the number of asymptomatic-infected persons, whereas ${\tilde{R}}_0(t)>1$ indicates its positive growth. However, temporal variation of ${\tilde{R}}_0(t)$ is more complex. Assuming that, $S_t/(S_t+A_t+R_t)\approx 1$, ${\tilde{R}}_0$ can decrease with time either due to reduction in $\kappa _t$, that is the current state of interaction among individuals, or due to an increase in the confirmed fraction $\theta (t)$. That is, the proposed ${\tilde{R}}_0(t)$ is directly influenced by the mitigation efforts such as social distancing, adherence to the use of masks, increased testing and subsequent quarantining, hospitalization of symptomatic patients, etc.

Most epidemiological models such as SIR, SEIR, etc., assume fixed doubling rate parameters. In reality, however, the doubling time is a dynamic quantity, which changes continuously due to mitigation efforts and the inherently changing nature of virus-spreading mechanisms. It is then vital that policymakers and researchers have access to frequent and up-to-date estimates of doubling time^58^. For example, fixed-in-time estimates of epidemic parameters of COVID-19 (e.g. growth rate, doubling time, basic reproduction number, case detection rate) during the first 50 days of onset in China is provided^59^. In recent work^60^ the basic reproduction number and doubling time have been studied in a dynamic manner by considering a varying coefficient model with daily new cases as the response and time as a predictor. A related approach focused on the real-time estimation of case fatality rates using Poisson mixture models can be found in^61^.

## Methods : parameter and compartment estimation

The core of our estimation strategy is to utilize Eqs. (1)–(7) to formulate appropriate regression problems. Our estimation procedure is based on the availability of the compartments $C_t$, $D_t$, $H_t$, $Q_t$, $T_t$ and $R^{reported}_t$ only. We do not assume that data on the social distancing factor $\kappa _t$ is available. Described crudely, the proposed estimation method uses local regression (linear or nonlinear) methods for estimating the time-varying parameters, while *profiling* over the time-independent ones.

In the absence of data on $\kappa _t$, the parameter $\alpha$ in Eq. (1) is not identifiable. We first describe how the product $\alpha \kappa ^2_t$ can be estimated. Notice that ignoring the stochastic nature, we may rewrite equation (5) as

16 $$\begin{aligned} \Delta C_t = (\theta (t)+\gamma ) A_t. \end{aligned}$$

Defining $\eta (t) = \theta (t)+\gamma$, and applying the difference operator on both sides of Eq. (16), and finally dividing both sides by $\Delta C_t$, we obtain

17 $$\begin{aligned} \frac{\Delta ^2 C_t}{\Delta C_t} = \left( 1 + \frac{\Delta \eta (t)}{\eta (t)}\right) \frac{\Delta A_t}{A_t} + \frac{\Delta \eta (t)}{\eta (t)}. \end{aligned}$$

Now, ignoring the second order factor $(\Delta \eta (t)\Delta A_t)/(\eta (t) A_t)$, from Eq. (2), at the onset of the epidemic (i.e. $S_t/(S_t+A_t+R_t)\approx 1$), we have the approximate relationship:

18 $$\begin{aligned} \frac{\Delta ^2 C_t}{\Delta C_t} \approx \frac{\Delta \eta (t)}{\eta (t)} - \eta (t) - \rho _A + \alpha \kappa _t^2. \end{aligned}$$

Note that Eq. (18) establishes an approximate linear relationship, between the observable quantity $\Delta ^2 C_t/\Delta C_t$ and the product $\alpha \kappa _t^2$. Below we show that, the other parameters in equation (18) can be estimated, from the available data. These estimates can be plugged in to get an estimate of $\alpha \kappa _t^2$.

### Point estimates

Broadly speaking, the estimation strategy consists of separating the time-dependent and time-independent parameters, into vectors $\varvec{\beta }_t = (\phi (t),\rho _H(t),\delta (t))$ and $\varvec{\zeta }= (\gamma ,\rho _A)$ respectively. First the vector $\varvec{\zeta }$ is kept fixed and for each *t* the time-dependent parameter $\varvec{\beta }_t$ is estimated (denoted $\widehat{\varvec{\beta }}^h_t(\varvec{\zeta })$) by minimizing the “conditional” local loss function $\widetilde{L}_t^h(\varvec{\beta }_t|\varvec{\zeta })$ (described below) with respect to $\varvec{\beta }_t$, subject to appropriate constraints on the parameters (non-negativity as well as certain upper bounds). The optimal local conditional loss is then combined across different time points to obtain the *profile loss* function for $\varvec{\zeta }$, which is given by

19 $$\begin{aligned} L^h(\varvec{\zeta }) = \sum _{t} \widetilde{L}_t^h(\widehat{\varvec{\beta }}^h_t(\varvec{\zeta })|\varvec{\zeta }). \end{aligned}$$

The estimate $\widehat{\varvec{\zeta }}^h$ of $\varvec{\zeta }$ is obtained by minimizing $L^h(\varvec{\zeta })$ under appropriate constraints. We update the estimates of $\varvec{\beta }_t$ as $\widehat{\varvec{\beta }}^h_t = (\widehat{\phi }(t),\widehat{\rho }_H(t),\widehat{\delta }(t))= \widehat{\varvec{\beta }}^h_t(\widehat{\varvec{\zeta }}^h)$.

In order to define the conditional loss function, let $K(\cdot )$ be a non-negative kernel integrating to one. Now, for a bandwidth parameter $h > 0$, the *local weighted conditional loss* function of $\varvec{\beta }_t$, given $\varvec{\zeta }$ is defined as:

20 $$\begin{aligned} \widetilde{L}_t^h(\varvec{\beta }_t|\varvec{\zeta }) = \sum _{s} \frac{1}{h}K\left( \frac{t-s}{h}\right) d_{s}(\varvec{\beta }_t|\varvec{\zeta }) \end{aligned}$$

where

21 $$\begin{aligned} d_{s}(\varvec{\beta }_t|\varvec{\zeta })= & {} \left| \sqrt{\Delta H_s + \Delta D_s + \Delta R^{reported}_s} - \sqrt{(\rho _A + \gamma ) Q_s + \frac{\gamma \Delta C_s}{\phi (t)F_s + \gamma }} \right| ^2 \nonumber \\&+ \left| \sqrt{\Delta R^{reported}_s} - \sqrt{\rho _A Q_s + \rho _H(t) H_s}\right| ^2 + \left| \sqrt{\Delta D_s} - \sqrt{\delta (t) H_s}\right| ^2. \end{aligned}$$

Note that the RHS of Eq. (21) only uses the observed data. The first addendum originates from equations (4), (5) and (6). The second and the third term use Eqs. (6) and (4) respectively. The square-root transformation of the responses are used as a variance stabilizing transformation, which is driven by the assumed Poissonian characteristics of the responses. Also by construction, the estimate of $\delta (t)$ does not depend on $\varvec{\zeta }$.

Estimated values of the parameters readily yields estimates of the key compartments of the model. In particular, from the definition of $\theta (t)$, Eqs. (16) and (17) we get:

$$\begin{aligned} {\widehat{\theta }}(t) = {\widehat{\phi }}(t)\frac{\Delta T_t}{H_t}, \quad {\hat{A}}_t = \frac{\Delta C_t}{{\widehat{\theta }}_t+{\hat{\gamma }}}, \quad \widehat{\left( \frac{\Delta A_t}{A_t}\right) }=\frac{\frac{\Delta ^2 C_t}{\Delta C_t}-\frac{\Delta {\hat{\theta }}(t)}{{\hat{\theta }}(t)+{\hat{\gamma }}}}{1+\frac{\Delta {\hat{\theta }}(t)}{{\hat{\theta }}(t)+{\hat{\gamma }}}}, \quad \widehat{\Delta A_t}=\widehat{\left( \frac{\Delta A_t}{A_t}\right) }{\hat{A}}_t. \end{aligned}$$

Now, by plugging in ${\hat{\gamma }}$, ${\widehat{\theta }}(t)$, ${\hat{A}}_t$ and ${\hat{\delta }}(t)$ in Eq. (4) we get an updated estimator of $\rho _H(t)$ as

$$\begin{aligned} {\widehat{\rho }}_H(t)=\frac{\Delta H_t-{\hat{\gamma }}({\hat{A}}_t+Q_t)+{\hat{\delta }}(t) H_t}{H_t}. \end{aligned}$$

Finally, using Eq. (17) an estimate of $\alpha \kappa ^2_t$ can be obtained as:

$$\begin{aligned} \widehat{\alpha \kappa ^2_t}=\widehat{\left( \frac{\Delta A_t}{A_t}\right) }+{\hat{\theta }}(t)+{\hat{\gamma }}+{\hat{\rho }}_A. \end{aligned}$$

The rest of the compartments can be estimated by plugging in the appropriate parameter or compartment estimates in equations (1)–(7) (see the Supplement Sections S1. and S3.).

The tuning parameter *h* in equation (20) is obtained by minimizing a standardized $L_1$ distance between the fitted and model based estimates of various compartments through a cross-validation strategy. The actual minimization is achieved by a grid search. Details can be found in the Supplement Sections S1. and S2.

### Confidence intervals

We employ residual bootstrap^62–64^ to compute the confidence intervals for our parameter and compartment estimates. Briefly put, the technique adds resampled residuals to the fitted values to create several “resampled” datasets. The point estimation technique described above is applied to each of these resampled datasets to create a new set of parameter and compartment estimates. The empirical distribution of these estimates is then used to construct the confidence interval. The details of the algorithm can be found in Supplement Section S5. The theoretical validity of the residual bootstrap method is well justified in existing literature^65,66^.

## Results : application to COVID-19 data from the US

### Data preparation

We consider the dynamics of the spread of COVID-19 in various states of the US for a tentative time window of late April to mid-December, 2020. The proposed model is based on the observed state-wise daily counts of confirmed infections, deaths, hospitalizations, and reported recoveries from the hospitals and quarantine facilities. Daily counts of the confirmed COVID-19 cases in various states were obtained from the COVID-19 Data Repository maintained by the Center for Systems Science and Engineering (CSSE) at Johns Hopkins University. This is publicly available at https://github.com/CSSEGISandData/COVID-19 and was accessed on December 15, 2020. The state-wise daily counts of positive and negative COVID-19 test results, current hospitalization, and recovery per day and state were obtained from the CDC data repository - the COVID Tracking Project and are publicly available at https://COVIDtracking.com/ (accessed on December 15, 2020.)

The collected noisy data used is pre-processed and cleaned, removing the irregularities present in the recording and maintenance of the data repositories. Any missing or evidently wrong (e.g. negative counts) observations were replaced by the average of the data from the adjacent five days. Inherent noise present in the daily counts was removed by pre-smoothing the trajectories using a *Lowess* method^67–69^ with bandwidth 1/16.

### Results

Unfortunately, continuous records on hospitalization and recovery information were not available for many states. For example, most counties in California are not reporting recovery information. Data on hospitalization is found to be updated once a week in Massachusetts and Florida. New York, on the other hand, started documenting the hospitalization information only after the initial surge of the pandemic was over for the state. In our analysis we only consider the states for which daily observations on $C_t,$ $D_t,$ $R^{reported}_t,$ $Q_t,$ and $H_t$ are available throughout the time window under consideration. Any missing/negative values are replaced by the average of the adjacent five days’ data. For a few states e.g. Alabama, the available data turned out to be too unreliable. We present results for fifteen states in the US that demonstrate the efficacy of the proposed model and the estimation methods. For succinct representation, the results from only one state i.e. Utah are presented in detail below. The results for the other fourteen states can be found in the Supplement Section S8.

#### Case study for the state Utah

**Figure 2:**
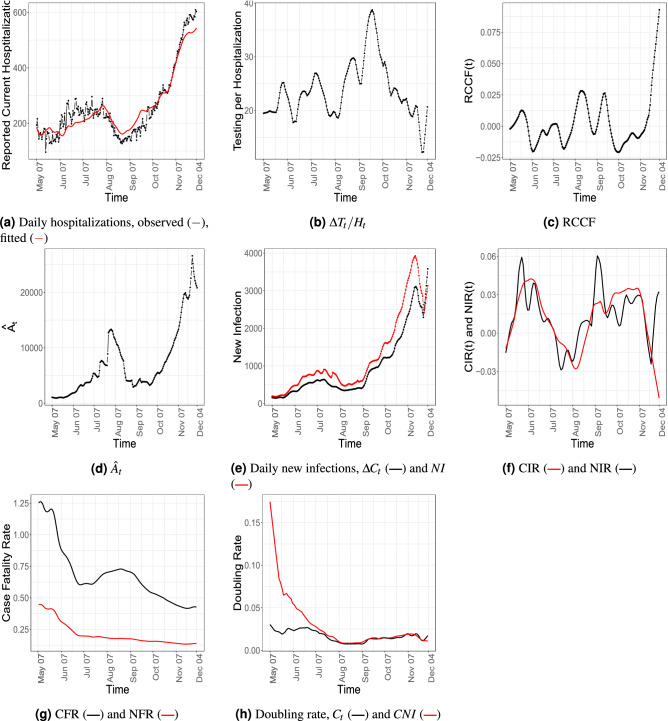
Temporal patterns of some compartments and epidemiological markers for Utah.

**Figure 3:**
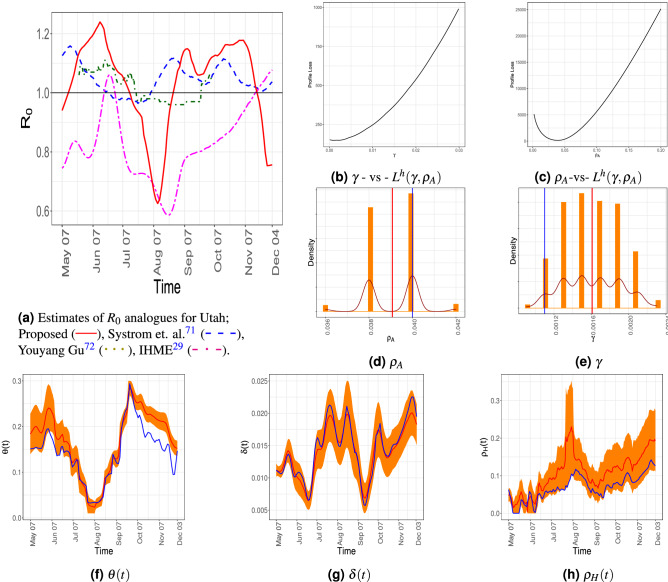
(**a**) Estimates of $R_0$ analogues. (**b**–**c**) Estimates and residual bootstrap based confidence intervals for time invariant and time-varying parameters for the state of Utah. The estimate from the data is in blue. The $95\%$ confidence band is in yellow and the mean of the bootstrap estimates are presented in red.

We present our results for the state of Utah for the time window between 7th May, 2020 to 4th December, 2020. The time interval includes the Thanksgiving weekend (27th -28th November, 2020), when due to the long holiday, the reported data may be unreliable. In Fig. 2 plots of various time-varying compartments and epidemiological markers defined in “Some relevant epidemiological markers” section. The plots of the parameters with their residual bootstrap confidence intervals can be found in Fig. 3. Due to unreliable reporting around the Thanksgiving holiday, the estimated values after 21st November, 2020 should be interpreted with caution.

The curves in Fig. 2a compare the observed and the fitted number of daily number of people in the hospitals. It can be seen that the fitted values obtained from the model closely follow the observed values. This validates our proposed model and the estimation procedure. From the data and the fit two waves of infection can be identified. It seems the first wave starts at the end of May, 2020 stabilizes and begins to die down around 7th August, 2020. The daily number of people in hospitals starts increasing again around the end of August, 2020.

#### Estimation of latent compartment

The estimated number of infected asymptomatic people (Fig. 2d) shows a similar pattern. From a high point around the beginning of August, it dips to a low value at the end of August. The number remains stable for a few weeks and starts growing again at the end of September. Estimation of such latent trajectory is a key feature of our proposed methodology which cannot naturally be obtained from the conventional epi-models. The projections from IHME^29^ which employ a more complex but less robust parametric estimation method based on an SEIR model provides an estimate of a “pre-symptomatic” population. Members of this compartment can be considered asymptomatic. We use the term in a more general sense.

#### Analogue of basic reproduction rate

The phenomenon of two waves is clearly observed from the plot of the proposed analogue of the basic reproduction rate ${\tilde{R}}_0$ (the solid red curve in Fig. 3a)—the estimated ${\tilde{R}}_0$ was larger than 1 in two sub-intervals, namely from middle May to middle of July and then from the end of August to the beginning of November. Our estimate is compared with three other relevant sources viz., the generative COVID-model considered by the Systrom et al.^70^ (blue, “longdash” line in Fig. 3a), the SEIR model based COVID-19 projections using machine learning from Youyang Gu^71^ (in green), and SEIR model used by the IHME team in^29^ (in magenta).

Our analogue of this epidemiological marker seems more realistic since it tallies with the other observed and estimated compartments. For example, around August 7, 2020, the cumulative new infections (CNI), both observed and estimated, hospitalization and the asymptomatic population (estimated) were quite low and almost constant over a period of time (see Supplement Section *S*7). The estimated social mobility index $\alpha \kappa _t^2$ also experienced a sharp decline around that time (see Figure 10 in Supplement Section *S*7), which all give evidence to the fact that the spread of the pandemic was indeed contained around mid-July to mid-August in Utah. This is clearly resonated in our version of reproduction rate but is not so well captured by the two other models considered above. The estimate released by IHME^29^ seems to follow our estimate in August, however, it hardly gets higher than 1, not even in October, when the number of new infections was high. From this, it seems that the IHME estimate does not qualitatively reflect the real nature of COVID spread.

The plot of the number of daily new and daily reported infections (Fig. 2e) shows a local maximum near the middle of November. However, we cannot rule out the boundary effect as its cause.

**Table 1:** Estimates, and the residual bootstrap Confidence intervals, mean and standard deviations for the time-invariant parameters for Utah. The latter three are computed based on 1000 bootstrap resamples.

	Estimate	$95\%$ confidence interval	Mean	SD
$\gamma$	0.0011	$[0.0011, \ 0.0021]$	0.0016	0.0003
$\rho _A$	0.0400	$[ 0.0360, \ 0.0420]$	0.0391	0.0012

#### Model parameter estimates

The estimate of $\delta (t)$ in Fig. 3g seems to remain stable throughout the time period under consideration. The $\widehat{\rho _H}(t)$ shows an overall increasing trend. On the other hand, the estimate of $\theta (t)$ decreases to a near-zero value at the end of the first wave (7th August, 2020) it then increases to its maximum value at the end of September and starts to decrease again. The parameters $(\gamma ,\rho _A)$ are estimated based on minimization of the profile loss using a grid search algorithm with grid size 0.0001. In Fig. 3d,e the estimates from residual bootstrap samples take discrete values, resulting in a discrete histogram counts. In Table 1 we present the estimates, 95% residual bootstrap confidence intervals, the residual bootstrap mean and standard deviations of the above parameters.

#### Transmission rates

The plots of CIR and NIR seem to be similar (Fig. 2f). In fact, the observed doubling rate obtained from $C_t$ and that estimated from *CNI* seems to be very close in the second wave of the pandemic (see Fig. 2h). This implies that in the second wave the reporting kept pace with the spread of the disease. Figure 2g shows the crude and net fatality rates. Due to the denominator effect, naturally, the crude fatality rate is much larger than the net fatality rate. However, our estimate of NFR is mostly below $0.25\%$, which complies with widely held beliefs^29,72–74^.

**Table 2:** Table comparing the seroprevalence estimates for the state Utah.

	CNI/Population	Recovery/Population	Average Seroprevalence
Period 1	1.78	1.62	3.2
(July 27–August 13)	1.144, 3.597)	(1.595, 2.152)	(1.20, 5.03)
Period 2	2.00	1.93	5.5
(August 10–August 27)	(1.258, 4.090)	(1.922, 2.493)	(2.94, 8.71)
Period 3	2.27	2.17	4.9
(August 24–September 10)	(1.420, 4.500)	(2.163, 2.733)	(2.82, 7.67)
Period 4	2.41	2.44	5.1
(September 7–September 24)	(1.748, 5.105)	(2.440, 3.020)	(3.29, 7.90)

#### Seroprevalence

Seroprevalence studies to estimate the prevalence of persons with SARS-CoV-2 antibodies have been of immense interest. Seroprevalence is calculated as the number of reactive specimens divided by the number of specimens tested^75^. Even though our model cannot explicitly compute it, analogues of such estimates can be found from the ratios such as percentage of cumulative new infections in the population and the percentage of total recovery (from quarantine, hospitalization, or asymptomatic states). The estimates of such seroprevalence analogue for the state of Utah are illustrated in Table 2 and the $95\%$ residual bootstrap confidence intervals mostly overlap with the $95\%$ confidence intervals provided in^75^ for all four periods of time considered.

#### Testing and hospitalization

The daily number of tests and its effect in quarantining asymptomatic but infected people can be judged from the Fig. 2b,c. The state of Utah increased its testing capacity by a public-private partnership. An empirical comparison of the Fig. 2a,b seems to reveal that although the number of daily tests could keep pace with the daily number of hospitalized patients up to the third week of September, the growing number of hospitalized people ultimately outpaced the number of daily tests. Note that estimated $\theta (t)$ increases at the onset of the second wave (see Fig. 3f between 7th, August and 21st, September), however, from Fig. 2d, ${\hat{A}}_t$ remains more or less constant. Thus, growth in the number of new infections could be due to the increase in $\kappa _t$, which is due to more interaction among individuals and less social distancing.

**Figure 4:**
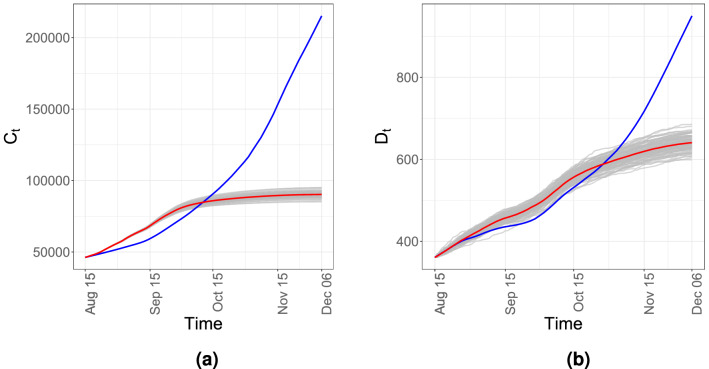
The 100 simulated trajectories when testing rate is increased by 30% are in gray while the mean of them are presented in red. The observed $C_t$ and $D_t$ in Utah are marked in blue.

#### Impact of testing in the disease control

From a public policy perspective, our model-based simulation provides strong quantitative evidence on the significant role of testing rate in controlling the spread of the pandemic. This could be the key to mitigating the explosive nature of the epidemic even before any intervention strategies are put into practice. Numerical simulation of the pandemic based on the estimates obtained from our model shows explicitly that, with all the time-invariant and time-varying rate parameters remaining the same, a higher testing rate leads to suppression and eventual decline in the number of infected individuals as well as hospitalizations and deaths (see Supplement Section *S*7). For example, Fig. 4 shows that the curves are clearly flattened when the confirmed fraction, $\theta (t)$ is increased by $30\%$. Non-increasing patterns shown in the cumulative compartments, $C_t$, and $D_t$ indicate a containment of the disease.

#### Summary of results for other states

We present a summary of the results obtained from applications of the proposed method on the data procured from fifteen other states in the US. The estimated parameters are in Table 3. The time-varying parameters, ($\phi (t)$, $\rho _H(t)$, $\delta (t)$), are summarized by their means. The computed ${\hat{\gamma }}$, that is, the rate for an asymptomatic person turning symptomatic on a particular day is the smallest in Arizona and largest in Tennessee. This estimate is smaller than 0.001 for Arizona and Idaho. Minnesota has by far the highest recovery rate for an asymptomatic person without needing hospitalization on a particular day (i.e. $\widehat{\rho }_A$). For Iowa, Nebraska, Pennsylvania, and Utah this rate is comparable and reasonably high, whereas Arizona, Delaware, and Idaho have their $\widehat{\rho }_A$ value below 0.01. The average confirmed fraction $\overline{{\hat{\theta }}}$ is larger than 0.1 in Delaware, Tennessee, and Utah. It is the lowest in Texas. This can be associated with better estimates obtained for these states due to the availability of more reliable data, whereas for Idaho, South Dakota, and Texas, a lower value of their epi-markers tends to give evidence for a more relaxed testing paradigm. More testing is required for isolating the confirmed cases to contain the disease faster, which can be reflected in the numbers for these states. The detailed results and bootstrap confidence regions for these additional states can be found in Supplement Section S8.

Among the states not included in Table 3, many, such as California did not report all the required compartments. For many states such as Alabama, Colorado, Maryland, Massachusetts, North Carolina etc. the reported data produced monotone profile likelihoods which yielded unreliable boundary estimates. This could be due to the change in the definition of many compartments over time, which violated our assumptions. Furthermore, for some states such as New York, New Jersey, Michigan etc., the pandemic started quite early and ran its course even before a proper testing protocol and other mitigation measures could be introduced. Thus the data from these states is contaminated with an inherent bias, the number of people in quarantine or symptomatic states is too low to produce reliable estimates.

**Table 3:** Mean estimated parameters for different states in the US.

	${\hat{\gamma }}$	${\hat{\rho }}_A$	$\overline{\widehat{\delta (t)}}$	$\overline{\widehat{\rho _H(t)}}$	$\overline{\widehat{\theta (t)}}$	$\overline{\widehat{\phi (t)}}$
Arizona	0.0003	0.002	0.0208	0.0023	0.0887	0.0079
Arkansas	0.0029	0.094	0.0249	0.0975	0.0809	0.0038
Delaware	0.0017	0.008	0.0159	0.0093	0.1076	0.0037
Idaho	0.0009	0.010	0.0230	0.0138	0.0289	0.0019
Iowa	0.0011	0.032	0.0263	0.0372	0.0478	0.0033
Minnesota	0.0023	0.128	0.0315	0.0654	0.0899	0.0034
Nebraska	0.0011	0.020	0.0141	0.0266	0.0394	0.0035
Ohio	0.0023	0.048	0.0180	0.0532	0.0625	0.0024
Oklahoma	0.0037	0.084	0.0122	0.1029	0.0494	0.0033
Pennsylvania	0.0013	0.026	0.0293	0.0372	0.0535	0.0033
South Dakota	0.0021	0.058	0.0190	0.0922	0.0262	0.0038
Tennessee	0.0059	0.064	0.0158	0.0413	0.1206	0.0076
Texas	0.0019	0.036	0.0207	0.0341	0.0212	0.0013
Utah	0.0011	0.040	0.0144	0.0252	0.1434	0.0061
Wisconsin	0.0017	0.068	0.0217	0.0707	0.0477	0.0026

## Discussion

We introduce a multi-compartment model for COVID-19 dynamics which can incorporate data from compartments like quarantine, hospitalization, etc. Unlike the conventional SIR and similar models, the proposed model is based on interpretable time-varying parameters, which are more suitable for describing the disease dynamics in the presence of mitigating procedures. It also incorporates information about testing and subsequent quarantining. We estimate the model parameters using profile likelihood and nonparametric regression. This provides a much faster alternative to Markov Chain Monte Carlo-based Bayesian models which are commonly used in estimating SIR parameters. Using the proposed detailed and robust model one can estimate the daily number of asymptomatic but infected individuals, who are universally regarded as the key agent for the COVID-19 spread. To the best of our knowledge, no other model gives both such epi-estimates, which are important from a health policy perspective, as well as the projections for the un-observable latent quantities such as the trajectories of susceptible, asymptomatic, and recovered (from quarantine, hospitalization, or asymptomatic states) population, which are essential for understanding the dynamics of the pandemic. We define several epidemiological markers that use the number of asymptomatic-infected individuals and therefore reveal the true underlying dynamics of the pandemic.

Our model only uses information on the number of confirmed infected, hospitalized, deaths, and total reported recoveries from hospitals and quarantine. We don’t require those numbers separately. However, such numbers are often available. In such a case, the loss function in Eq. (21) can be simplified a bit. The details can be found in the Supplement in Section S2.

In this article, the model parameters have been estimated without assuming that any information about the mobility within the population, or degree of restrictions on the interaction among people are available. Therefore, apart from the parameters $\gamma$, $\rho _A$, $\theta (t)$, $\rho _H(t)$ and $\delta (t)$, we can estimate the function $\alpha \kappa _t^2$, but not the social distancing index $\kappa _t$ and baseline infection rate $\alpha$ separately. Additional information on mobility or social distancing restrictions would enable the determination of the parameters $\kappa _t$ and $\alpha$ in our model. Specifically, if accurate information on $\kappa _t$ is available, the parameter $\alpha$, which is the average number of susceptible individuals who may be infected in a day by an asymptomatic-infected individual, is identifiable and can be estimated. The details of the estimator can be found in Section S5 of the Supplement. Reliable data on the compliance to social distancing, mask wearing etc. is difficult to get. Various aspects of the mobility data available from e.g. Google can be one potential surrogate for $\kappa _t$^76,77^. However, such data only reflect the fraction of people going to their workplace or places of recreation, and so on, and such sources do not collect information on individuals who are super spreaders or not wearing masks, etc. Thus, the collected data as such does not necessarily reflect the social distancing index $\kappa _t$, as interpreted in our model. In the Supplement (see Section S8.), we estimate $\alpha$ by using, as a surrogate to $\kappa _t$, the publicly available Google mobility data sourced from https://www.google.com/covid19/mobility/. If one is interested in separately estimating this parameter, mobility data from many similar sources such as SafeGraph, Apple, Facebook, etc. may be alternatively used. However, the parameter $\alpha$ was not of primary interest to us, neither its estimation was necessary for our proposed procedure.

The proposed method and estimation procedure do not explicitly use the underlying assumption of a Poisson process. In the Supplement (see Section S$6.-$ 7.), however, we use an ensemble of independent Poisson processes to simulate data from the proposed model. These aggregated data sets are then used to accurately estimate various parameters, which validate our estimation procedure. The aggregation has the effect of increasing the number of observations in the compartments and thereby improving estimation accuracy. If the number of individuals in the symptomatic or quarantined compartments is low, e.g. at the onset of the pandemic, inherent biases are introduced in the estimated trajectories. A bigger sample size is required to correct such contaminants.

In our model, the compartment $A_t$ includes the asymptomatic individuals, as well as those infected before they are quarantined, tested positive, or hospitalized. We further assume that anybody, whether quarantined or not, is immediately hospitalized, and is tested positive, upon the onset of significant symptoms. In reality, however, some symptomatic people might not get tested and remain in the community as spreaders. Furthermore, the rate at which a truly asymptomatic person infects a susceptible may differ from the same rate for a non-tested mildly symptomatic person. In practice, little data is available on mildly symptomatic people. Under the ideal situation we consider here, such differences should be negligible.

Because of the limited availability and relatively poor quality of detailed data, we allow no strata with respect to age or intrinsic vulnerability to the disease in our homogeneous population. Moreover, due to the presence of unobservable compartments like $A_t$, even when the data quality is good, there is a near lack of identifiability of the parameters if all of them are assumed to be time-dependent. In any case, for most practical situations, it is reasonable to assume a constant rate of recovery $\rho _A$ and a constant rate $\gamma$ of getting severely ill from the asymptomatic compartment. We consider dynamic models of pandemic propagation in a stratified population in a subsequent article.

Since the proposed method is non-parametric, they suffer from possible boundary effects near the end-points of the time window. It should also be noted that COVID-19 analyses based on the published case and death counts, including those conducted here, are subject to the same biases which affect the accuracy of the data, primarily due to under-reporting^78^ or misrecording of the data, the degree of which varies by country^79^. The reasons for such under-reporting are many, including insufficient testing materials, political incentives, and administrative delays. If such irregularities are present even after pre-processing steps, the underlying model in  (1)–(7) may not be adequate. In such cases, the profile loss functions of $\gamma$ and $\rho _A$ in (19) may attain their minimums at the boundaries. This may influence other parameter estimates and their interpretations. Furthermore, our model assumes a closed population. It ignores migration between cities, states, or countries which play an essential role in the propagation of the disease. We only count the deaths solely due to COVID-19 infections and as such completely ignore any competing causes of morbidity, as well as increase in population due to new births.

With this caveat in mind, the study of available data presented in this article nevertheless provides useful insights into the COVID-19 propagation and ways to control it. It clearly follows that in order to break the chain of transmission and “flatten the curve”, we need extensive testing and adhere to strict social distancing protocols.

## Supplementary Information

Supplementary Information.

## Data Availability

All data necessary for the replication of our results is collated in https://github.com/Satarupa3671/COVID-19-Nonparametric-Inference. The data for the number of COVID cases, deaths, hospitalizations and recovery were originally collected from https://covidtracking.com/data/download while the social mobility data was sourced from https://www.google.com/covid19/mobility. All code necessary for the replication of our results is collated in https://github.com/Satarupa3671/COVID-19-Nonparametric-Inference.
