# Supplementary material for: Inference on the dynamics of COVID-19 in the United States
Source: Sci Rep. 2022 Feb 10;12:2253. doi: 10.1038/s41598-021-04494-z (PMC8831615; doi:10.1038/s41598-021-04494-z)
Supplement: Supplementary file 1 — Supplementary Information. [file 41598_2021_4494_MOESM1_ESM.pdf]

Supplement to Inference on the dynamics of COVID-19 in the United States

Satarupa Bhattacharjee[a], Shuting Liao[b],

Debashis Paul[a], Sanjay Chaudhuri[c*]

[a] *Department of Statistics, University of California, Davis*

[b] *Graduate Group in BioStatistics, University of California, Davis*

[c] *Department of Statistics and Applied Probability, National University of Singapore*

S1. Choice of bandwidth h

The optimization of the profile loss function and hence the parameter estimation depends on the choice of the tuning parameter h. We choose the optimal h, that minimizes the standardized L_1 distance between the fitted and model-based estimates for (i) the new number of confirmed cases at time t, (ii) the number of new infections at time t, and (iii) the number of cumulative infections at time t.

It should be noted that the number of new confirmed cases at time t (denoted C_t) is an observable quantity and often under-reported due to the prevalence of the asymptomatic population. The comparable model-based new infection is approximately given by $\widehat{\alpha\kappa_t^2}\widehat{A}_t$. This model-based estimate depends on the choice of h. The difference between the above two quantities measures the number of unreported new cases.

The number of new asymptomatic infections can be calculated in two ways- the model-based growth in A_t given by $\widehat{\Delta A_t} = NIR(t)A_t$ (see Section 2.2.2 for the definition of $NIR(t)$), and the difference of the estimated A_t fitted from the data given by $\Delta\widehat{A_t}$. A

E-mail address: * Corresponding author: stasc@nus.edu.sg.

higher value of the discrepancy between the two quantities is likely to indicate a model mismatch. To this end, the cumulative version of the above difference is also considered.

The three different objective functions contribute to the efficacy of the model in the context of the observed data. Combining these in an additive manner, we then proceed to find the minimizer h. As such,

$$h^* = \underset{h}{\text{argmin}} \sum_t \left(\frac{|\widehat{\alpha\kappa_t^2}(h)\widehat{A_t}(h) - \Delta C_t(h)|}{|\widehat{\alpha\kappa_t^2}(h)\widehat{A_t}(h)| + |\Delta C_t(h)|} + \frac{|\widehat{\Delta A_t}(h) - \Delta\widehat{A_t}(h)|}{|\widehat{\Delta A_t}(h)| + |\Delta\widehat{A_t}(h)|} \right.$$
$$\left. + \frac{|\sum_{s=1}^t \widehat{\alpha\kappa_s^2}(h)\widehat{A_s}(h) - C_t(h)|}{|\sum_{s=1}^t \widehat{\alpha\kappa_s^2}(h)\widehat{A_s}(h)| + |\Delta C_t(h)|} \right) \tag{S.1}$$

S2. A different scenario of reported recovery

The estimation procedure may differ in case R_t^H and R_t^Q were reported separately. In such case, we give a different version of the loss $d_s(\boldsymbol{\beta}(t)|\boldsymbol{\zeta})$ below, corresponding to this alternate reporting regime.

$$d_s(\boldsymbol{\beta}(t)|\boldsymbol{\zeta}) = \left| \left(\Delta H_s + \Delta D_s + \Delta R_s^H\right)^{1/2} - \left(\gamma Q_s + \frac{\gamma \Delta C_s}{\phi(t) F_s + \gamma}\right)^{1/2} \right|^2$$
$$+ \left| \sqrt{\Delta R_s^Q} - \sqrt{\rho_H(t)}\sqrt{H_s} \right|^2 + \left| \sqrt{\Delta D_s} - \sqrt{\delta(t)}\sqrt{H_s} \right|^2. \tag{S.2}$$

In either equation (21) (see Section 3.1) or (S.2), the use of square-root transformation of the response is driven by the Poissonian character of the responses, and is done to stabilize the variance of the noise.

The estimation strategy consists of first fixing $\boldsymbol{\zeta} = (\gamma, \rho_A)$ and estimating $\boldsymbol{\beta}(t) = (\phi(t), \rho_H(t), \delta(t))$ for each t by minimizing the "conditional" loss function $\widetilde{L}_t^h(\boldsymbol{\beta}(t)|\boldsymbol{\zeta})$ with respect to $\boldsymbol{\beta}(t)$, subject to appropriate constraints on the parameters (non-negativity as well as certain upper bounds). Let us denote the resulting estimate by $\widehat{\boldsymbol{\beta}}_h(t|\boldsymbol{\zeta})$. Notice

however that the estimate of $\delta(t)$ does not depend on $\boldsymbol{\zeta}$, while if we use the description (S.2) for $d_s(\boldsymbol{\beta}(t)|\boldsymbol{\zeta})$, then the estimate of $\rho_H(t)$ also does not depend on $\boldsymbol{\zeta}$. After this, we combine the minimum value of the local loss across different time points to obtain the *profile loss* function for $\boldsymbol{\zeta}$ given by

$$L^h(\boldsymbol{\zeta}) = \sum_t \widetilde{L}_t^h(\widehat{\boldsymbol{\beta}}_h(t|\boldsymbol{\zeta})|\boldsymbol{\zeta}). \tag{S.3}$$

We obtain estimates $\widehat{\boldsymbol{\zeta}}_h$ by minimizing $L^h(\boldsymbol{\zeta})$ under appropriate constraints on $\boldsymbol{\zeta}$. Finally, the final estimates of $\boldsymbol{\beta}(t) = (\phi(t), \rho_H(t), \delta(t))$ are obtained as

$$\widehat{\boldsymbol{\beta}}_h(t) = (\widehat{\phi(t)}, \widehat{\rho_H(t)}, \widehat{\delta(t)}) = \widehat{\boldsymbol{\beta}}_h(t|\widehat{\boldsymbol{\zeta}}_h).$$

S3. Doubling times and rates

With C_t denoting the total confirmed cases per million up to time t, the *doubling time* $t_d(t)$ at time t is the length of time necessary for the cumulative cases to double. In other words, $t_d(t)$ is defined explicitly by the relation

$$\frac{C_{(t+t_d(t))}}{C_t} = 2. \tag{S.4}$$

By a first-order Taylor series expansion, a linear approximation of the numerator in (S.4) is

$$C_{(t+t_d(t))} = C_t + t_d(t)\frac{d}{dt}C_t. \tag{S.5}$$

(S.4) and (S.5) together implies

$$t_d(t) = \frac{C_t}{\frac{d}{dt}C_t} = \frac{1}{\frac{d}{dt}\log(C_t)}$$

The *doubling rate* $\tilde{\xi}(t)$ is defined as

$$\tilde{\xi}(t) = \frac{1}{t_d(t)} = \frac{d}{dt}\log(C_t). \tag{S.6}$$

The doubling rate quantifies the rate of spread, with lower doubling rates corresponding to longer doubling times and signify a better containment of the disease.

S4. Residual bootstrap procedure for inference

We employ a residual bootstrap based method to compute the confidence intervals for the estimated epidemiological parameters as well as the estimated compartments at each time point. To ensure positivity of the trajectories and to control variability, we first apply a square-root transform to the trajectories of the observed compartments C_t, D_t, H_t, Q_t and $R_t^{reported} = R_t^H + R_t^Q$. Then we obtain the residuals by taking daily differences of the difference between observed and fitted values (after square-root transform).

Since the errors in the data are dependent and possibly heteroskedastic, the residuals are adjusted to have zero mean, equal variance and low serial correlation before resampling. This is achieved by first subtracting the mean from each residual, then dividing the result by a windowed estimate of the local standard deviation. This procedure corrects for bias and heteroskedasticity. In order to get rid of the serial correlation, an $AR(1)$ process is fit to these standardized residuals. Finally, the estimated innovations from this $AR(1)$ fit are resampled with replacement (jointly across compartments). The resampled innovations are then used to create the resampled trajectories by reversing the process described above. A step-wise description of the algorithm is given in S4. The parameters and the compartments are then fitted based on many replications of the resampled data set. The confidence intervals are constructed from the estimates obtained from these data sets constructed from the resampled residuals.

Algorithm 1 Residual bootstrap implementation

Input: $(C_t, R_t^{reported}, Q_t, H_t, D_t)$ and $(\hat{C}_t,\ \hat{R}_t^{reported},\ \hat{Q}_t,\ \hat{H}_t,\ \hat{D}_t)$

Output: 95% confidence intervals for $\boldsymbol{\beta}(t)$, $\boldsymbol{\zeta}$ and α.

1: Compute the residuals corresponding to daily differences (after square root transform applied to the compartment) as: for compartment $X \in \{C, D, R^{reported}, H, Q\}$, compute

$$e_t^X = \Delta\sqrt{X_t} - \Delta\sqrt{\hat{X}_t}.$$

2: Obtain the mean-adjusted residuals, compute the windowed standard deviation $(s_{e^X}(t))$ of the residuals, and finally obtain the standardized residuals as

$$\tilde{e}_t^X = (e_t^X - \overline{e^X})/s_{e^X}(t).$$

where $\overline{e^X}$ is the mean of $\{e_t^X\}$.

3: Fit an AR(1) model to the process $\tilde{e}_{X_t}$, and denote the fitted values by $\widehat{\tilde{e}_t^X}$.

4: Obtain the estimated innovations (denoted r_t^X) from the AR(1) fit.

$$r_t^X = \tilde{e}_t^X - \widehat{\tilde{e}_t^X}.$$

5: Resample from the innovation process r_t^X jointly (for all $X \in \{C, R^{reported}, D, H, Q\}$, simultaneously) with replacement, to construct $r_t^{X,*}$. Obtain bootstrapped residuals

$$e_t^{X,*} = \overline{e^X} + s_{e^X}(t) \cdot (\widehat{\tilde{e}_t^X} + r_t^{X,*}).$$

6: Construct bootstrap trajectories for the daily differences

$$(\Delta\sqrt{X_t})^* = \Delta\sqrt{\hat{X}_t} + e_t^{X,*}.$$

7: Construct bootstrap trajectories (in the original scale) as

$$X_t^* = \left(\sqrt{X_1} + \sum_{t=1}^{T}(\Delta\sqrt{X_t})^*\right)^2.$$

8: Fit the model in Section 2.1, with X_t^* as data for all $X \in \{C, R^{reported}, D, H, Q\}$. For the b-th bootstrap sample obtain the bootstrap estimates $\widehat{\boldsymbol{\beta}}^{*b}(t)$, $\widehat{\boldsymbol{\zeta}}^{*b}$ and $\widehat{\alpha}^{*b}$.

9: Repeat *Steps 5 to 8* B times. Use percentile bootstrap procedure to find the confidence intervals with level 0.95 (pointwise C.I. for the time-dependent parameters respectively) based on the B bootstrap estimate.

S5. Estimation when κ_t is observed

We can adapt an iterative estimation method incorporating the information available on the social mobility parameter κ_t. The algorithm can be summarized as follows.

- Plug in the values of $\widehat{\eta}(t)$ in (18) (see Section 3) to create a new response $(\Delta^2 C_t/\Delta C_t) - \Delta\widehat{\eta}(t)/\widehat{\eta}(t) + \widehat{\eta}(t)$ and regress linearly onto κ_t^2.
- The slope gives a (crude) estimate of α. Negative of the intercept gives a new estimate of ρ_A. Plug in the new estimate $\widehat{\rho}_A(1)$ in the profile loss and minimize $L^h(\gamma, \widehat{\rho}_A(1))$ to obtain new estimates $(\widehat{\gamma}^{(2)}, \widehat{\boldsymbol{\beta}}^{(2)}(t))$.
- Iterate until convergence of the parameters.

We can refine the estimation strategy further by replacing the predictor κ_t^2 by $\left(\frac{\widehat{S}_t}{\widehat{S}_t+\widehat{A}_t+\widehat{R}_t}\right)\kappa_t^2$ in the regression step, where $\widehat{S}_t$, $\widehat{A}_t$ and $\widehat{R}_t$ are the (model-based) fitted values of the respective states based on the current estimates of the parameters.

$$\text{(S.7)} \qquad \widehat{\theta(t)} = \widehat{\phi(t)}\frac{\Delta T_t}{H_t}; \quad \hat{A}_t = \frac{\Delta C_t}{\widehat{\theta(t)} + \hat{\gamma}}; \quad \widehat{\Delta R_t^H} = \widehat{\rho_H(t)} H_t; \quad \widehat{\Delta R_t^Q} = \hat{\rho}_A\ Q_t;$$

$$\text{(S.8)} \qquad \widehat{\Delta R_t^A} = \hat{\rho}_A \widehat{A_t}; \quad \widehat{\Delta R_t} = \widehat{\Delta R_t^H} + \widehat{\Delta R_t^Q} + \widehat{\Delta R_t^A}; \quad \widehat{\Delta S_t} = \widehat{\alpha\kappa_t^2}\ \frac{\hat{S}_t}{(\hat{S}_t + \hat{R}_t + \widehat{A_t})},$$

where $\widehat{S}_t$, $\widehat{A}_t$ and $\widehat{R}_t$ are the iteratively fitted values of the respective states based on the current estimates of the parameters.

The baseline infection rate in the absence of any social distancing, given by α, can also be explicitly estimated in this case using information on the observable mobility data $\kappa(t)$ as follows

$$\text{(S.9)} \qquad \hat{\alpha} = \frac{\sum_{t=1}^{T} \widehat{\alpha\kappa_t}}{\sum_{t=1}^{T} \widehat{\kappa_t}}.$$

When the observable mobility data is reliable and works as an adequate representative of the social distancing measure, as described in our model, this direct estimate of α is likely to be close to the crude slope estimate of the regression problem described earlier.

S6. Aggregation simulation study based on the Poisson model

In this section, we present the results of a simulation study in order to partially justify the proposed estimation procedure. We also study the confidence intervals obtained from parametric as well as residual bootstrap procedures. Since the parametric bootstrap procedure depends on the model assumptions which may not be satisfied by the observed data, we use a more flexible residual bootstrap in our data analysis. This simulation exercise intends to compare the two procedures when the underlying true model is known.

S6.1. **Data generation and aggregation.** Given the values of the parameters, the simulated trajectories are modeled as linear combinations of sub-Poisson processes. To this end, we look at the individual drivers or branches that generate the dynamics at a granular level (see Figure 1 in Section 2)

$$\theta(t) = \phi(t)\frac{\Delta T_t}{H_t}, \tag{S.10}$$

$$P_{tA} \sim Poisson(\theta(t)A_t), \quad P_{gA} \sim Poisson(\gamma A_t), \quad P_{rA} \sim Poisson(\rho_A A_t), \tag{S.11}$$

$$P_{aA} \sim Poisson\left(\frac{S_t}{S_t + R_t + A_t}\right)\alpha\kappa^2(t)A_t, \quad P_{gQ} \sim Poisson(\gamma Q_t), \tag{S.12}$$

$$P_{rQ} \sim Poisson(\rho_A Q_t), \quad P_{rH} \sim Poisson(\rho_H(t)H_t), \quad P_d \sim Poisson(\delta(t)H_t), \tag{S.13}$$

$$\Delta A_t = -(P_{tA} + P_{gA} + P_{rA}) + P_{aA}, \quad \Delta Q_t = P_{tA} - (P_{gQ} + P_{rQ}), \tag{S.14}$$

$$\Delta H_t = P_{gA} + P_{gQ} - (P_{rH} + P_d), \quad \Delta D_t = P_d, \quad \Delta C_t = P_{tA} + P_{gA}, \tag{S.15}$$

$$\Delta S_t = -\Delta A_t, \quad \Delta R_t^{reported} = \Delta R_t^Q + \Delta R_t^H = P_{rQ} + P_{rH}. \tag{S.16}$$

In the above simulation (S.10) – (S.16) are the Poisson process random variable versions of the population model (1) – (7) (see Section 2.1) Here, we assume T_t, κ_t to be given. Also, in compliance with assumption **A5**, we take $\rho_Q(t) \equiv \rho_A$. Further, in (S.16) we use

$\Delta R_t^{reported}$ instead of $\Delta R_t = \Delta R_t^A + \Delta R_t^Q + \Delta R_t^H$ since only $R_t^{reported} = R_t^Q + R_t^H$ can be observed or reported in practice. The values for $\Delta A_t, \Delta H_t, \Delta Q_t$ can be negative.

At the beginning of the pandemic, the number of individuals in the symptomatic or quarantined states is quite low, introducing an inherent bias in the estimated trajectories. We use an aggregation procedure to increase the size of the simulated samples.

The general idea of the aggregation method is to make use of the fixed intrinsic parameters such as γ and ρ_A to generate 10 independent processes as 10 locations, for example, counties or cities, and consider the loss function to be the sum of the loss of these 10 independent processes. For the ith state or process, we have the loss metric $d_s^{(i)}$ as in (S.2) and the new locally weighted loss function conditional on $\boldsymbol{\zeta} = (\gamma, \rho_A)$ is constructed as

$$\text{(S.17)} \qquad \widetilde{L}_t^h(\boldsymbol{\beta}(t)|\boldsymbol{\zeta}) = \sum_{i=1}^{10} \sum_s \frac{1}{h} K\left(\frac{t-s}{h}\right) d_s^{(i)}(\boldsymbol{\beta}(t)|\boldsymbol{\zeta}).$$

The time-varying parameters are estimated as the minimizer of (S.17), while the intrinsic parameters are found as the optimizers of the corresponding profile loss function using a grid search algorithm.

S6.2. **Parametric bootstrap algorithm.** We now describe a parametric bootstrap method to construct pointwise confidence intervals for the estimated rate parameters and other relevant epidemiological quantities.

Step 1. Given the estimates of the parameters $\widehat{\boldsymbol{\beta}}(t)$, $\widehat{\boldsymbol{\zeta}}$ and $\widehat{\alpha}$ (assuming κ_t is observed), simulate multiple copies of the data from a Poisson process with the dynamical states given by (1) – (7) in Section 2.1, by substituting the true parameter values with the estimates, over the same time domain, treating T_t (number of tests) as given (since this is an intervention process that does not have a discernible generative mechanism).

Step 2. For the k-th sample, obtain the bootstrap estimates $\widehat{\boldsymbol{\beta}}^{*k}(t)$, $\widehat{\boldsymbol{\zeta}}^{*k}$ and $\widehat{\alpha}^{*k}$.

Step 3. Use percentile bootstrap procedure to find the confidence intervals (pointwise C.I. for the time-dependent parameters respectively).

S6.3. **Simulated data and results.** The simulation setting is studied for $T = 100$ days for 10 independent processes. The true parameters generating the processes are chosen as follows. The time-invariant parameters are $\gamma = 0.03$, indicating that on a certain day 3 out of 100 asymptomatic individuals may become symptomatic, $\rho_A = 0.06$, meaning daily 6 among 100 asymptomatic individuals may recover, and $\alpha = 0.2$ as the baseline infection rate, which suggests 20 of 100 susceptible individuals may be infected without social distancing. The recovering rate from state H_t is time-dependent: 0.03 for the first 20 days, 0.04 for the following 40 days, and 0.05 for the rest of the days. The parameter $\phi(t)$, testing efficiency, is a linear function of time T with 0.00005 as a small positive slope and 0.001 as the intercept which reflects increasing testing efficiency. The intrinsic parameters and mobility index are fixed across all processes. The mobility index κ_t is also a time-dependent parameter, ranging from 0.4 to 1.2 to describe the overall social distancing of people. $\delta(t)$ represents time-dependent death rate over 100 days. It is set as 0.007 during the first 50 days while 0.005 during the following 50 days. That is, 7 out of $1,000$ hospitalized individuals may die during the first half of the period while 5 out of $1,000$ hospitalized ones may die during the second half. Each process will be assigned to different numbers of test cases.

S6.3.1. *Parametric bootstrap.* We first conduct a grid search for γ and ρ_A to estimate the optimal parameters minimizing the loss function (see (20) in Section 3.1) This yields

$$\hat{\gamma} = 0.03, \hat{\rho}_A = 0.058.$$

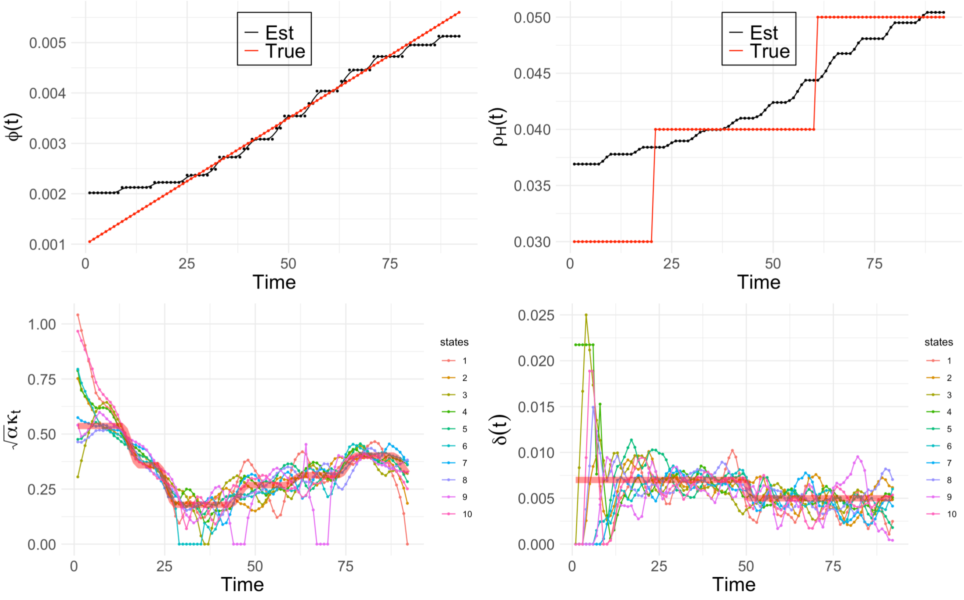

FIGURE 1. From left to right : plots of estimations for $\phi(t), \rho_H(t)$, $\sqrt{\alpha}\kappa_t$, and $\delta(t)$; the wide red lines denote the true $\sqrt{\alpha}\kappa_t$ and $\delta(t)$

We then estimate the other time-dynamic parameters by using the values of $\hat{\gamma}, \hat{\rho}_A$.

We observe that the estimates of γ and ρ_A are very close to our true values. Figure 1 reports the estimates against the true parameters. $\hat{\phi}(t)$ and $\widehat{\rho_H}(t)$ are quite close to the true ones, with a bias smaller than 0.01 and capturing the overall pattern of change. The estimates of $\sqrt{\alpha}\kappa_t$ and $\delta(t)$ are varying across the 10 processes, however, the overall trends are captured well.

Using the parametric bootstrap procedure (Section S6), we compute the bootstrap sampling distributions and confidence regions for various epidemiological parameters and compare them with the true values of the underlying parameters. Under the same setting, we now generate 1000 independent copies of the aggregation processes with the estimated values of the parameters and repeat the estimation procedure. This yields a set of resampled estimates for the parameters from which the bootstrap sampling distributions can be computed. We notice that, (See Figure 2) the 95% confidence interval constructed based on the bootstrap sampling distribution contains the true values of $\gamma = 0.03$ and $\rho_A = 0.06$.

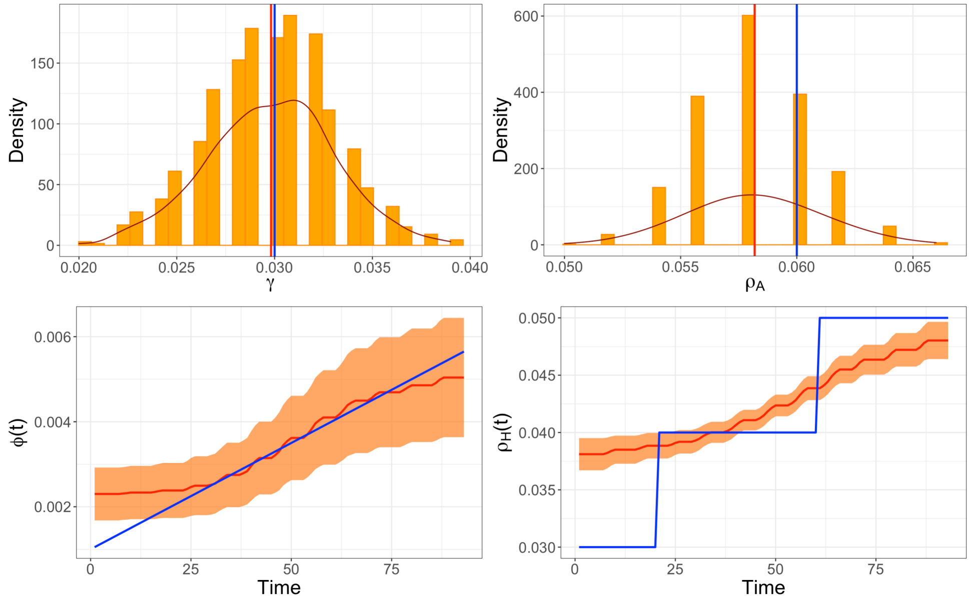

FIGURE 2. From left to right: the top row displays the sampling distribution of γ and ρ_A - the bootstrap sample mean and the true parameter values are depicted in the red line and the blue lines respectively. The bottom row show the 95% point-wise confidence intervals for $\phi(t)$ and $\rho_H(t)$ based on 1000 bootstrap samples- the bootstrap sample means are shown in red while the blue curves are the true parameters.

It is also found that the true $\phi(t)$ lies within the corresponding confidence band uniformly over time. The true $\rho_H(t)$ intersects partially with the corresponding confidence band.

The 95% point-wise confidence band for A_t with its true trajectory is shown in Figure 3. It seems that the confidence region covers the true A_t well. Similar results are observed (not displayed here) for the other compartments like C_t etc.

We can conclude, based on the simulation results, that the Poisson process model could be able to describe the dynamics pretty well, as long as we have enough data and valid information. If such a model is true, the parametric bootstrap method can be employed to compute the confidence intervals of the estimates. However, due to the limited sample size and possible lack of valid information, the data we encounter in real life is noisier, resulting in biased estimates if the Poisson process model is applied directly.

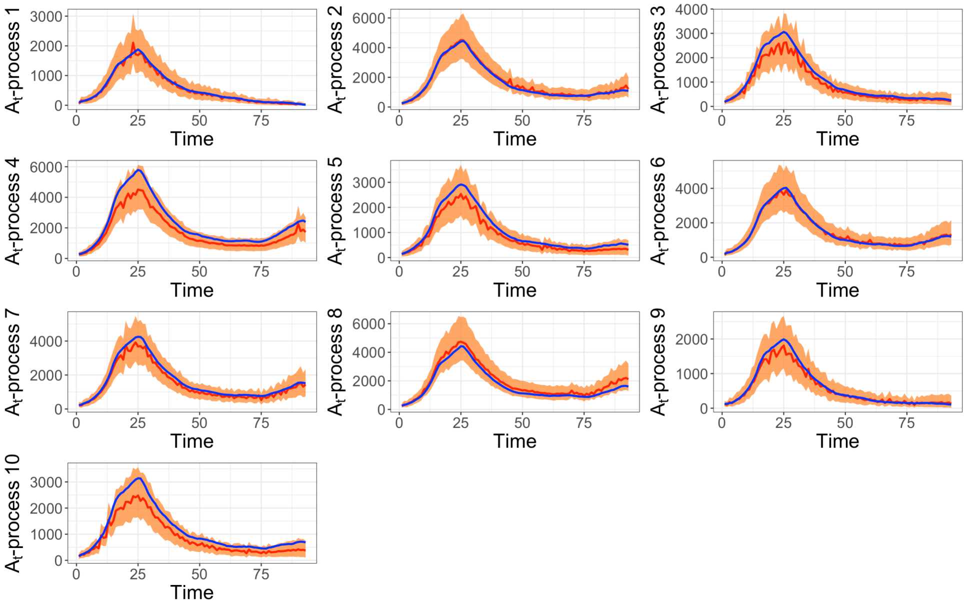

FIGURE 3. Point-wise Confidence band for A_t for all 10 independent processes; point-wise mean is in red and the true trajectories are in blue.

S6.3.2. *Residual bootstrap.* We apply the residual bootstrap strategy as described in Section S6.3) to the same simulation setting (and compare the performances of the two types of bootstrap inference. The results for the residual bootstrap are presented in Figures 4 and 5.

In general, the confidence intervals of the parameters and the compartments obtained from the residual bootstrap are wider than those computed from the parametric bootstrap. It should however be noted that in both methods the confidence intervals more or less cover the true value of the parameters and the observed and estimated compartments. Even though a narrower band can be obtained, the parametric bootstrap can only be justified when the observed data supports the underlying assumption of the Poisson process mechanism. The residual bootstrap, being nonparametric on the other hand, is data-driven, robust, and hence more adaptable to the current data analysis paradigm, despite the fact that the confidence regions of parameters provided are wider.

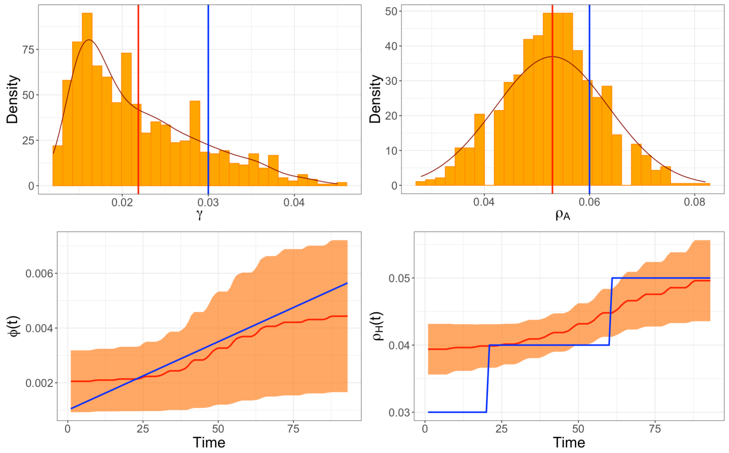

FIGURE 4. From left to right: the top row displays the sampling distribution of γ and ρ_A - the bootstrap sample mean and the true parameter values are depicted in the red line and the blue lines respectively. The bottom row show the 95% point-wise confidence intervals for $\phi(t)$ and $\rho_H(t)$ based on 1000 bootstrap samples- the bootstrap sample means are shown in red while the blue curves are the true parameters.

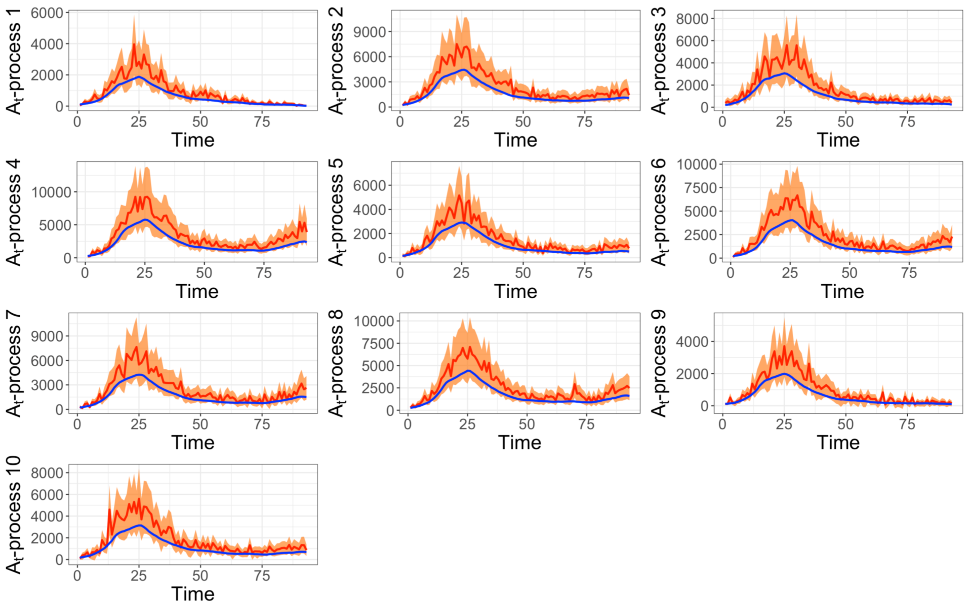

FIGURE 5. Point-wise Confidence Interval for A_t for all 10 independent processes; point-wise mean is in red and the true data is in blue.

S7. Practical implications of the proposed model

It is generally believed that an increase in testing together with subsequent quarantining can play a significant role in containing the pandemic. Even though many traditional SIER models control for some kind of social mixing (e.g. [1]), to our knowledge, most models exclude the explicit effects of testing and subsequent quarantining. For this reason, the effect of the number of tests has never been quantified in any analysis involving real COVID-19 pandemic data. Incorporating information on testing can lead to scientific conclusions with profound implications in public policy. Numerical simulation of the pandemic based on the estimates obtained from our model shows explicitly that, with all the time-invariant and time-varying rate parameters remaining the same, a higher testing rate leads to suppression and eventual decline in the number of infected individuals as well as hospitalizations and deaths.

For the purposes of demonstration, let $\phi_0(t)$, $T_H^0(t)$ be respectively the estimated testing efficiency, observed daily test to daily hospitalization ratio on day t from the pandemic data reported by *Utah*. Let

$$\theta_f(t) = f \times \phi_0(t) \times T_H^0(t). \tag{S.18}$$

To understand the effects of increased testing rate, we simulate the course of the pandemic based on our Poissonian compartment model (see Supplement Section $S6.1.$ equations (S.11) - (S.17)) from August 15 to December 6, 2020, for different values of f, while using the parameter estimates for the US state *Utah*. This is achieved by setting the $\Delta T_t = f \times T_H^0(t) \times H_t$, where H_t is the number of hospitalizations on day t in the simulated trajectories. Figure 6 and Figure 7 illustrate the effect of the testing factor $f = 1.1$ (10% increase in testing) and $f = 1.3$ (30% increase in testing) respectively. For the first scenario, the trajectories of C_t and D_t are closer to the observed ones, even though

the rate of their growths are slower. Significant reductions in the numbers of H_t and A_t (asymptomatic but infected individuals) are noticed from the beginning of November. This modest increase of 10%, shows relatively little effect but shows that the trajectories simulated from the underlying Poisson model can approximate the observed number of confirmed cases and deaths.

In comparison, the curves are clearly flattened when the confirmed fraction is increased by 30%, i.e. when $f = 1.3$. Non-increasing patterns shown in the cumulative compartments, C_t, and D_t indicate a containment of the disease. The cumulative number of deaths is also significantly smaller than the observed death by December 6, 2020. Both H_t and A_t. seem to decrease almost to zero.

For records, the intensive care units in *Utah* reached more than 100% capacity around $17th$ October 2020. From a public policy perspective, testing and subsequent quarantine might be easier to implement than many other social distancing measures.

Most of the models for real COVID dynamics data that we came across are based on either SIR, SEIR, SIRD, or one of its variants. They include only a few compartments. As we have noted above important features and potential control variables like testing, social distancing, use of masks, etc. are often ignored. In order to fit the observed data restrictive and usually untestable distributional assumptions are routinely made. The temporally static or dynamic model parameters are often estimated using tools from machine learning and Bayesian literature [2, 3, 4], which are computationally intensive and their results are usually difficult to interpret. Results from more detailed multi-compartment models are usually based on simulation, not real data sets.

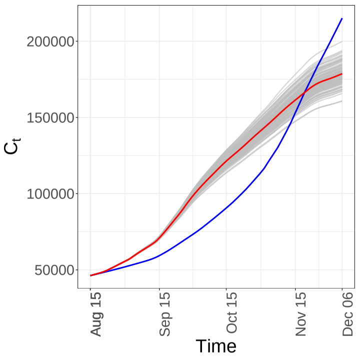

(a) C_t

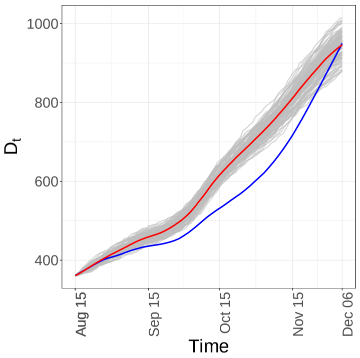

(b) D_t

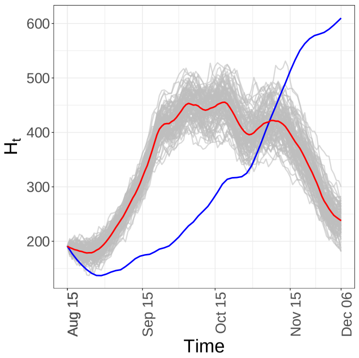

(c) H_t

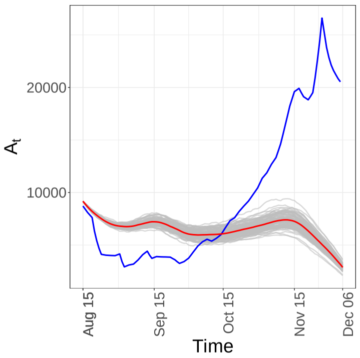

(d) A_t

FIGURE 6. The 100 simulated trajectories when testing rate is increased by 10% are in grey while the mean of them are presented in red. The observed C_t, D_t, H_t and estimated A_t in Utah are marked in blue.

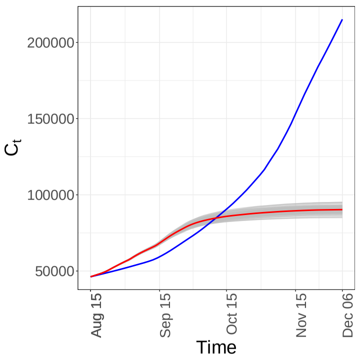

(a) C_t

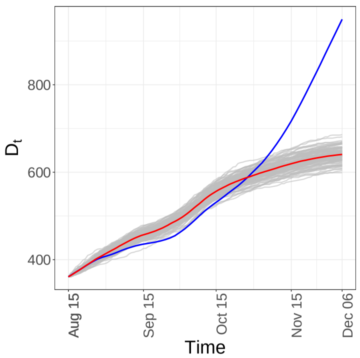

(b) D_t

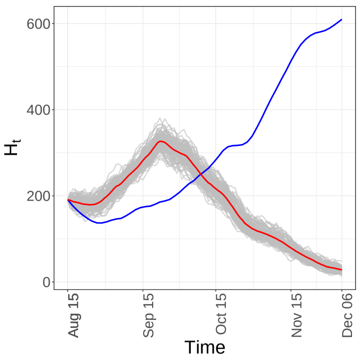

(c) H_t

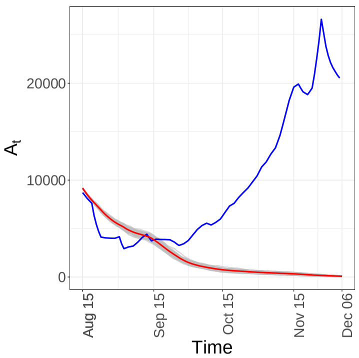

(d) A_t

FIGURE 7. The 100 simulated trajectories when testing rate is increased by 30% are in cray while the mean of them are presented in red. The observed C_t, D_t, H_t and estimated A_t in Utah are marked in blue.

Along with the various time-invariant and time-varying rate parameters, we propose some analogues of popular epidemiological markers. These markers are mostly based on the estimated number of asymptomatic infected people.

For example, a time-dynamic version of the Basic Reproduction Rate can be computed using the detailed, multi-compartment model we consider (see equation (15) in Section 2.2.6.) By focusing on the compartment A_t, new infections arrive at the compartment at the rate of $\alpha\kappa_t^2 S_t/(S_t + A_t + R_t)$ and leave at the rate of $(\theta(t) + \gamma + \rho_A)$. There is no other pathway for disease spread. Thus, the proposed $\tilde{R}_0(t)$ can be interpreted in the same way as the conventional basic reproduction rate.

Our analogue of this epidemiological marker seems more realistic since it tallies with the other observed and estimated compartments. For example, around August 7, 2020, the Cumulative New Infections (CNI), both observed and estimated, hospitalization and the asymptomatic population (estimated) were quite low and almost constant over a period of time (Figure 8(a)) The estimated social mobility index $\alpha\kappa_t^2$ also depicted a sharp decline around that time (Figure 8(b)), which all give evidence to the fact that the spread of the pandemic was indeed contained around mid-July to mid- August in Utah. This is clearly resonated in our version of reproduction rate but is not so well captured by the two other models considered above. The estimate released by IHME [1] seems to follow our estimate in August, however, it hardly gets higher than 1, not even in October, when the number of new infections was high. From this, it seems that the IHME estimate does not qualitatively reflect the real nature of COVID spread.

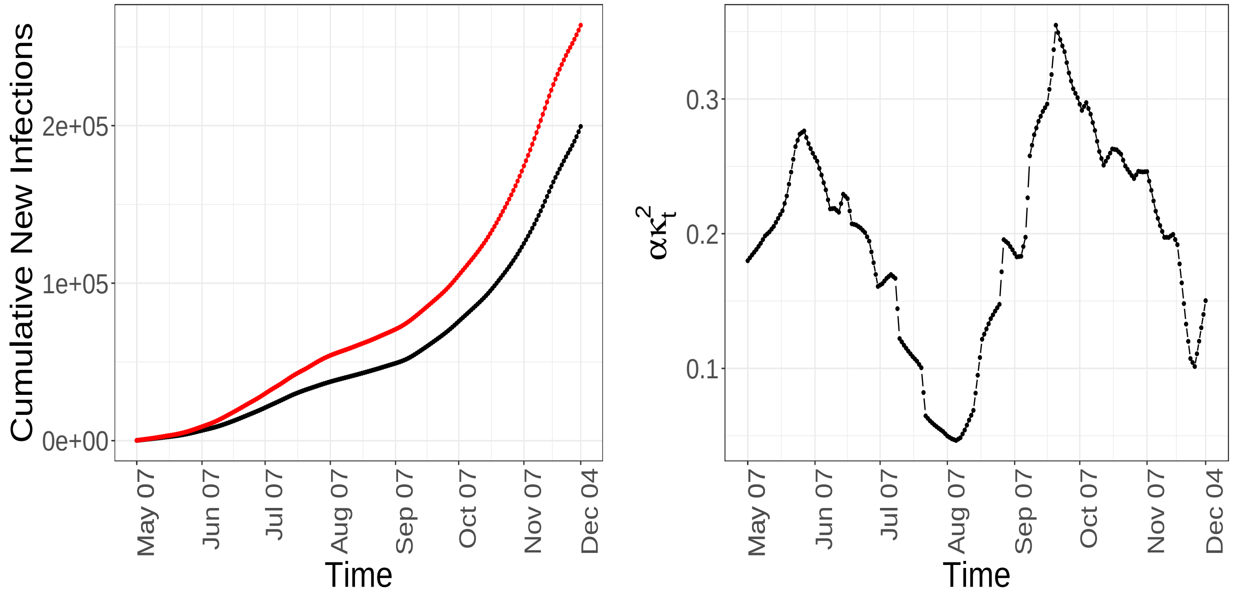

(a) Cumulative New Infections for *Utah*; observed (−), fitted (−)

(b) Estimates of a social mobility index $\alpha\kappa_t^2$ for *Utah*

FIGURE 8. Temporal patterns of some compartments and epidemiological markers for Utah.

S8. Additional results for a select few states of the US

The table comparing the estimates for the relevant parameters for some chosen states of the US (Table 3 in Section 4.2.9) is extended to include the estimates of α - the basic infection rate without any social distancing measure put in place, and $\phi(t)$ - the time-varying parameter testing efficiency. To this end, the estimate of α is found from (S.9), as described in Supplement Section S5, using the google mobility data as a surrogate for $\kappa(t)$.

The Google community mobility trends data measures changes in the frequency and length of stay at different locations relative to a baseline level prior to the COVID-19 pandemic. The baseline activity level is defined as the median activity value for the corresponding day of the week in the period of Jan. 3 to Feb. 6, 2020 during which time most countries (excluding China) did not implement any distancing efforts. Available

	$\hat{\gamma}$	$\hat{\rho}_A$	$\hat{\alpha}$	$\widehat{\delta(t)}$	$\widehat{\rho_H(t)}$	$\widehat{\theta(t)}$	$\widehat{\phi(t)}$
Arizona	0.0003	0.002	0.1509	0.0208	0.0023	0.0887	0.0079
Arkansas	0.0029	0.094	0.1775	0.0249	0.0975	0.0809	0.0038
Delaware	0.0017	0.008	0.1519	0.0159	0.0093	0.1076	0.0037
Idaho	0.0009	0.010	0.0357	0.0230	0.0138	0.0289	0.0019
Iowa	0.0011	0.032	0.0682	0.0263	0.0372	0.0478	0.0033
Minnesota	0.0023	0.128	0.2410	0.0315	0.0654	0.0899	0.0034
Nebraska	0.0011	0.020	0.0557	0.0141	0.0266	0.0394	0.0035
Ohio	0.0023	0.048	0.1027	0.0180	0.0532	0.0625	0.0024
Oklahoma	0.0037	0.084	0.1372	0.0122	0.1029	0.0494	0.0033
Pennsylvania	0.0013	0.026	0.0818	0.0293	0.0372	0.0535	0.0033
South Dakota	0.0021	0.058	0.0561	0.0190	0.0922	0.0262	0.0038
Tennessee	0.0059	0.064	0.1907	0.0158	0.0413	0.1206	0.0076
Texas	0.0019	0.036	0.0986	0.0207	0.0341	0.0212	0.0013
Utah	0.0011	0.040	0.1692	0.0144	0.0252	0.1434	0.0061
Wisconsin	0.0017	0.068	0.0926	0.0217	0.0707	0.0477	0.0026

TABLE 1. Mean estimated parameters for different states in the US.

categories include retail, grocery, park, transit, workplace, and residential categories. In particular, information on the change in the mobility patterns, as the percentage decrease (increase) from the baseline, in different areas such as parks, residential locations, retail locations, among others during the pandemic from the Google mobility database can be obtained. The publicly available data was sourced from https://www.google.com/covid19/mobility/ (accessed on December 15, 2020).

The mean estimates for the time varying parameters $\delta(t)$, $\rho_H(t)$, $\theta(t)$, and $\phi(t)$ are shown (see Table 1).

We now present the point estimates, as well as the residual bootstrap results, for the relevant parameters and epidemiological markers pertaining to a select few US states. The results can be interpreted in a similar way as for the case of Utah in Section 4.2.

Arizona

	Estimate	95% Confidence Interval	Mean	s.d.
γ	0.0003	[0.0003, 0.0005]	0.0003	0.00007
ρ_A	0.0020	[0.0019, 0.0025]	0.0022	0.0003
α	0.1509	[0.1519, 0.1727]	0.1599	0.0061

Table 2. Confidence intervals, mean and standard deviations for the time-invariant parameters, computed based on 1000 bootstrap samples using residual bootstrap approach for *Arizona*.

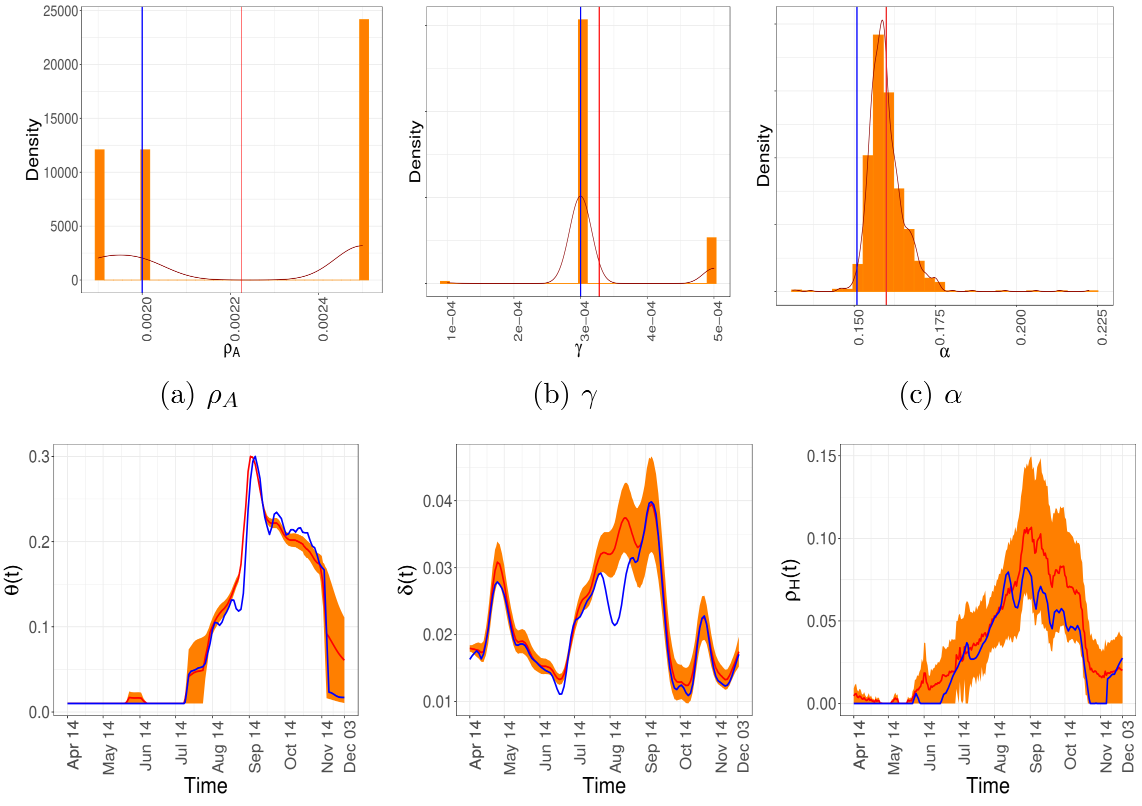

(a) ρ_A (b) γ (c) α

(d) $\theta(t)$ (e) $\delta(t)$ (f) $\rho_H(t)$

Figure 9. Estimates and residual bootstrap based confidence intervals for time invariant and time-varying parameters for the state of *Arizona*. The estimate from the data is in blue. The 95% confidence band is in yellow and the mean of the bootstrap estimates are presented in red.

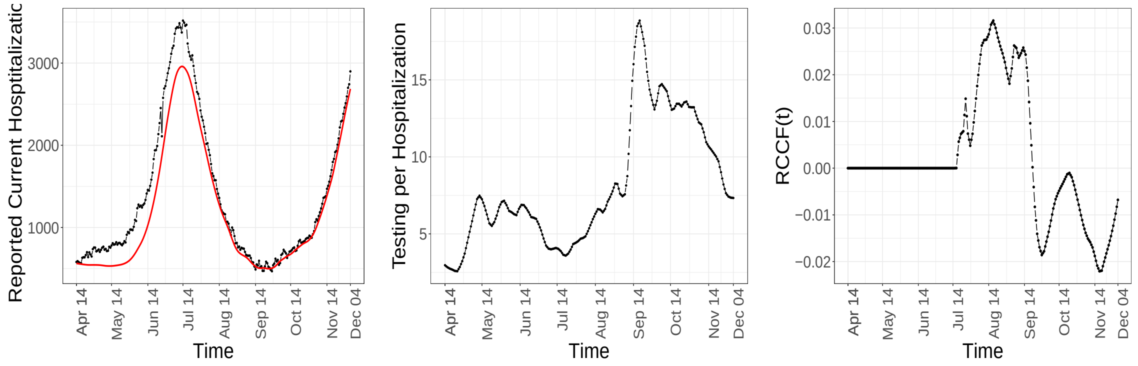

(a) Daily hospitalizations, observed (—), fitted (—)

(b) $\Delta T_t/H_t$,

(c) RCCF

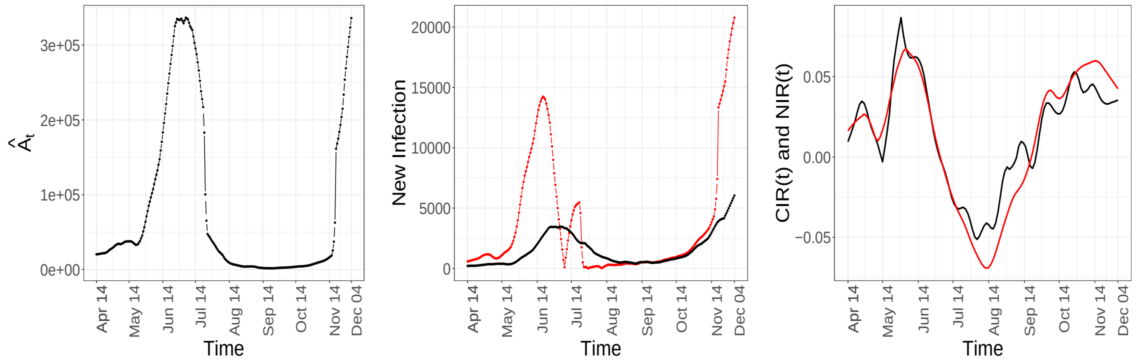

(d) $\hat{A}_t$

(e) Daily new infections, observed (—), fitted (—)

(f) CIR (—) and NIR (—)

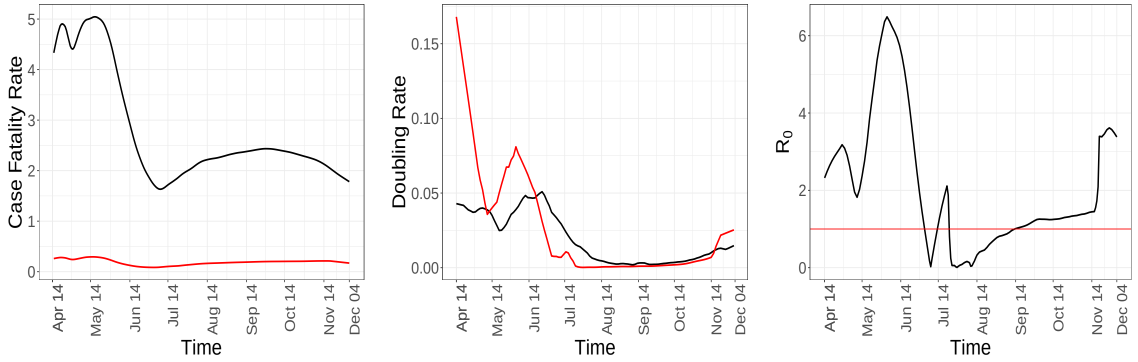

(g) Fatality Rate, observed (—), actual (—)

(h) Doubling rate, C_t (—) and CNI (—)

(i) R_0 analogue

FIGURE 10. Temporal patterns of some components and epidemiological markers for *Arizona*.

Arkansas

	Estimate	95% Confidence Interval	Mean	s.d.
γ	0.0029	[0.0021, 0.0039]	0.0027	0.0005
ρ_A	0.0940	[0.0840, 0.1200]	0.0979	0.0090
α	0.1775	[0.1744, 0.2170]	0.1924	0.0110

TABLE 3. Confidence intervals, mean and standard deviations for the time-invariant parameters, computed based on 1000 bootstrap samples using residual bootstrap approach for *Arkansas*.

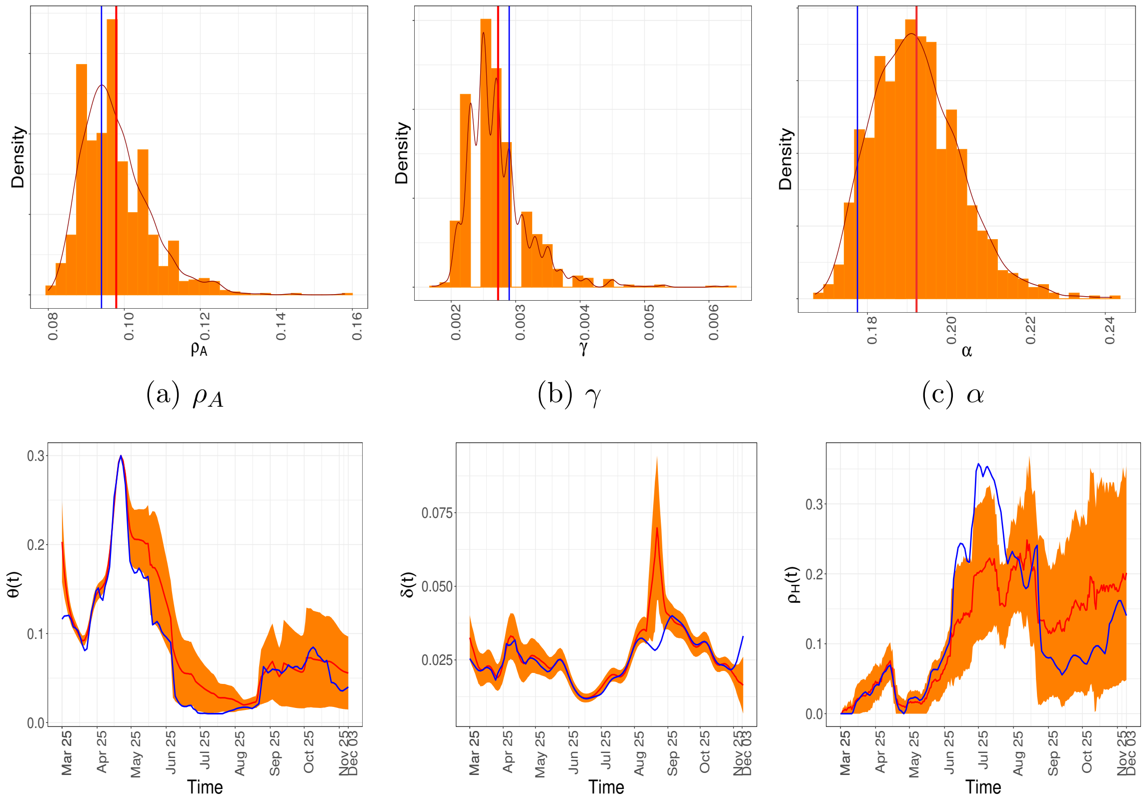

(a) ρ_A (b) γ (c) α

(d) $\theta(t)$ (e) $\delta(t)$ (f) $\rho_H(t)$

FIGURE 11. Estimates and residual bootstrap based confidence intervals for time invariant and time-varying parameters for the state of *Arkansas*. The estimate from the data is in blue. The 95% confidence band is in yellow and the mean of the bootstrap estimates are presented in red.

The point estimates and bootstrap confidence for the relevant parameters are presented below. The mortality and hospitalization rate in Arkansas is high.

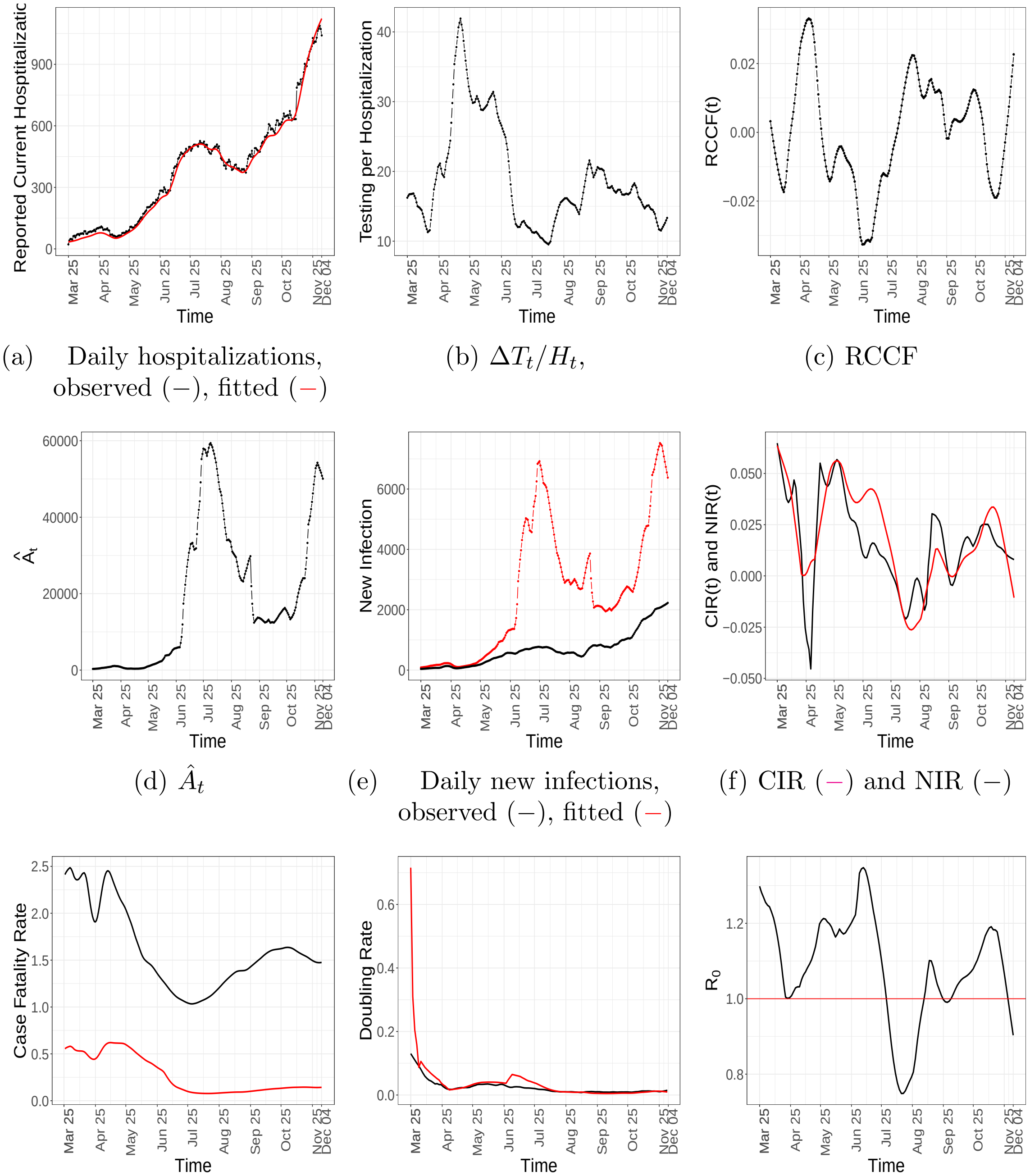

(a) Daily hospitalizations, observed (–), fitted (–)

(b) $\Delta T_t / H_t$,

(c) RCCF

(d) $\hat{A}_t$

(e) Daily new infections, observed (–), fitted (–)

(f) CIR (–) and NIR (–)

(g) Fatality Rate, observed (–), actual (–)

(h) Doubling rate, C_t (–) and CNI (–)

(i) R_0 analogue

FIGURE 12. Temporal patterns of some components and epidemiological markers for *Arkansas*.

Delaware

	Estimate	95% Confidence Interval	Mean	s.d.
γ	0.0017	[0.0001, 0.0211]	0.0024	0.0045
ρ_A	0.0080	[0.0080, 0.0100]	0.0084	0.0008
α	0.1519	[0.0465, 0.1478]	0.0970	0.0257

TABLE 4. Confidence intervals, mean and standard deviations for the time-invariant parameters, computed based on 1000 bootstrap samples using residual bootstrap approach for *Delaware*.

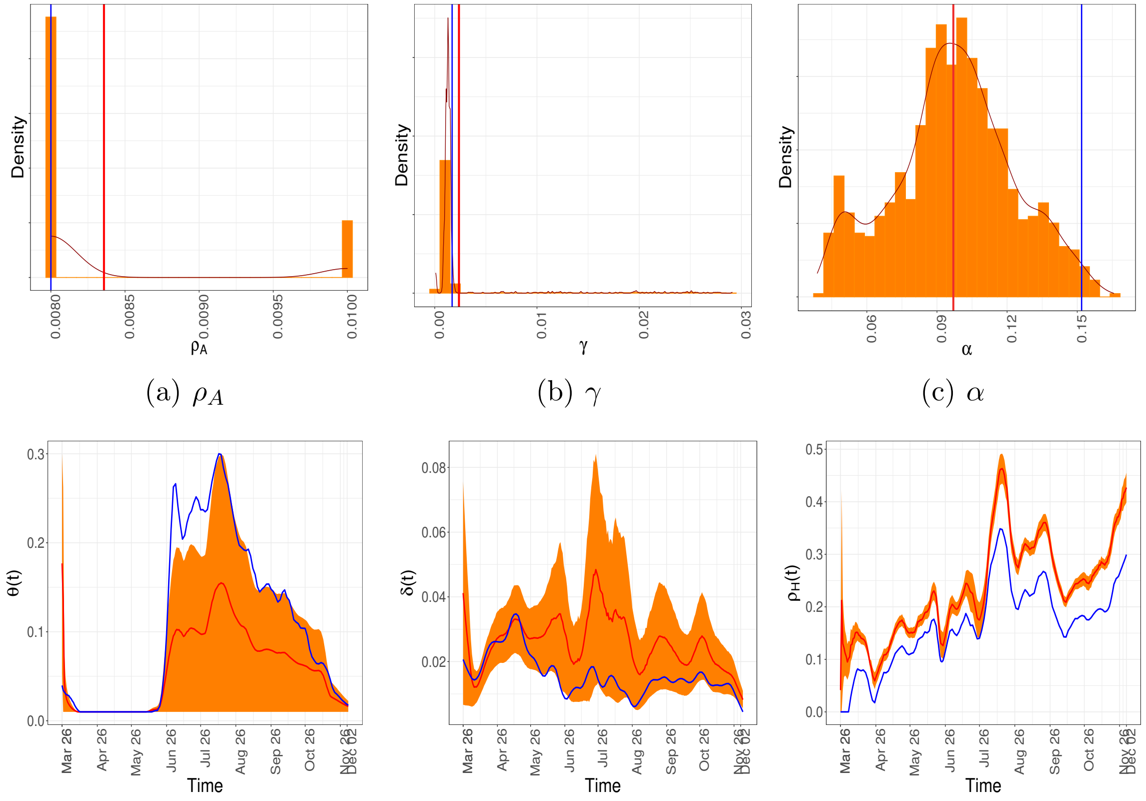

(a) ρ_A (b) γ (c) α

(d) $\theta(t)$ (e) $\delta(t)$ (f) $\rho_H(t)$

FIGURE 13. Estimates and residual bootstrap based confidence intervals for time invariant and time-varying parameters for the state of *Delaware*. The estimate from the data is in blue. The 95% confidence band is in yellow and the mean of the bootstrap estimates are presented in red.

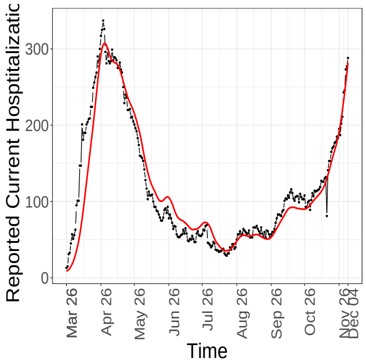

(a) Daily hospitalizations, observed (−), fitted (−)

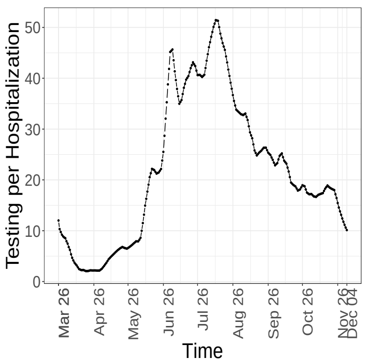

(b) $\Delta T_t/H_t$,

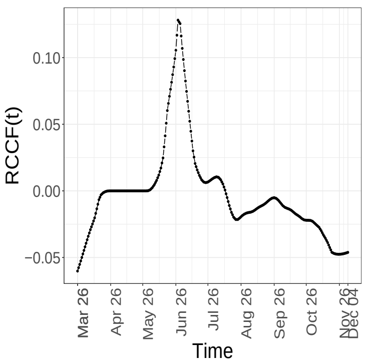

(c) RCCF

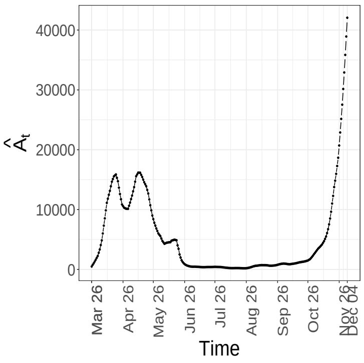

(d) $\hat{A}_t$

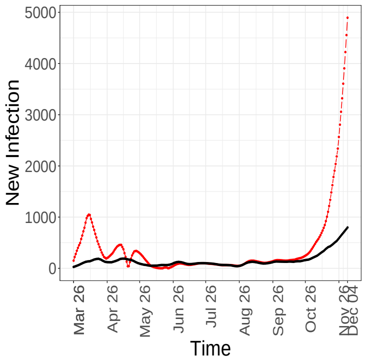

(e) Daily new infections, observed (−), fitted (−)

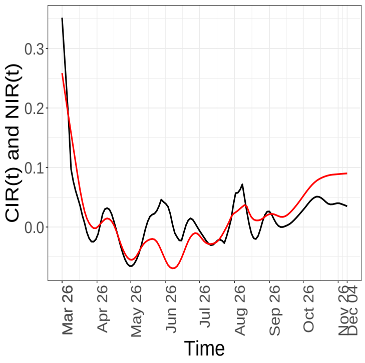

(f) CIR (−) and NIR (−)

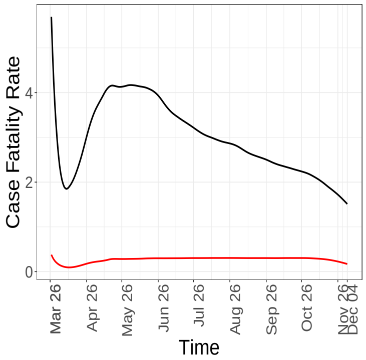

(g) Fatality Rate, observed (−), actual (−)

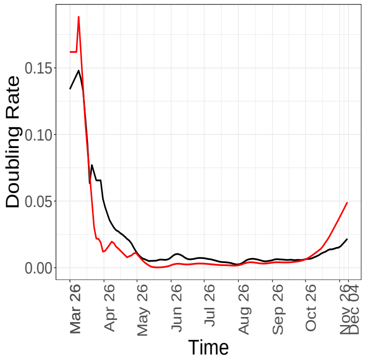

(h) Doubling rate, C_t (−) and CNI (−)

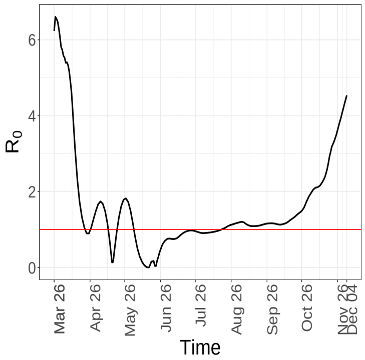

(i) R_0 analogue

FIGURE 14. Temporal patterns of some components and epidemiological markers for *Delaware*.

Idaho

	Estimate	95% Confidence Interval	Mean	s.d.
γ	0.0009	[0.0005, 0.0019]	0.0012	0.0004
ρ_A	0.0100	[0.0080, 0.0120]	0.0097	0.0013
α	0.0357	[0.0382, 0.0609]	0.0503	0.0061

TABLE 5. Confidence intervals, mean and standard deviations for the time-invariant parameters, computed based on 1000 bootstrap samples using residual bootstrap approach for *Idaho*.

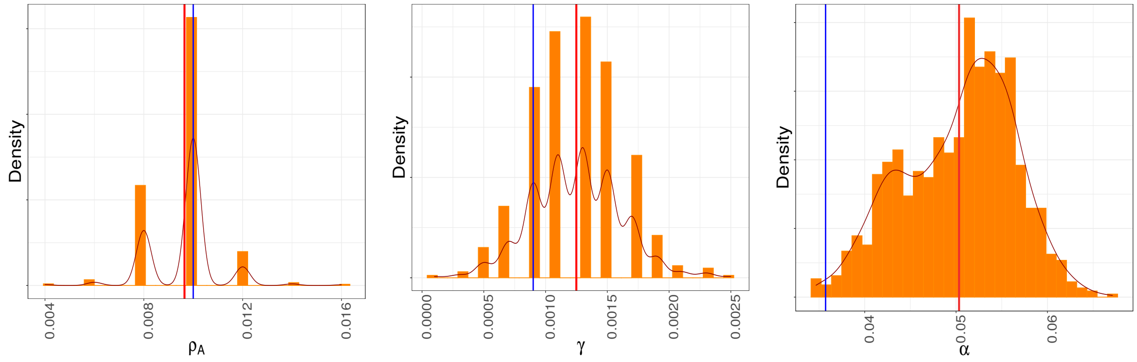

(a) ρ_A (b) γ (c) α

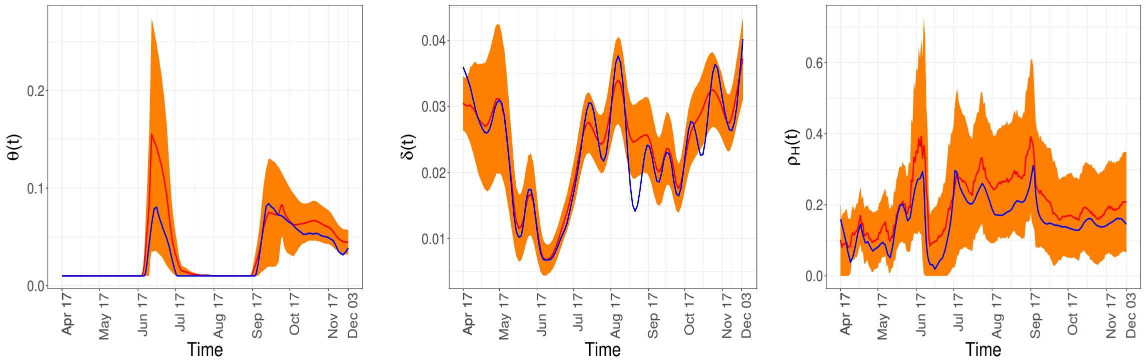

(d) $\theta(t)$ (e) $\delta(t)$ (f) $\rho_H(t)$

FIGURE 15. Estimates and residual bootstrap based confidence intervals for time invariant and time-varying parameters for the state of *Idaho*. The estimate from the data is in blue. The 95% confidence band is in yellow and the mean of the bootstrap estimates are presented in red.

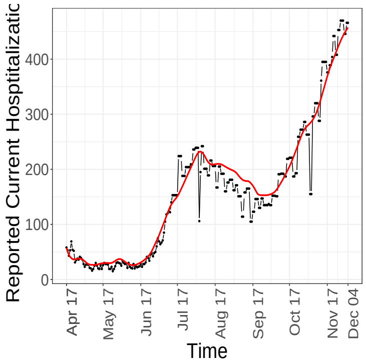

(a) Daily hospitalizations, observed (–), fitted (–)

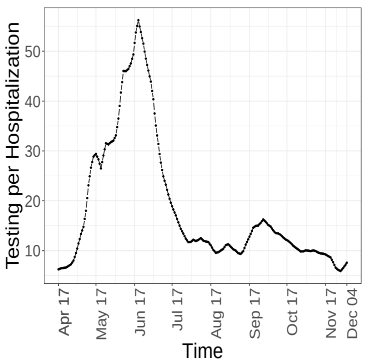

(b) $\Delta T_t/H_t$,

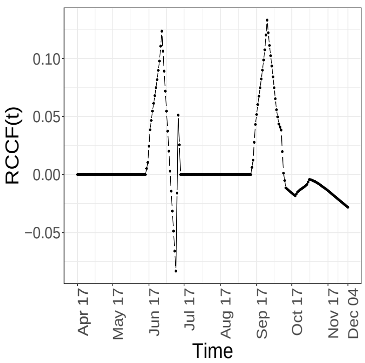

(c) RCCF

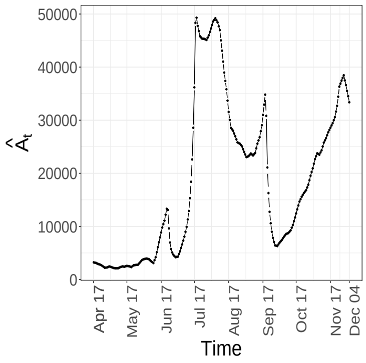

(d) $\hat{A}_t$

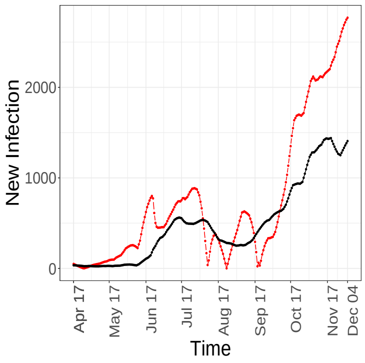

(e) Daily new infections, observed (–), fitted (–)

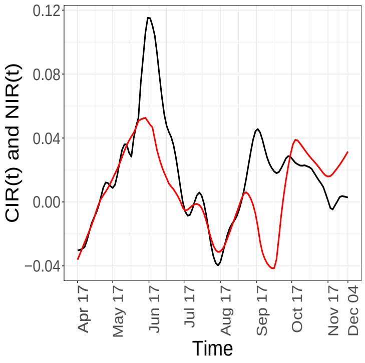

(f) CIR (–) and NIR (–)

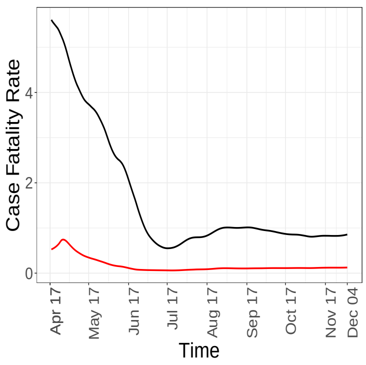

(g) Fatality Rate, observed (–), actual (–)

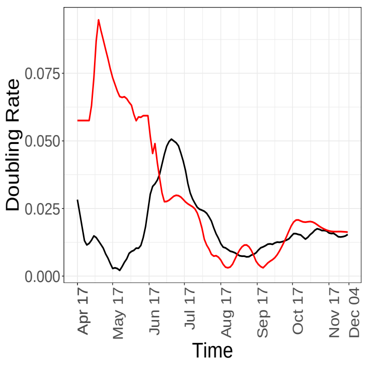

(h) Doubling rate, C_t (–) and CNI (–)

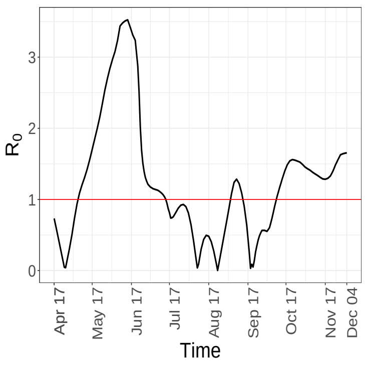

(i) R_0 analogue

FIGURE 16. Temporal patterns of some components and epidemiological markers for *Idaho*.

Iowa

	Estimate	95% Confidence Interval	Mean	s.d.
γ	0.0011	[0.0009, 0.0095]	0.0022	0.0019
ρ_A	0.0320	[0.0120, 0.0520]	0.0270	0.0103
α	0.0682	[0.0371, 0.1019]	0.0595	0.0170

TABLE 6. Confidence intervals, mean and standard deviations for the time-invariant parameters, computed based on 1000 bootstrap samples using residual bootstrap approach for *Iowa*.

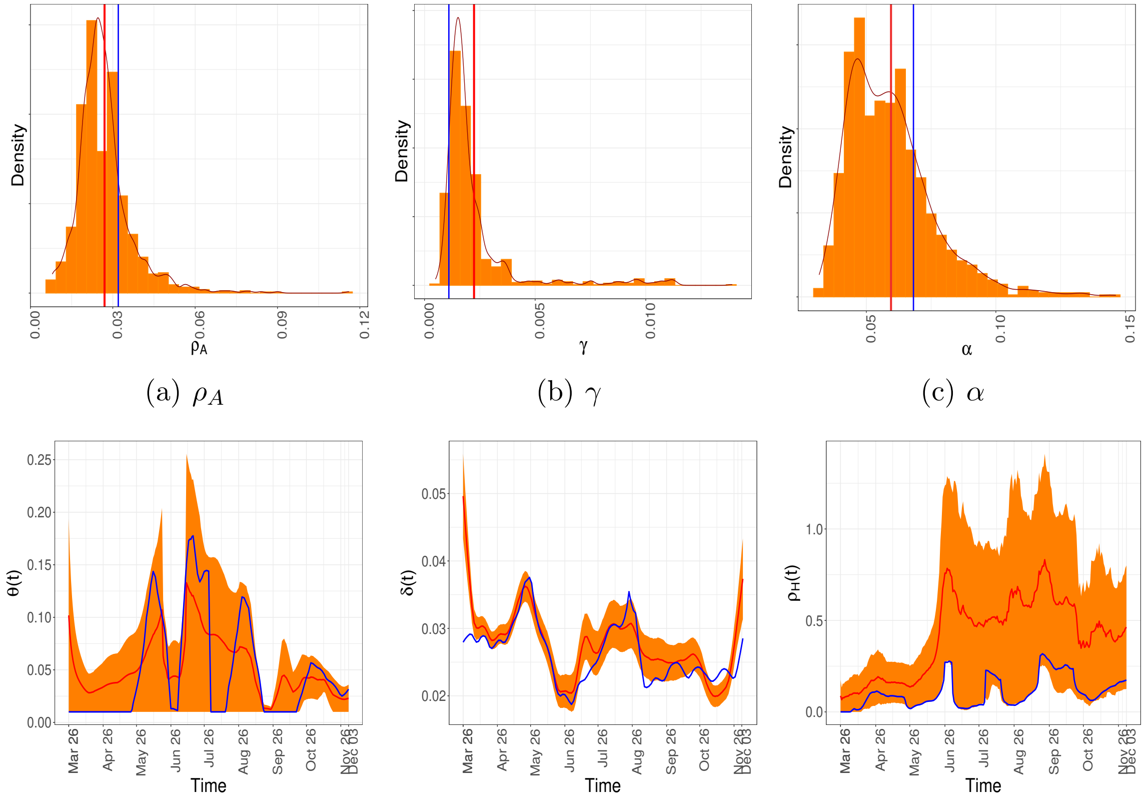

(a) ρ_A (b) γ (c) α

(d) $\theta(t)$ (e) $\delta(t)$ (f) $\rho_H(t)$

FIGURE 17. Estimates and residual bootstrap based confidence intervals for time invariant and time-varying parameters for the state of *Iowa*. The estimate from the data is in blue. The 95% confidence band is in yellow and the mean of the bootstrap estimates are presented in red.

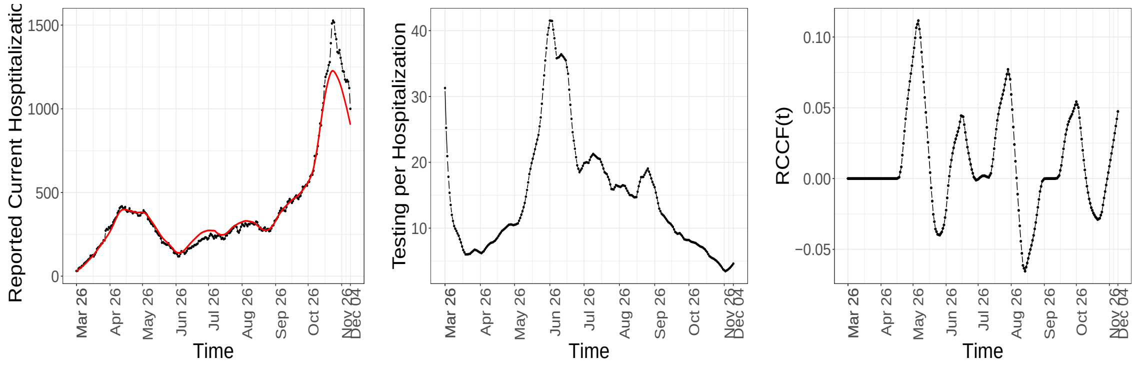

(a) Daily hospitalizations, observed (–), fitted (–)

(b) $\Delta T_t/H_t$,

(c) RCCF

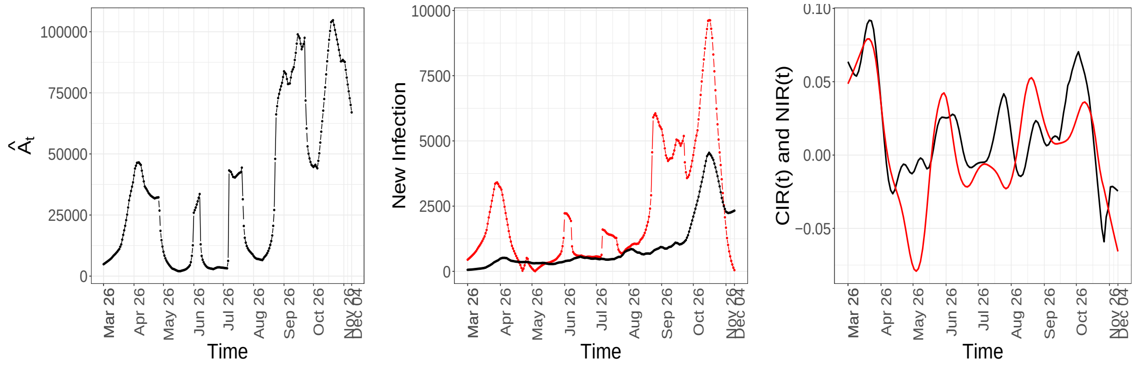

(d) $\hat{A}_t$

(e) Daily new infections, observed (–), fitted (–)

(f) CIR (–) and NIR (–)

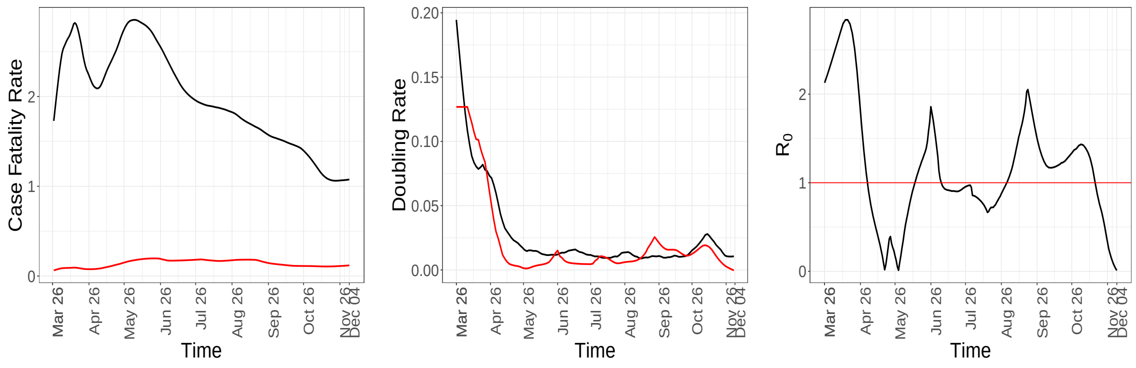

(g) Fatality Rate, observed (–), actual (–)

(h) Doubling rate, C_t (–) and CNI (–)

(i) R_0 analogue

FIGURE 18. Temporal patterns of some components and epidemiological markers for *Iowa*.

Minnesota

	Estimate	95% Confidence Interval	Mean	s.d.
γ	0.0023	[0.0013, 0.0045]	0.0024	0.0008
ρ_A	0.1280	[0.1020, 0.1620]	0.1255	0.0150
α	0.2410	[0.1785, 0.2664]	0.2244	0.0232

TABLE 7. Confidence intervals, mean and standard deviations for the time-invariant parameters, computed based on 1000 bootstrap samples using residual bootstrap approach for *Minnesota*.

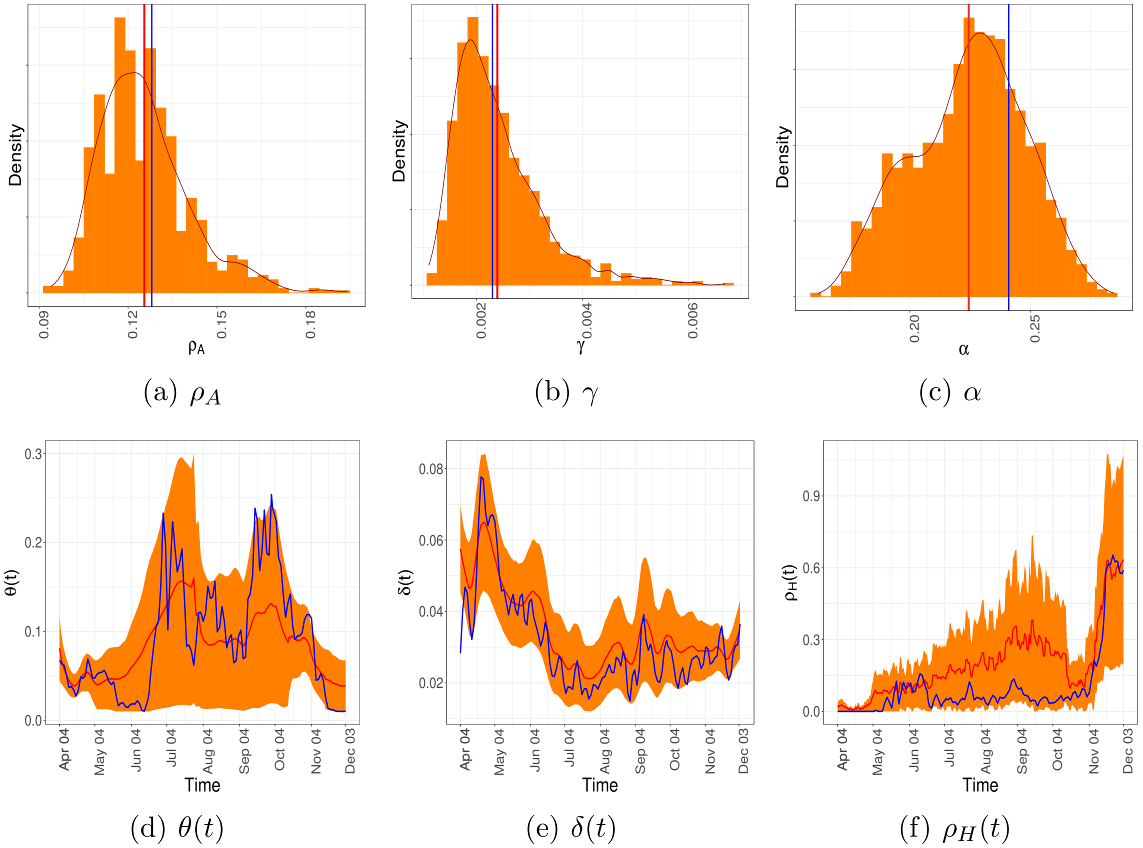

(a) ρ_A (b) γ (c) α

(d) $\theta(t)$ (e) $\delta(t)$ (f) $\rho_H(t)$

FIGURE 19. Estimates and residual bootstrap based confidence intervals for time invariant and time-varying parameters for the state of *Minnesota*. The estimate from the data is in blue. The 95% confidence band is in yellow and the mean of the bootstrap estimates are presented in red.

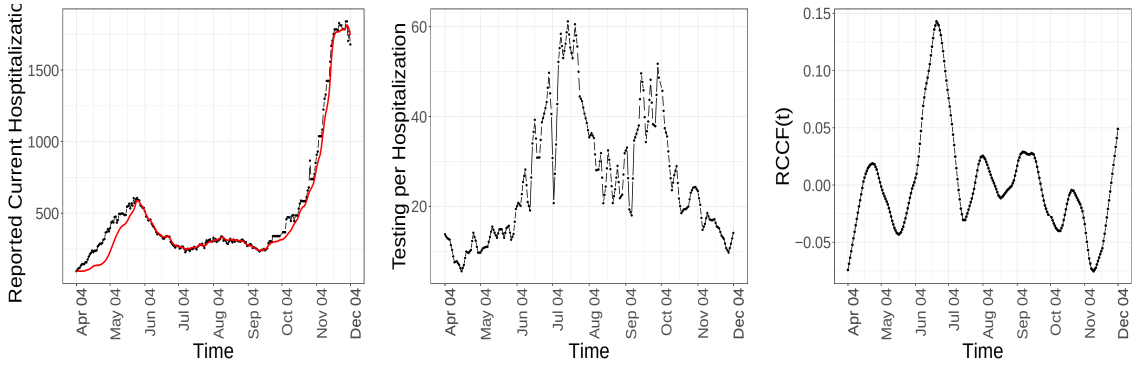

(a) Daily hospitalizations, observed (−), fitted (−) (b) $\Delta T_t/H_t$, (c) RCCF

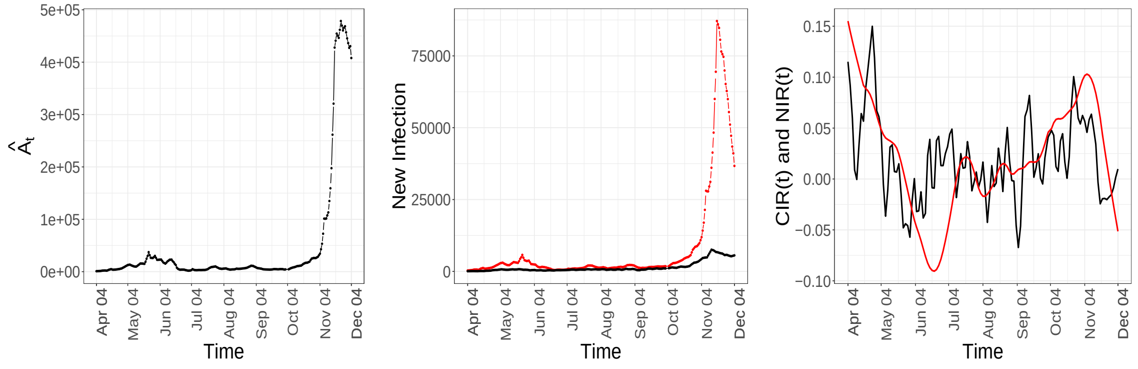

(d) $\hat{A}_t$ (e) Daily new infections, observed (−), fitted (−) (f) CIR (−) and NIR (−)

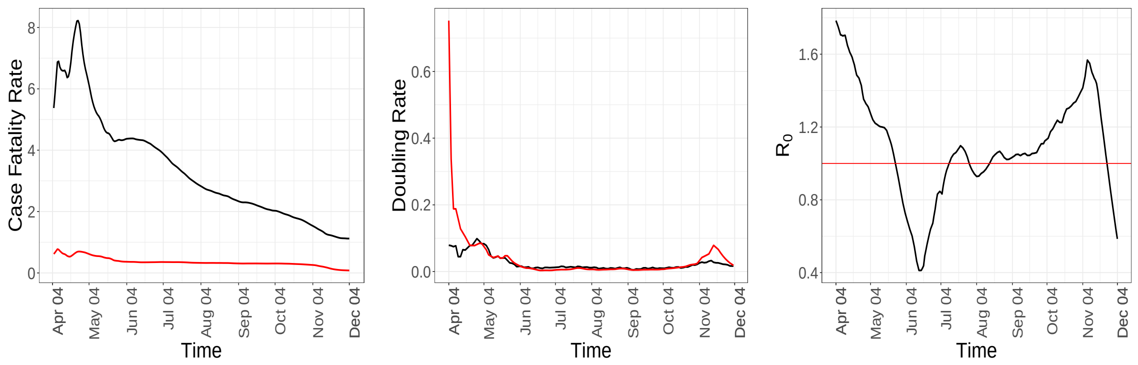

(g) Fatality Rate, observed (−), actual (−) (h) Doubling rate, C_t (−) and CNI (−) (i) R_0 analogue

FIGURE 20. Temporal patterns of some components and epidemiological markers for *Minnesota*.

Nebraska

	Estimate	95% Confidence Interval	Mean	s.d.
γ	0.0011	[0.0009, 0.0019]	0.0016	0.0013
ρ_A	0.0200	[0.0140, 0.0240]	0.0174	0.0112
α	0.0557	[0.0324, 0.0934]	0.0545	0.1189

TABLE 8. Confidence intervals, mean and standard deviations for the time-invariant parameters, computed based on 1000 bootstrap samples using residual bootstrap approach for *Nebraska*.

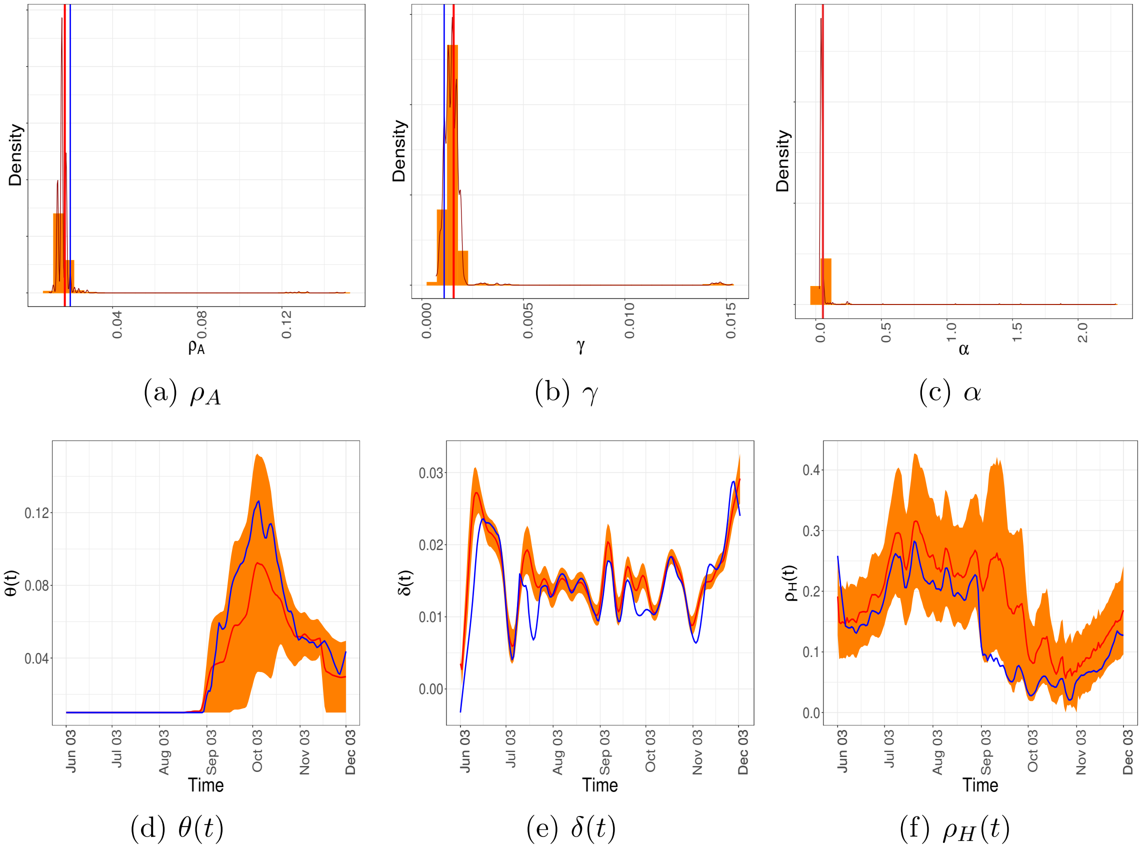

(a) ρ_A (b) γ (c) α

(d) $\theta(t)$ (e) $\delta(t)$ (f) $\rho_H(t)$

FIGURE 21. Estimates and residual bootstrap based confidence intervals for time invariant and time-varying parameters for the state of *Nebraska*. The estimate from the data is in blue. The 95% confidence band is in yellow and the mean of the bootstrap estimates are presented in red.

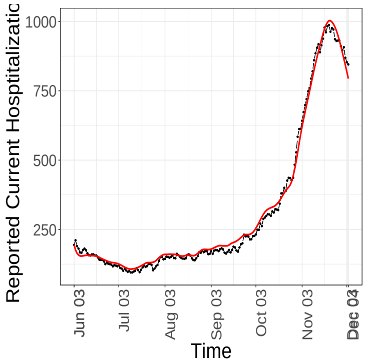

(a) Daily hospitalizations, observed (–), fitted (–)

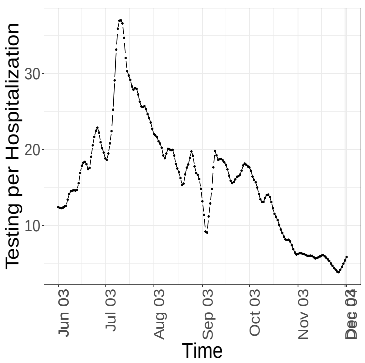

(b) $\Delta T_t / H_t$,

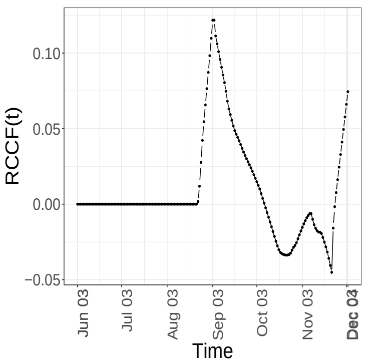

(c) RCCF

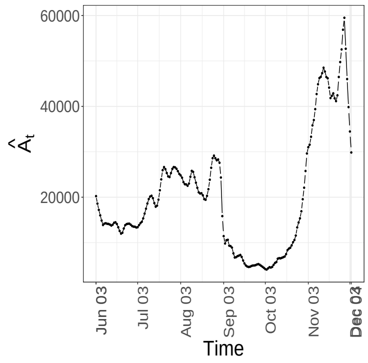

(d) $\hat{A}_t$

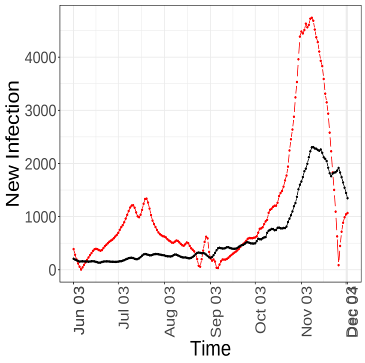

(e) Daily new infections, observed (–), fitted (–)

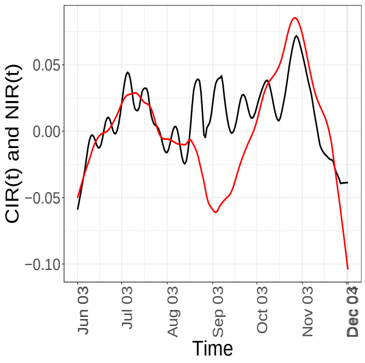

(f) CIR (–) and NIR (–)

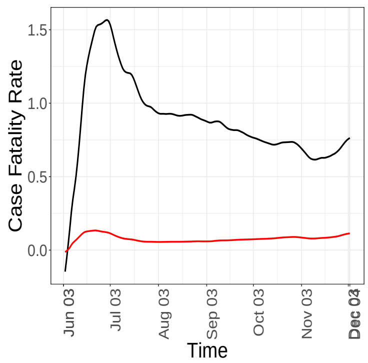

(g) Fatality Rate, observed (–), actual (–)

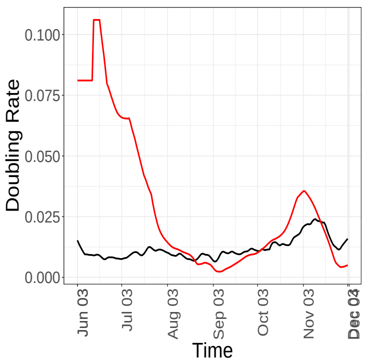

(h) Doubling rate, C_t (–) and CNI (–)

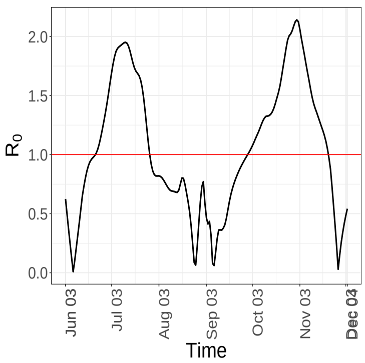

(i) R_0 analogue

FIGURE 22. Temporal patterns of some components and epidemiological markers for *Nebraska*.

Ohio

	Estimate	95% Confidence Interval	Mean	s.d.
γ	0.0023	[0.0019, 0.0029]	0.0024	0.0003
ρ_A	0.0480	[0.0440, 0.0500]	0.0471	0.0014
α	0.1027	[0.0881, 0.1133]	0.0977	0.0063

Table 9. Confidence intervals, mean and standard deviations for the time-invariant parameters, computed based on 1000 bootstrap samples using residual bootstrap approach for *Ohio*.

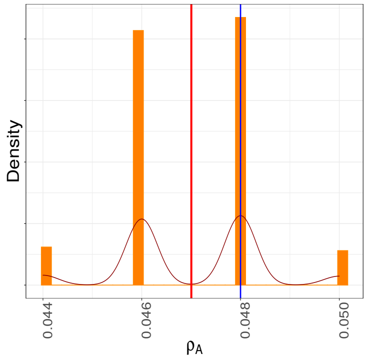

(a) ρ_A

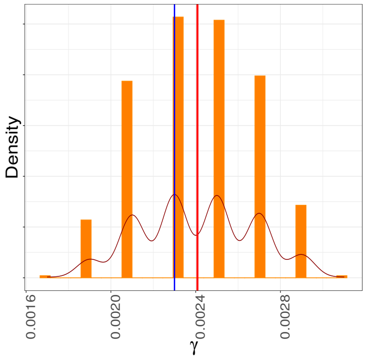

(b) γ

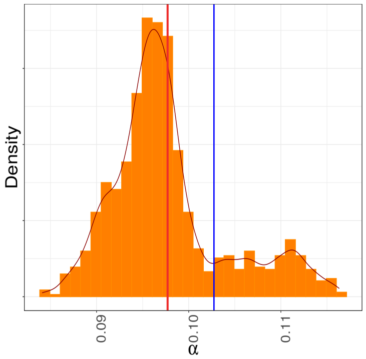

(c) α

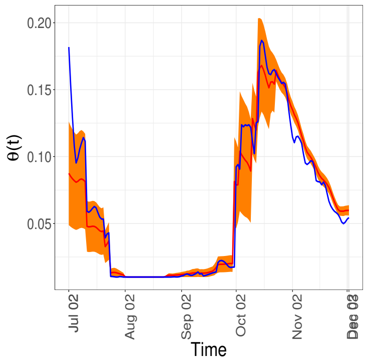

(d) $\theta(t)$

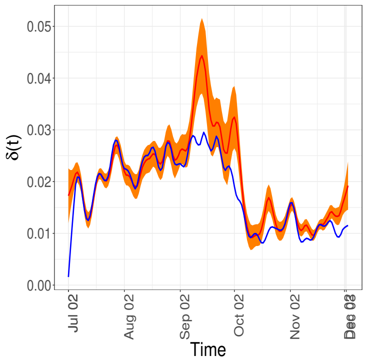

(e) $\delta(t)$

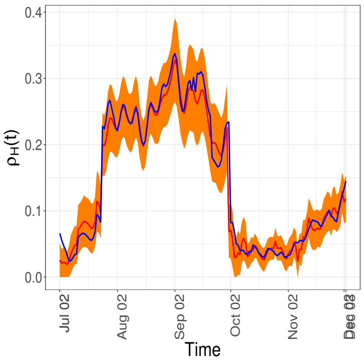

(f) $\rho_H(t)$

Figure 23. Estimates and residual bootstrap based confidence intervals for time invariant and time-varying parameters for the state of *Ohio*. The estimate from the data is in blue. The 95% confidence band is in yellow and the mean of the bootstrap estimates are presented in red.

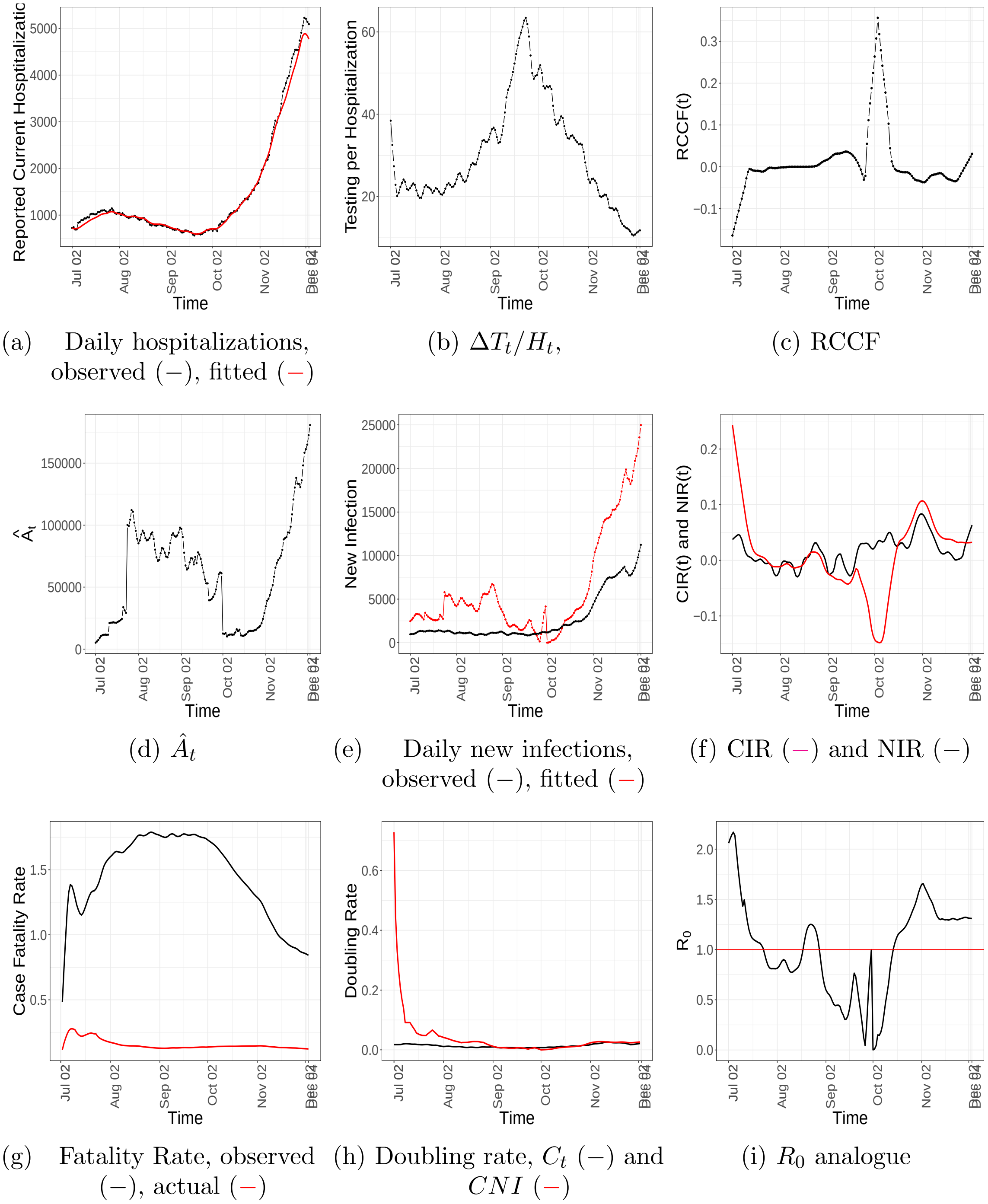

(a) Daily hospitalizations, observed (–), fitted (–)

(b) $\Delta T_t/H_t$,

(c) RCCF

(d) $\hat{A}_t$

(e) Daily new infections, observed (–), fitted (–)

(f) CIR (–) and NIR (–)

(g) Fatality Rate, observed (–), actual (–)

(h) Doubling rate, C_t (–) and CNI (–)

(i) R_0 analogue

FIGURE 24. Temporal patterns of some components and epidemiological markers for *Ohio*.

Oklahoma

	Estimate	95% Confidence Interval	Mean	s.d.
γ	0037	[0.0043, 0.0055]	0.0048	0.0003
ρ_A	0.0840	[0.0740, 0.0880]	0.0812	0.0036
α	0.1372	[0.1815, 0.2048]	0.1935	0.0059

TABLE 10. Confidence intervals, mean and standard deviations for the time-invariant parameters, computed based on 1000 bootstrap samples using residual bootstrap approach for *Oklahoma*.

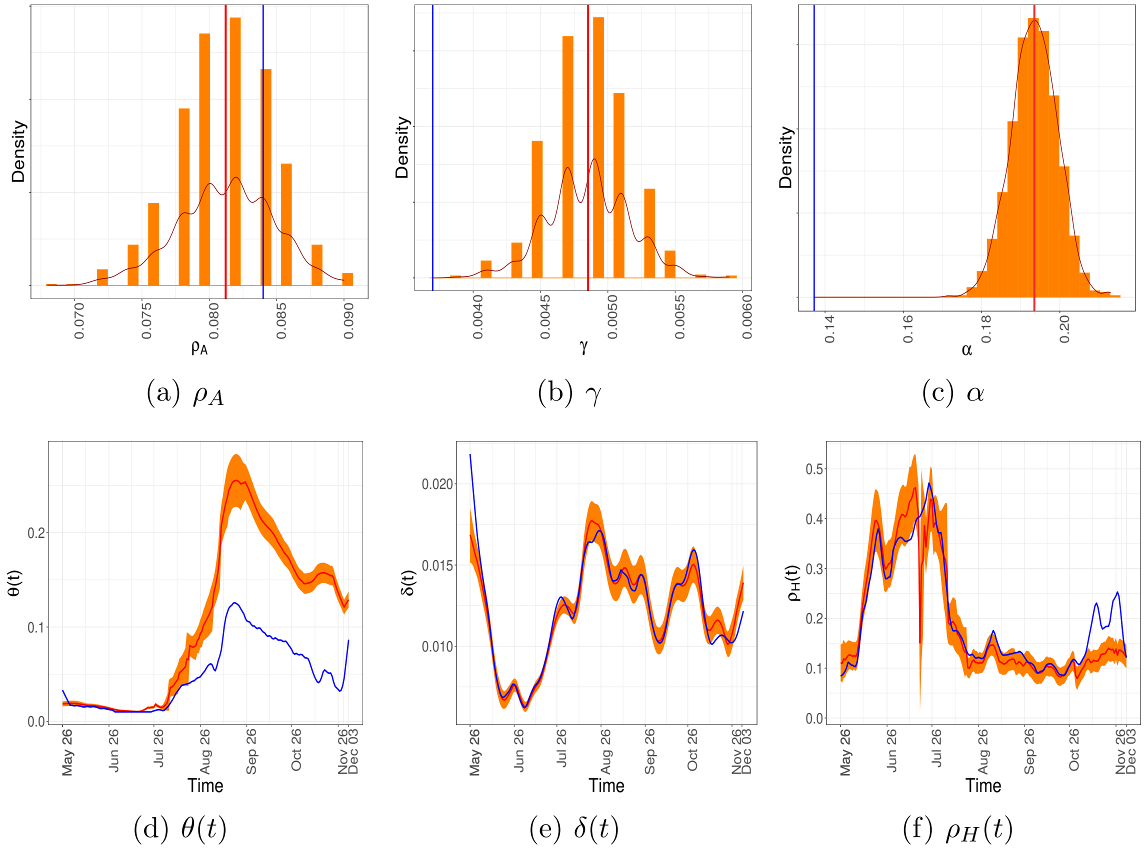

(a) ρ_A (b) γ (c) α

(d) $\theta(t)$ (e) $\delta(t)$ (f) $\rho_H(t)$

FIGURE 25. Estimates and residual bootstrap based confidence intervals for time invariant and time-varying parameters for the state of *Oklahoma*. The estimate from the data is in blue. The 95% confidence band is in yellow and the mean of the bootstrap estimates are presented in red.

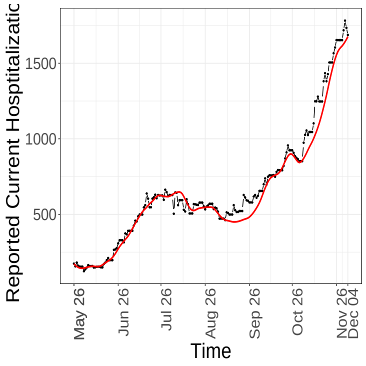

(a) Daily hospitalizations, observed (−), fitted (−)

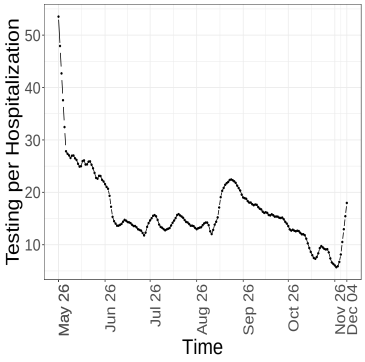

(b) $\Delta T_t/H_t$,

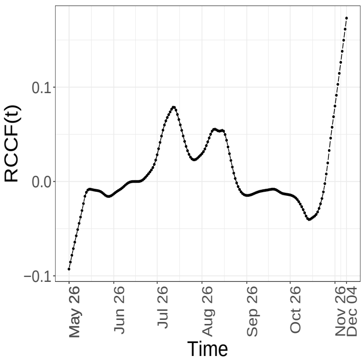

(c) RCCF

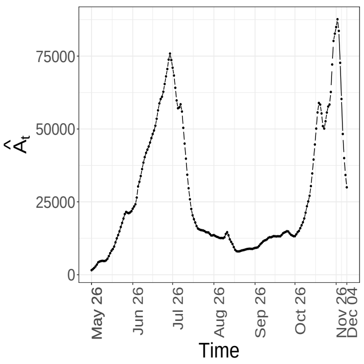

(d) $\hat{A}_t$

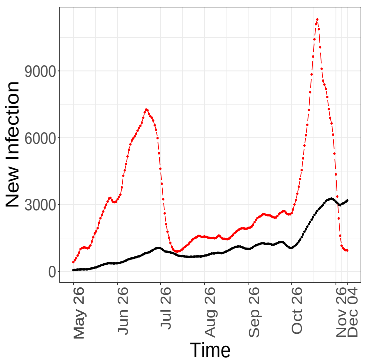

(e) Daily new infections, observed (−), fitted (−)

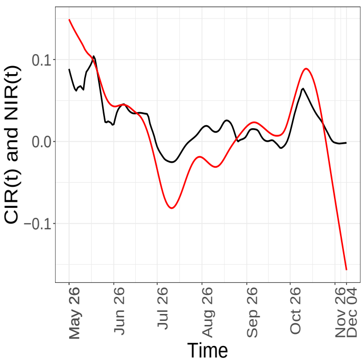

(f) CIR (−) and NIR (−)

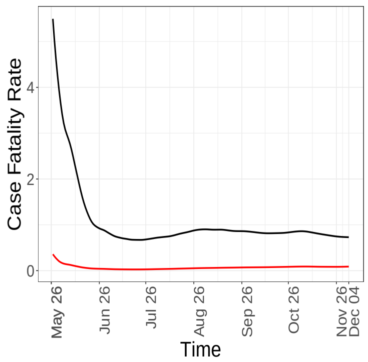

(g) Fatality Rate, observed (−), actual (−)

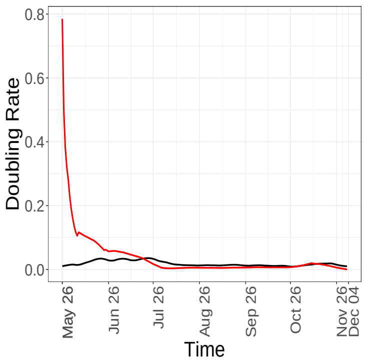

(h) Doubling rate, C_t (−) and CNI (−)

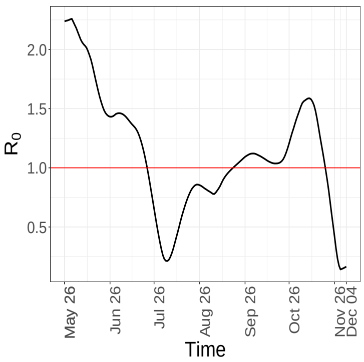

(i) R_0 analogue

FIGURE 26. Temporal patterns of some components and epidemiological markers for *Oklahoma*.

Pennsylvania

	Estimate	95% Confidence Interval	Mean	s.d.
γ	0.0013	[0.0009, 0.0019]	0.0014	0.0003
ρ_A	0.0260	[0.0260, 0.0300]	0.0279	0.0007
α	0.0818	[0.0965, 0.1407]	0.1173	0.0109

TABLE 11. Confidence intervals, mean and standard deviations for the time-invariant parameters, computed based on 1000 bootstrap samples using residual bootstrap approach for *Pennsylvania*.

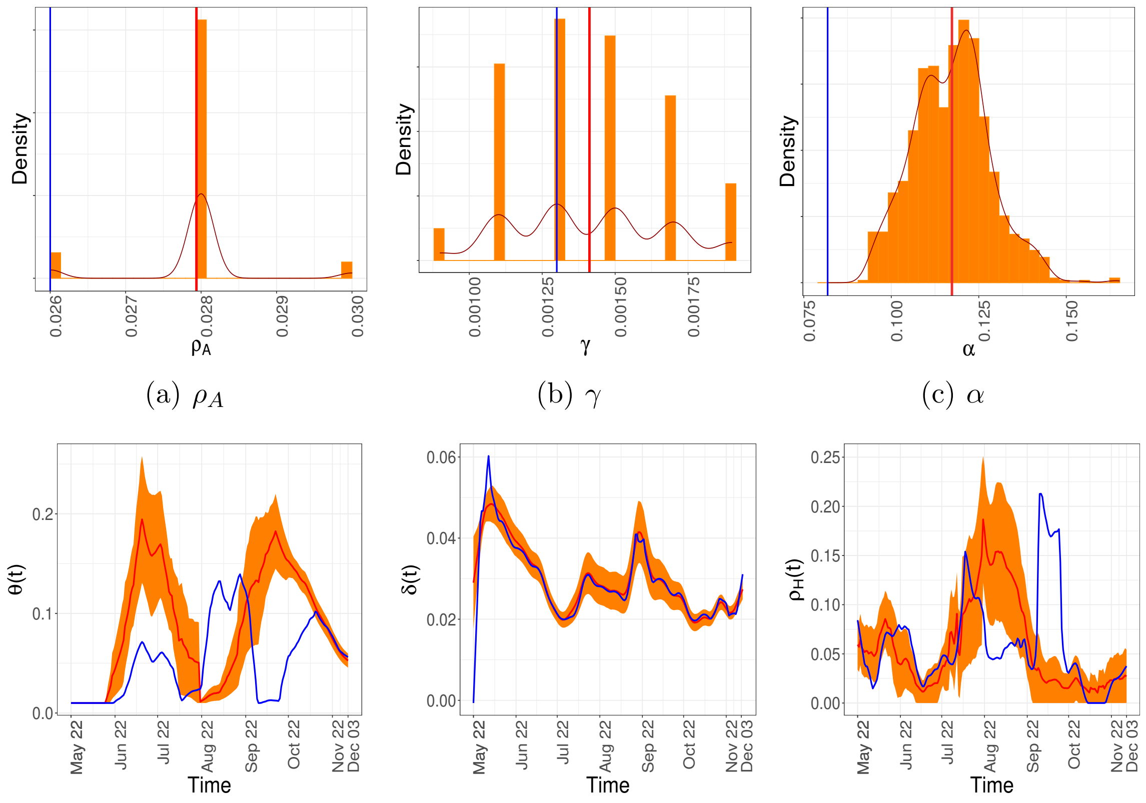

(a) ρ_A (b) γ (c) α

(d) $\theta(t)$ (e) $\delta(t)$ (f) $\rho_H(t)$

FIGURE 27. Estimates and residual bootstrap based confidence intervals for time invariant and time-varying parameters for the state of *Pennsylvania*. The estimate from the data is in blue. The 95% confidence band is in yellow and the mean of the bootstrap estimates are presented in red.

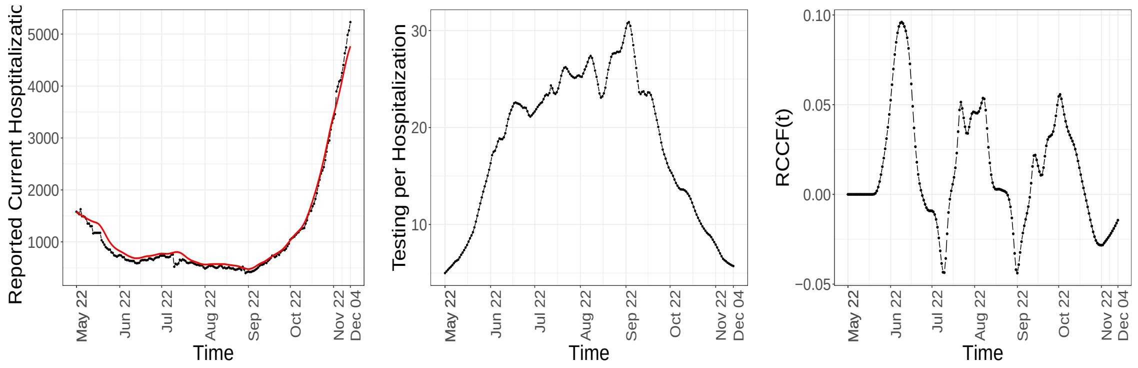

(a) Daily hospitalizations, observed (–), fitted (–)

(b) $\Delta T_t/H_t$,

(c) RCCF

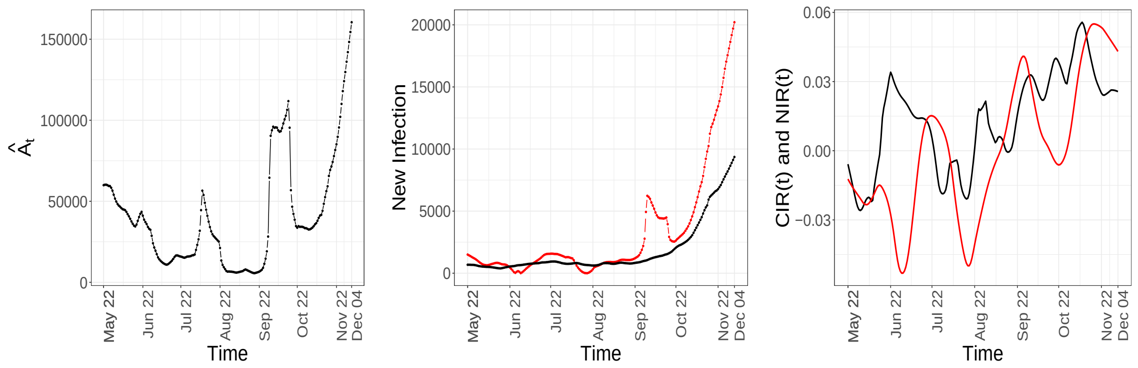

(d) $\hat{A}_t$

(e) Daily new infections, observed (–), fitted (–)

(f) CIR (–) and NIR (–)

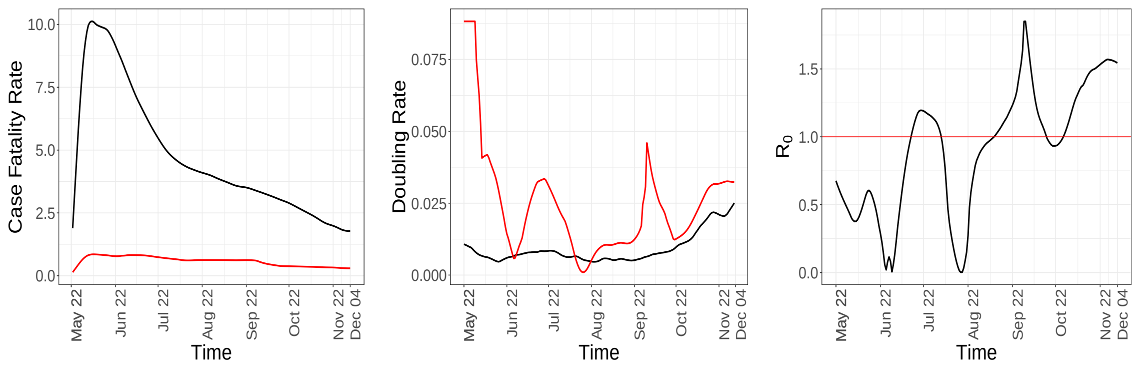

(g) Fatality Rate, observed (–), actual (–)

(h) Doubling rate, C_t (–) and CNI (–)

(i) R_0 analogue

FIGURE 28. Temporal patterns of some components and epidemiological markers for *Pennsylvania*.

South Dakota

	Estimate	95% Confidence Interval	Mean	s.d.
γ	0.0021	[0.0011, 0.0023]	0.0017	0.0003
ρ_A	0.0580	[0.0580, 0.0740]	0.0647	0.0042
α	0.0561	[0.0567, 0.0885]	0.0656	0.0075

Table 12. Confidence intervals, mean and standard deviations for the time-invariant parameters, computed based on 1000 bootstrap samples using residual bootstrap approach for *South Dakota*.

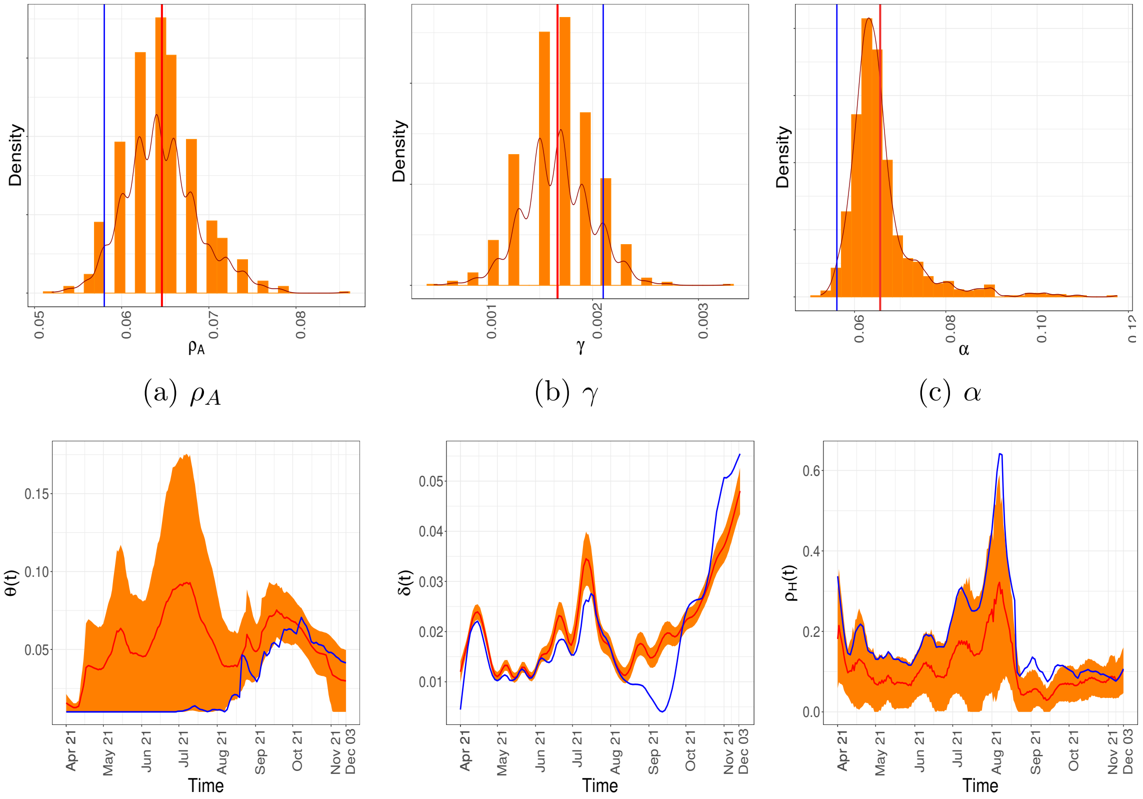

(a) ρ_A (b) γ (c) α

(d) $\theta(t)$ (e) $\delta(t)$ (f) $\rho_H(t)$

Figure 29. Estimates and residual bootstrap based confidence intervals for time invariant and time-varying parameters for the state of *South Dakota*. The estimate from the data is in blue. The 95% confidence band is in yellow and the mean of the bootstrap estimates are presented in red.

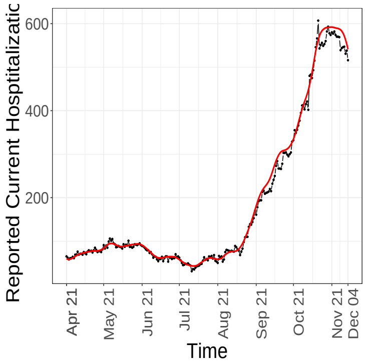

(a) Daily hospitalizations, observed (−), fitted (−)

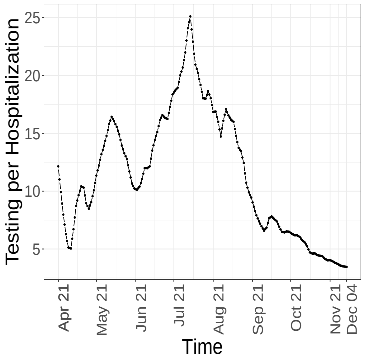

(b) $\Delta T_t/H_t$,

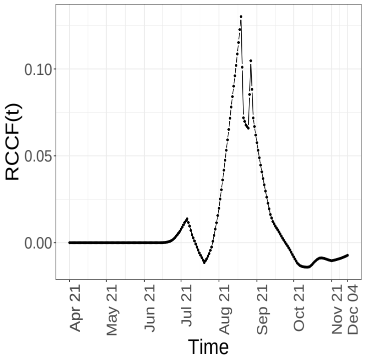

(c) RCCF

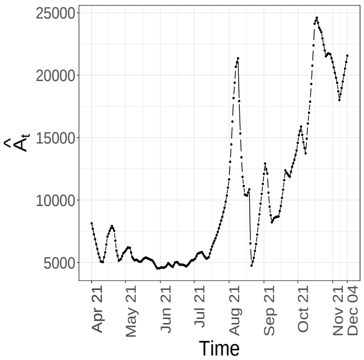

(d) $\hat{A}_t$

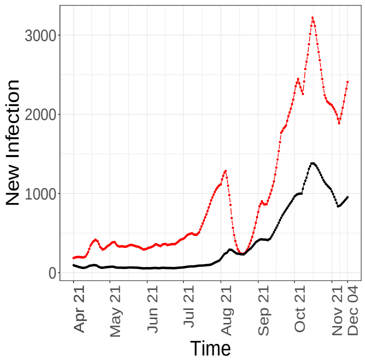

(e) Daily new infections, observed (−), fitted (−)

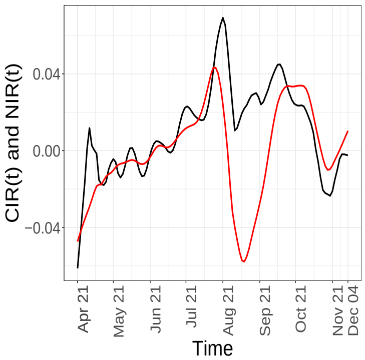

(f) CIR (−) and NIR (−)

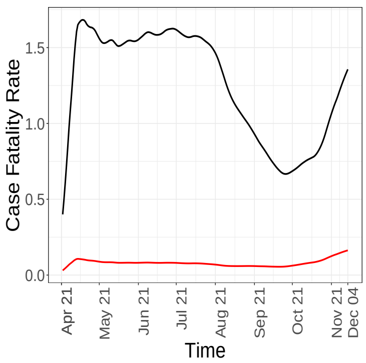

(g) Fatality Rate, observed (−), actual (−)

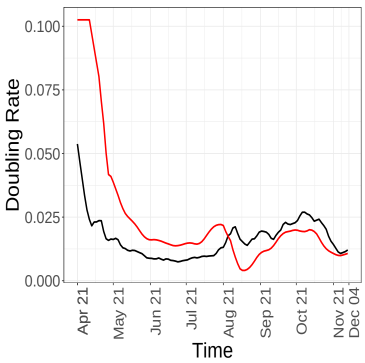

(h) Doubling rate, C_t (−) and CNI (−)

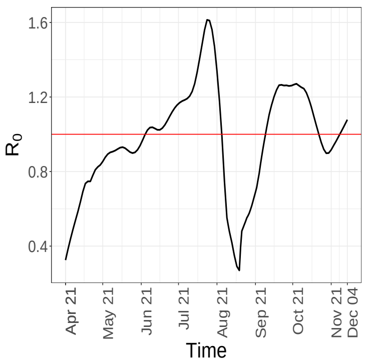

(i) R_0 analogue

FIGURE 30. Temporal patterns of some components and epidemiological markers for *South Dakota*.

Tennessee

	Estimate	95% Confidence Interval	Mean	s.d.
γ	0.0059	[0.0001, 0.0299]	0.0160	0.0084
ρ_A	0.0640	[0.0440, 0.0104]	0.0694	0.0154
α	0.1907	[0.0980, 0.2116]	0.1593	0.0301

TABLE 13. Confidence intervals, mean and standard deviations for the time-invariant parameters, computed based on 1000 bootstrap samples using residual bootstrap approach for *Tennessee*.

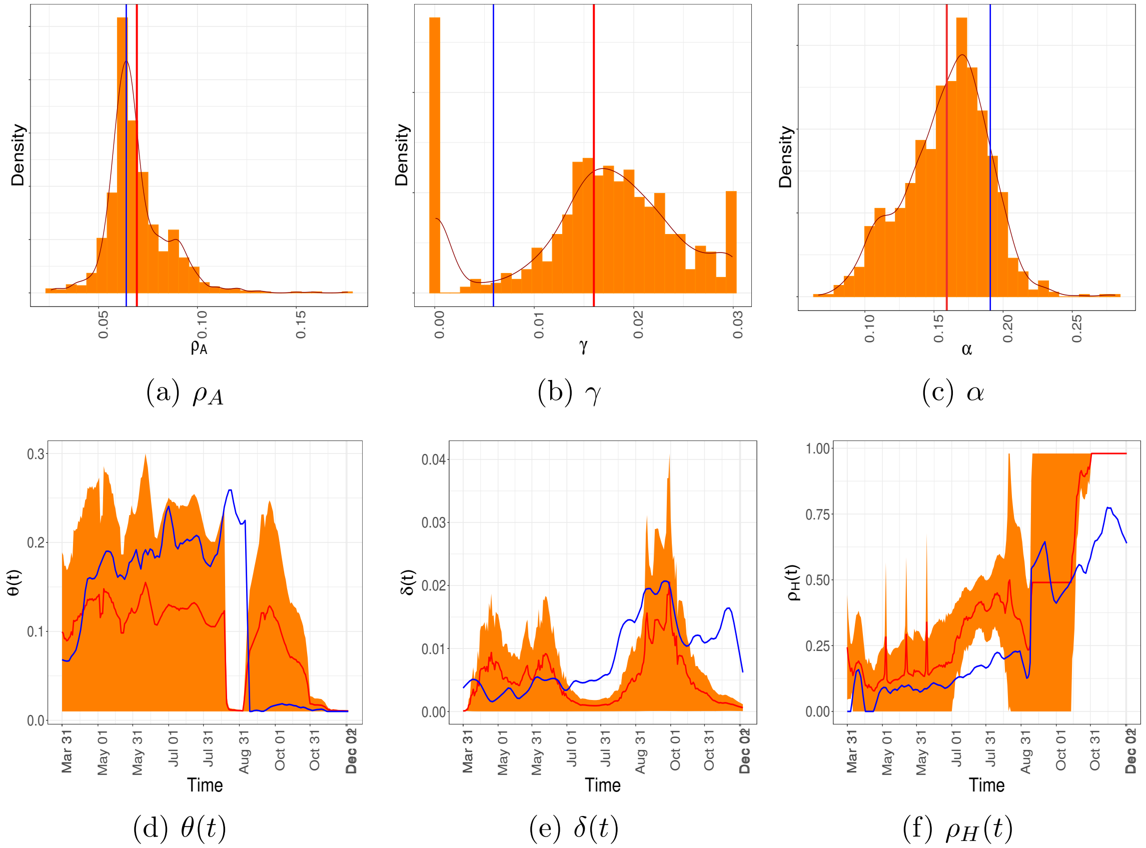

(a) ρ_A (b) γ (c) α

(d) $\theta(t)$ (e) $\delta(t)$ (f) $\rho_H(t)$

FIGURE 31. Estimates and residual bootstrap based confidence intervals for time invariant and time-varying parameters for the state of *Tennessee*. The estimate from the data is in blue. The 95% confidence band is in yellow and the mean of the bootstrap estimates are presented in red.

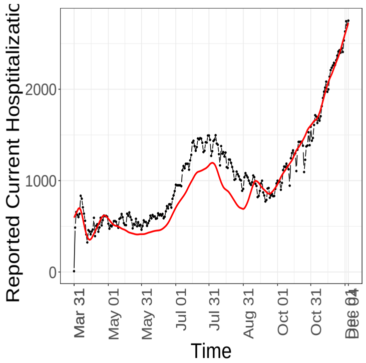

(a) Daily hospitalizations, observed (–), fitted (–)

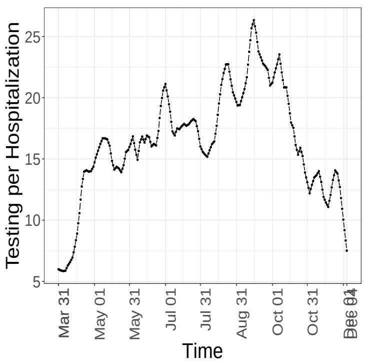

(b) $\Delta T_t/H_t$,

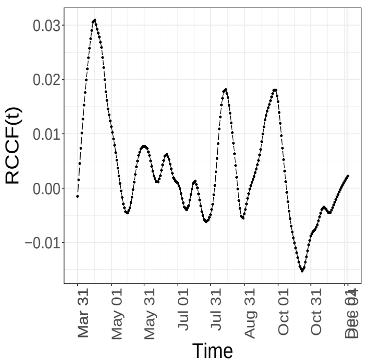

(c) RCCF

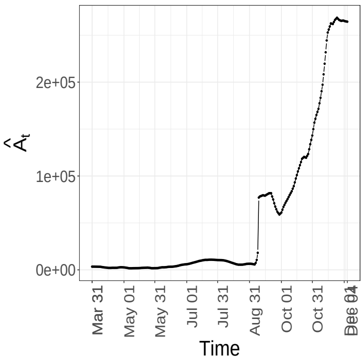

(d) $\hat{A}_t$

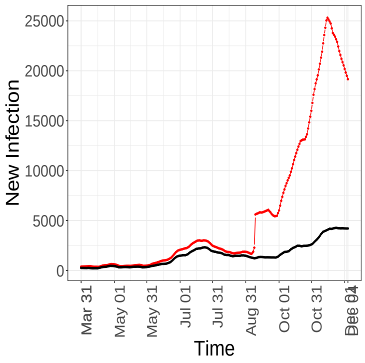

(e) Daily new infections, observed (–), fitted (–)

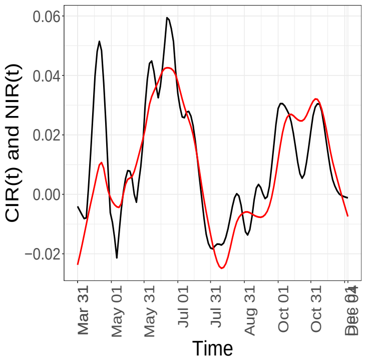

(f) CIR (–) and NIR (–)

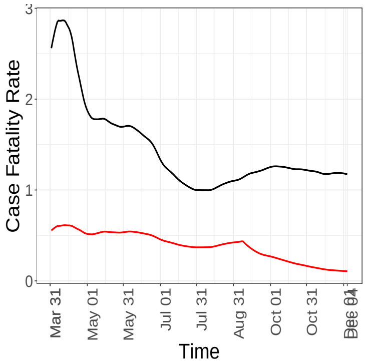

(g) Fatality Rate, observed (–), actual (–)

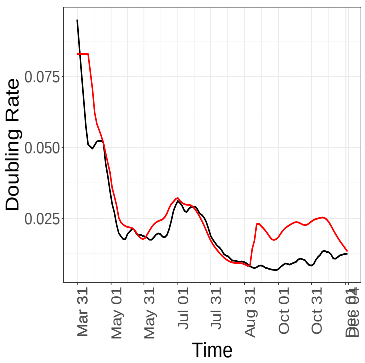

(h) Doubling rate, C_t (–) and CNI (–)

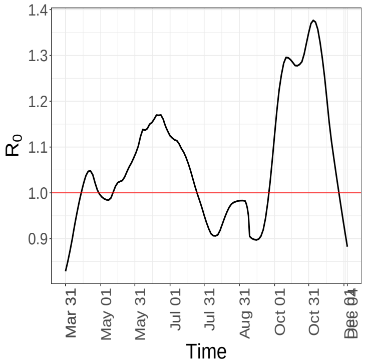

(i) R_0 analogue

FIGURE 32. Temporal patterns of some components and epidemiological markers for *Tennessee*.

Texas

	Estimate	95% Confidence Interval	Mean	s.d.
γ	0.0019	[0.0019, 0.0027]	0.0023	0.0003
ρ_A	0.0360	[0.0320, 0.0360]	0.0348	0.0013
α	0.0986	[0.1021, 0.1583]	0.1325	0.0142

Table 14. Confidence intervals, mean and standard deviations for the time-invariant parameters, computed based on 1000 bootstrap samples using residual bootstrap approach for *Texas*.

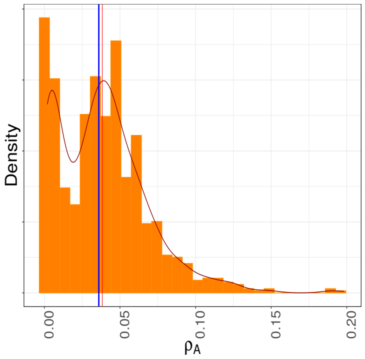

(a) ρ_A

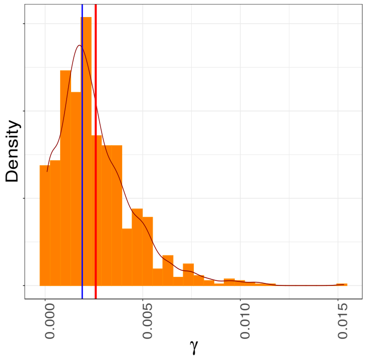

(b) γ

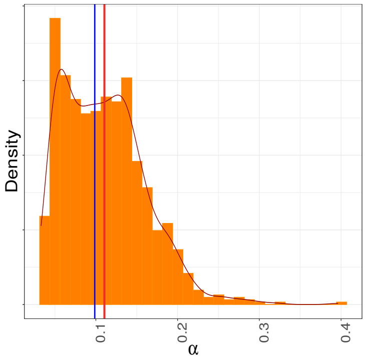

(c) α

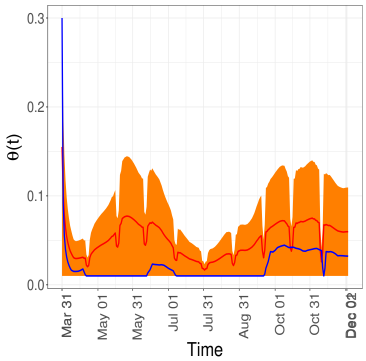

(d) $\theta(t)$

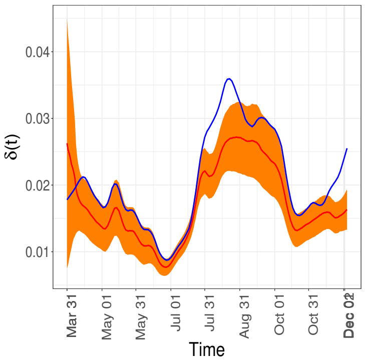

(e) $\delta(t)$

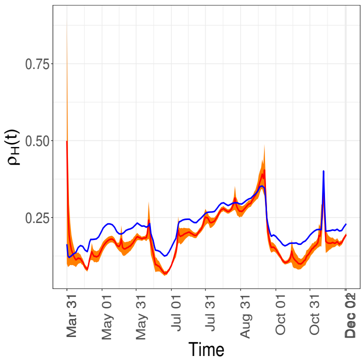

(f) $\rho_H(t)$

Figure 33. Estimates and residual bootstrap based confidence intervals for time invariant and time-varying parameters for the state of *Texas*. The estimate from the data is in blue. The 95% confidence band is in yellow and the mean of the bootstrap estimates are presented in red.

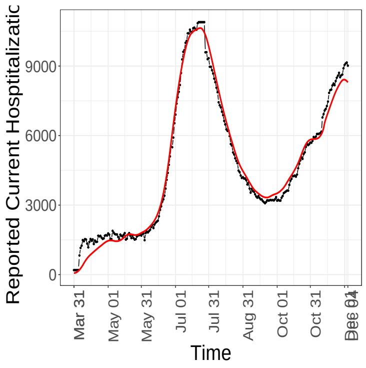

(a) Daily hospitalizations, observed (–), fitted (–)

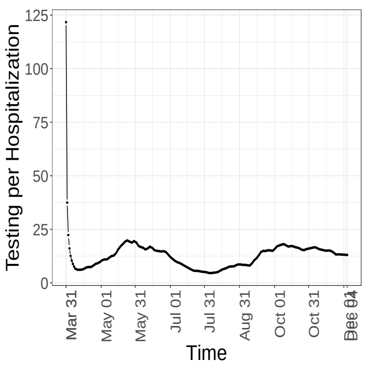

(b) $\Delta T_t / H_t$,

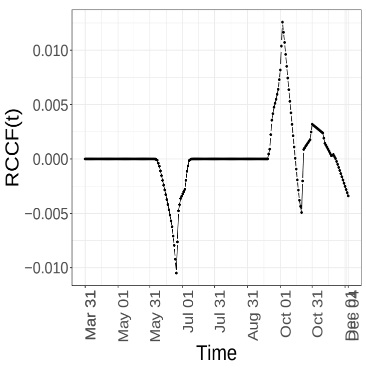

(c) RCCF

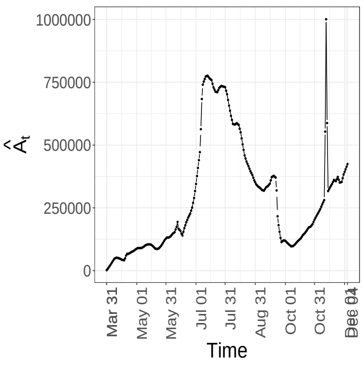

(d) $\hat{A}_t$

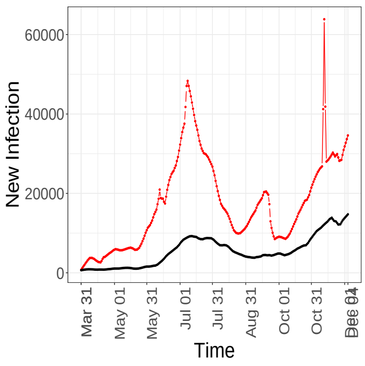

(e) Daily new infections, observed (–), fitted (–)

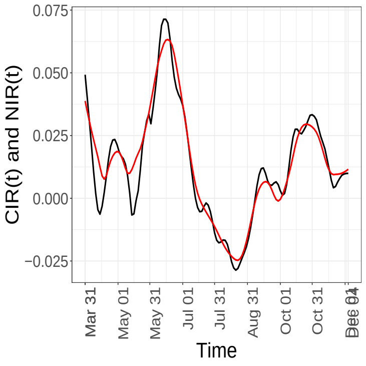

(f) CIR (–) and NIR (–)

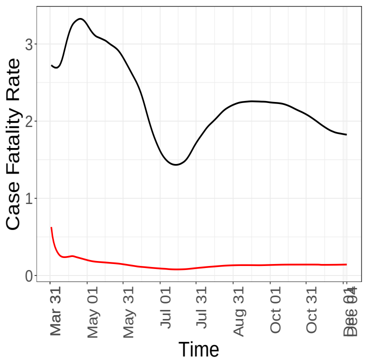

(g) Fatality Rate, observed (–), actual (–)

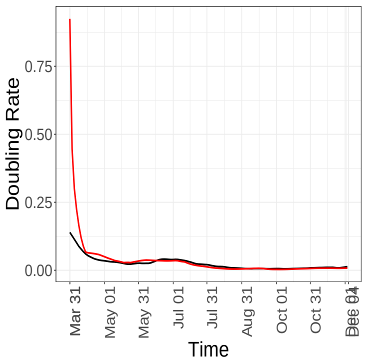

(h) Doubling rate, C_t (–) and CNI (–)

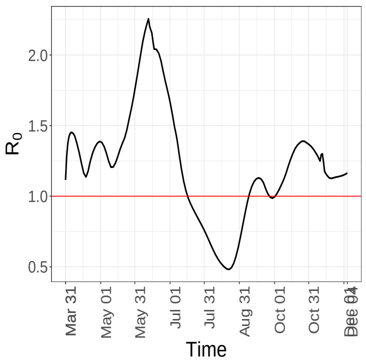

(i) R_0 analogue

FIGURE 34. Temporal patterns of some components and epidemiological markers for *Texas*.

Wisconsin

	Estimate	95% Confidence Interval	Mean	s.d.
γ	0.0017	[0.0005, 0.0015]	0.0010	0.0003
ρ_A	0.0680	[0.0580, 0.0700]	0.0644	0.0028
α	0.0926	[0.0801, 0.1312]	0.1097	0.0130

TABLE 15. Confidence intervals, mean and standard deviations for the time-invariant parameters, computed based on 1000 bootstrap samples using residual bootstrap approach for *Wisconsin*.

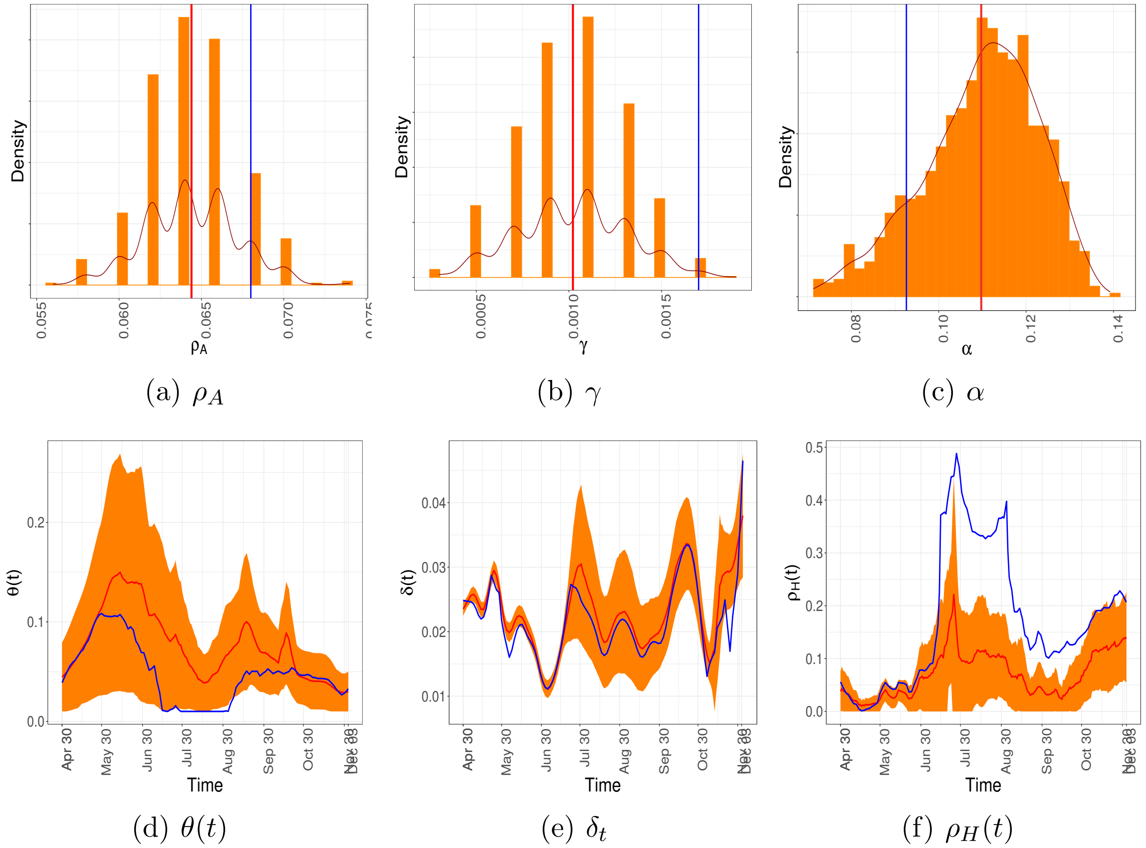

(a) ρ_A (b) γ (c) α

(d) $\theta(t)$ (e) δ_t (f) $\rho_H(t)$

FIGURE 35. Estimates and residual bootstrap based confidence intervals for time invariant and time-varying parameters for the state of *Wisconsin*. The estimate from the data is in blue. The 95% confidence band is in yellow and the mean of the bootstrap estimates are presented in red.

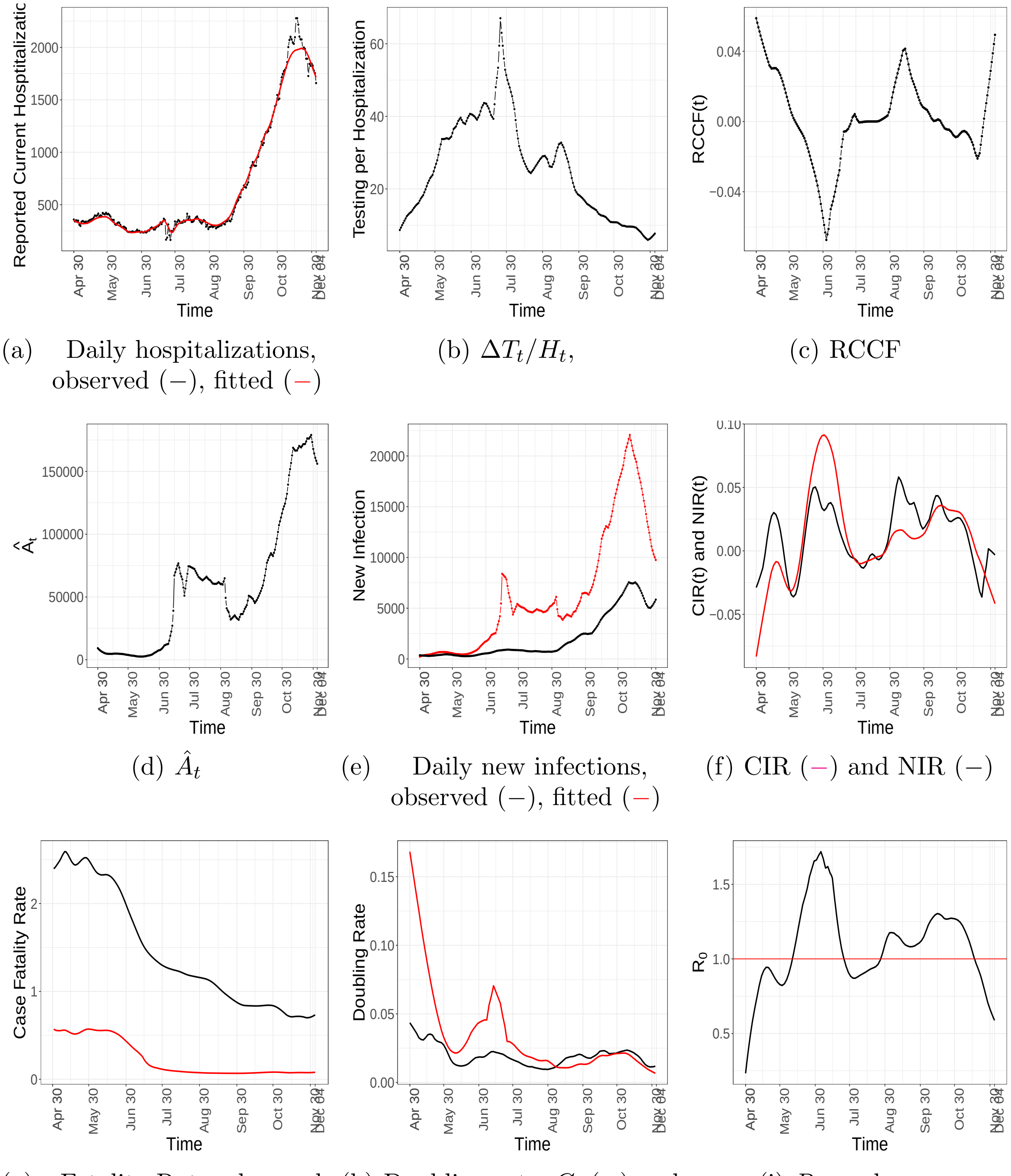

(a) Daily hospitalizations, observed (–), fitted (–)

(b) $\Delta T_t/H_t$,

(c) RCCF

(d) $\hat{A}_t$

(e) Daily new infections, observed (–), fitted (–)

(f) CIR (–) and NIR (–)

(g) Fatality Rate, observed (–), actual (–)

(h) Doubling rate, C_t (–) and CNI (–)

(i) R_0 analogue

FIGURE 36. Temporal patterns of some components and epidemiological markers for *Wisconsin*.

References

[1] IHME COVID-19 forecasting team. Modeling COVID-19 scenarios for the United States. Nature Medicine, 2020.

[2] Hemant Bherwani, Saima Anjum, Suman Kumar, Sneha Gautam, Ankit Gupta, Himanshu Kumbhare, Avneesh Anshul, and Rakesh Kumar. Understanding COVID-19 transmission through bayesian probabilistic modeling and gis-based voronoi approach: a policy perspective. Environment, Development and Sustainability, pages 1–19, 2020.

[3] Gergo Pinter, Imre Felde, Amir Mosavi, Pedram Ghamisi, and Richard Gloaguen. COVID-19 pandemic prediction for hungary; a hybrid machine learning approach. Mathematics, 8(6):890, 2020.

[4] Narinder Singh Punn, Sanjay Kumar Sonbhadra, and Sonali Agarwal. COVID-19 epidemic analysis using machine learning and deep learning algorithms. MedRxiv, 2020.
